# Supplementary material for: Nitrogen dioxide pollution in 346 Chinese cities: Spatiotemporal variations and natural drivers from multi-source remote sensing data
Source: PLoS One. 2025 Nov 7;20(11):e0334535. doi: 10.1371/journal.pone.0334535 (PMC12594365; doi:10.1371/journal.pone.0334535)
Supplement: S1 File — This text presents the optimal parameters obtained after tuning the random forest using the particle swarm optimization algorithm. (PDF) [file pone.0334535.s001.pdf]

Stopping search: Swarm best objective change less than 1e-08
optimal parameter: n_estimators=4953, max_depth=18, min_samples_split=4, min_samples_leaf=1,
training set accuracy： 0.9427572073600595 3.793265149187732e-10 1.1530369285556027e-05 1.9476306500945532e-05 0.9427169166277293
test set accuracy： 0.7644377776510662 1.2206709470696028e-09 2.3486978912545244e-05 3.493810165234515e-05 0.7630375550738906
