# Supplementary material for: Nitrogen dioxide pollution in 346 Chinese cities: Spatiotemporal variations and natural drivers from multi-source remote sensing data
Source: PLoS One. 2025 Nov 7;20(11):e0334535. doi: 10.1371/journal.pone.0334535 (PMC12594365; doi:10.1371/journal.pone.0334535)
Supplement: S2 File — This document contains the code for the parameter tuning process of the Particle Swarm Optimization (PSO) algorithm. (DOCX) [file pone.0334535.s002.docx]

import pandas as pd

import numpy as np

from sklearn.ensemble import RandomForestRegressor

from sklearn.metrics import explained_variance_score, mean_squared_error, mean_absolute_error, r2_score

from pyswarm import pso

def cal_score(y_true, y_pred):

ev = explained_variance_score(y_true, y_pred)

mse = mean_squared_error(y_true, y_pred)

mae = mean_absolute_error(y_true, y_pred)

rmse = np.sqrt(mse)

r2 = r2_score(y_true, y_pred)

return ev, mse, mae, rmse, r2

def rf_model_score(params):

n_estimators = int(params[0])

max_depth = int(params[1])

min_samples_split = int(params[2])

min_samples_leaf = int(params[3])

# max_features

max_features = 1.0

reg_model = RandomForestRegressor(

n_estimators=n_estimators,

max_depth=max_depth,

min_samples_split=min_samples_split,

min_samples_leaf=min_samples_leaf,

max_features=max_features,

random_state=100

)

reg_model.fit(train_X, train_Y)

ptrain = reg_model.predict(train_X)

_, mse_train, _, _, _ = cal_score(train_Y, ptrain)

return mse_train

data_train = pd.read_excel("C:\\Users\\Administrator\\Desktop\\Train.xlsx")

data_test = pd.read_excel("C:\\Users\\Administrator\\Desktop\\Test.xlsx")

train_X = data_train.iloc[:, 1:].values

train_Y = data_train.iloc[:, 0].values

test_X = data_test.iloc[:, 1:].values

test_Y = data_test.iloc[:, 0].values

lb = [10, 5, 2, 1]

ub = [1000, 30, 20, 20]

best_params, best_score = pso(rf_model_score, lb, ub, swarmsize=20, maxiter=50)

best_n_estimators = int(best_params[0])

best_max_depth = int(best_params[1])

best_min_samples_split = int(best_params[2])

best_min_samples_leaf = int(best_params[3])

print(f"optimal parameters: n_estimators={best_n_estimators}, max_depth={best_max_dep
