# Supplementary material for: Nitrogen dioxide pollution in 346 Chinese cities: Spatiotemporal variations and natural drivers from multi-source remote sensing data
Source: PLoS One. 2025 Nov 7;20(11):e0334535. doi: 10.1371/journal.pone.0334535 (PMC12594365; doi:10.1371/journal.pone.0334535)
Supplement: S3 File — This document contains the code for the SHAP explanation algorithm, which is used to analyze the response degree of various influencing factors to NO2 concentration. (DOCX) [file pone.0334535.s003.docx]

import shap

import matplotlib.pyplot as plt

import pandas as pd

import xgboost as xgb

from sklearn.preprocessing import LabelEncoder

import numpy as np

train_data = pd.read_excel("C:\\Users\\Administrator\\Desktop\\test\\Kumar.xlsx")

print("Data loaded successfully.")

label_encoder = LabelEncoder()

train_data.iloc[:, 0] = label_encoder.fit_transform(train_data.iloc[:, 0])

X_train = train_data.iloc[:, 1:]

y_train = train_data.iloc[:, 0]

X_train = pd.get_dummies(X_train, drop_first=True)

xgb_model = xgb.XGBClassifier(n_estimators=100, max_depth=10, random_state=42)

xgb_model.fit(X_train, y_train)

explainer = shap.TreeExplainer(xgb_model)

shap_values = explainer(X_train)

shap_interaction_values = explainer.shap_interaction_values(X_train)

# print("shap_values")

# print(shap_values)

# print("shap_interaction_values")

# print(shap_interaction_values)

shap.summary_plot(shap_values, X_train, plot_type="bar")

# shap.summary_plot(shap_values, X_train, plot_type="dot")

shap.summary_plot(shap_interaction_values, X_train)

# shap.plots.force(shap_values[8][:, 1])

#shap.plots.force(explainer.expected_value[1], shap_values2[1][:80],feature_names=feature_names)

shap.plots.waterfall(shap_values[8])
