# Supplementary material for: Nitrogen dioxide pollution in 346 Chinese cities: Spatiotemporal variations and natural drivers from multi-source remote sensing data
Source: PLoS One. 2025 Nov 7;20(11):e0334535. doi: 10.1371/journal.pone.0334535 (PMC12594365; doi:10.1371/journal.pone.0334535)
Supplement: Attachment — Submitted filename: PONE-D-25-30016_reviewer.pdf [file pone.0334535.s006.pdf]

PLOS ONE

Spatiotemporal Dynamics and Multidimensional Drivers of Nitrogen dioxide concentration Pollution in 346 Chinese Cities: A Remote Sensing Data Fusion Approach

--Manuscript Draft--

**Manuscript Number:**	PONE-D-25-30016
**Article Type:**	Research Article
**Full Title:**	Spatiotemporal Dynamics and Multidimensional Drivers of Nitrogen dioxide concentration Pollution in 346 Chinese Cities: A Remote Sensing Data Fusion Approach
**Short Title:**	Unveiling Nitrogen dioxide Pollution Dynamics and Drivers in 346 Chinese Cities with Remote Sensing Fusion
**Corresponding Author:**	Heming Yang<br>Jilin Normal University<br>Siping City, Jilin Province CHINA
**Keywords:**	Sentinel-5P; Nitrogen dioxide column concentrations; spatiotemporal distribution characteristics; Random Forest model; SHapley Additive exPlanations
**Abstract:**	In this study, we obtained tropospheric nitrogen dioxide concentration data based on Sentinel-5P data. We also employed statistical and local spatial autocorrelation analyses to investigate the spatial-temporal distribution and variation of nitrogen dioxide concentration in 346 major Chinese cities from 2019 to 2023. The Random Forest (RF) and Shapley Additive Explanations (SHAP) were utilized to analyze the influence of 15 factors to nitrogen dioxide in the air, such as pressure, leaf area index, ~~and etc.~~ Additionally, partial dependence plots were introduced to investigate the proportional relationship between a single factor and pollutants. ~~The results show that~~ nitrogen dioxide concentration in the 346 cities varies with seasons, with the highest concentration in winter and the lowest in summer. Nitrogen dioxide concentration exhibited an increasing, then decreasing, and increasing again trend from 2019 to 2023. It is higher in capital cities and in northern and northeastern areas of China than in southern, southwestern, and northwestern areas of China. ~~The results also show that~~ nitrogen dioxide concentration is significantly affected by temperature-related factors. ~~Nitrogen dioxide concentration is also affected by~~ aerosol optical thickness and leaf area index. Our findings can help inform policymaking and pollution remediation.
**Order of Authors:**	Feifei Cheng
	Qing Sun
	Jiaqi Zhang
	Jiahe Liu
	Wenwen Lü
	Fapeng Shen
	Heming Yang
**Additional Information:**	
**Question**	**Response**
**Financial Disclosure**<br><br>Enter a financial disclosure statement that describes the sources of funding for the work included in this submission. Review the submission guidelines for detailed requirements. View published research	Yes

articles from *PLOS ONE* for specific examples.<br><br>This statement is required for submission and **will appear in the published article** if the submission is accepted. Please make sure it is accurate.<br><br>**Funded studies**<br>Enter a statement with the following details:<br>• Initials of the authors who received each award<br>• Grant numbers awarded to each author<br>• The full name of each funder<br>• URL of each funder website<br>• Did the sponsors or funders play any role in the study design, data collection and analysis, decision to publish, or preparation of the manuscript?<br><br>Did you receive funding for this work?	
Please add funding details.<br>as follow-up to "**Financial Disclosure**<br><br>Enter a financial disclosure statement that describes the sources of funding for the work included in this submission. Review the submission guidelines for detailed requirements. View published research articles from *PLOS ONE* for specific examples.<br><br>This statement is required for submission and **will appear in the published article** if the submission is accepted. Please make sure it is accurate.	Funding: This research was funded by the Jilin Provincial Department of Science and Technology, Approval No.: YDZJ202401520ZYTS.

**Funded studies**<br>Enter a statement with the following details:<br>• Initials of the authors who received each award<br>• Grant numbers awarded to each author<br>• The full name of each funder<br>• URL of each funder website<br>• Did the sponsors or funders play any role in the study design, data collection and analysis, decision to publish, or preparation of the manuscript?<br><br>Did you receive funding for this work?"	
Please select the country of your main research funder (please select carefully as in some cases this is used in fee calculation).<br>as follow-up to "**Financial Disclosure**<br><br>Enter a financial disclosure statement that describes the sources of funding for the work included in this submission. Review the submission guidelines for detailed requirements. View published research articles from *PLOS ONE* for specific examples.<br><br>This statement is required for submission and **will appear in the published article** if the submission is accepted. Please make sure it is accurate.<br><br>**Funded studies**<br>Enter a statement with the following details:<br>• Initials of the authors who received each award<br>• Grant numbers awarded to each author<br>• The full name of each funder<br>• URL of each funder website<br>• Did the sponsors or funders play any role in the study design, data collection and analysis, decision to publish, or preparation of the manuscript?<br><br>Did you receive funding for this work?"	CHINA - CN
**Competing Interests**	The authors have declared that no competing interests exist.

Use the instructions below to enter a competing interest statement for this submission. On behalf of all authors, disclose any competing interests that could be perceived to bias this work—acknowledging all financial support and any other relevant financial or non-financial competing interests.<br><br>This statement is required for submission and **will appear in the published article** if the submission is accepted. Please make sure it is accurate and that any funding sources listed in your Funding Information later in the submission form are also declared in your Financial Disclosure statement.<br><br>View published research articles from *PLOS ONE* for specific examples.<br><br>**NO authors have competing interests**<br><br>Enter: *The authors have declared that no competing interests exist.*<br><br>**Authors with competing interests**<br><br>Enter competing interest details beginning with this statement:<br><br>*I have read the journal's policy and the authors of this manuscript have the following competing interests: [insert competing interests here]*<br><br>* typeset	
**Ethics Statement**<br><br>Enter an ethics statement for this submission. This statement is required if the study involved:<br><br>• Human participants<br>• Human specimens or tissue<br>• Vertebrate animals or cephalopods<br>• Vertebrate embryos or tissues	N/A

Powered by Editorial Manager® and ProduXion Manager® from Aries Systems Corporation

• Field research<br><br>Write "N/A" if the submission does not require an ethics statement.<br><br>General guidance is provided below. Consult the submission guidelines for detailed instructions. **Make sure that all information entered here is included in the Methods section of the manuscript.**	

**Format for specific study types**<br><br>**Human Subject Research (involving human participants and/or tissue)**<br>• Give the name of the institutional review board or ethics committee that approved the study<br>• Include the approval number and/or a statement indicating approval of this research<br>• Indicate the form of consent obtained (written/oral) or the reason that consent was not obtained (e.g. the data were analyzed anonymously)<br><br>**Animal Research (involving vertebrate animals, embryos or tissues)**<br>• Provide the name of the Institutional Animal Care and Use Committee (IACUC) or other relevant ethics board that reviewed the study protocol, and indicate whether they approved this research or granted a formal waiver of ethical approval<br>• Include an approval number if one was obtained<br>• If the study involved *non-human primates*, add *additional details* about animal welfare and steps taken to ameliorate suffering<br>• If anesthesia, euthanasia, or any kind of animal sacrifice is part of the study, include briefly which substances and/or methods were applied<br><br>**Field Research**<br><br>Include the following details if this study involves the collection of plant, animal, or other materials from a natural setting:<br>• Field permit number<br>• Name of the institution or relevant body that granted permission	
**Data Availability**<br><br>Authors are required to make all data underlying the findings described fully available, without restriction, and from the time of publication. PLOS allows rare exceptions to address legal and ethical concerns. See the PLOS Data Policy and FAQ for detailed information.	Yes - all data are fully available without restriction

A Data Availability Statement describing where the data can be found is required at submission. Your answers to this question constitute the Data Availability Statement and **will be published in the article**, if accepted.<br><br>**Important:** Stating 'data available on request from the author' is not sufficient. If your data are only available upon request, select 'No' for the first question and explain your exceptional situation in the text box.<br><br>Do the authors confirm that all data underlying the findings described in their manuscript are fully available without restriction?	
**Describe where the data may be found in full sentences. If you are copying our sample text, replace any instances of XXX with the appropriate details.**<br><br>• If the data are **held or will be held in a public repository**, include URLs, accession numbers or DOIs. If this information will only be available after acceptance, indicate this by ticking the box below. For example: *All XXX files are available from the XXX database (accession number(s) XXX, XXX.).*<br>• If the data are all contained **within the manuscript and/or Supporting Information files**, enter the following: *All relevant data are within the manuscript and its Supporting Information files.*<br>• If neither of these applies but you are able to provide **details of access elsewhere**, with or without limitations, please do so. For example:<br><br>*Data cannot be shared publicly because of [XXX]. Data are available from the XXX Institutional Data Access / Ethics Committee (contact via XXX) for researchers who meet the criteria for access to confidential data.*<br><br>*The data underlying the results presented in the study are available from (include the name of the third party*	The data underlying the results presented in the study are available from the Google Earth Engine. https://developers.google.cn › earth-engine

and contact information or URL).<br>• This text is appropriate if the data are owned by a third party and authors do not have permission to share the data.<br><br>* typeset	
Additional data availability information:	

Manuscript

Click here to access/download;Manuscript;MainText_Yang_20250605.docx

Spatiotemporal Dynamics and Multidimensional Drivers of Nitrogen dioxide concentration Pollution in 346 Chinese Cities: A Remote Sensing Data Fusion Approach

Feifei Cheng [a, ¶], Qing Sun [b, ¶], Jiaqi Zhang [a], Jiahe Liu [b], Wenwen Lü [a], Fapeng Shen [b], Heming Yang [a, *]

[a]*Jilin Normal University, Siping, Jilin, China;*

[b]*Jilin Agricultural University, Changchun, Jilin, China.*

[¶]These authors contributed equally to this study.

*Corresponding author at: E-mail addresses: wosyhm@jlnu.edu.cn (H. Yang).

Abstract:

In this study, we obtained tropospheric nitrogen dioxide concentration data based on Sentinel-5P data. We also employed statistical and local spatial autocorrelation analyses to investigate the spatial-temporal distribution and variation of nitrogen dioxide concentration in 346 major Chinese cities from 2019 to 2023. The Random Forest (RF) and Shapley Additive Explanations (SHAP) were utilized to analyze the influence of 15 factors to nitrogen dioxide in the air, such as pressure, leaf area index, ~~and etc.~~ Additionally, partial dependence plots were introduced to investigate the proportional relationship between a single factor and pollutants. The results show that nitrogen dioxide concentration in the 346 cities varies with seasons, with the highest concentration in winter and the lowest in summer. Nitrogen dioxide concentration exhibited an increasing, then decreasing,

and increasing again trend from 2019 to 2023. It is higher in capital cities and in northern and northeastern areas of China than in southern, southwestern, and northwestern areas of China. The results also show that nitrogen dioxide concentration is significantly affected by temperature-related factors. Nitrogen dioxide concentration is also affected by aerosol optical thickness and leaf area index. Our findings can help inform policymaking and pollution remediation.

Keywords: Sentinel-5P; Nitrogen dioxide column concentrations; spatiotemporal distribution characteristics; Random Forest model; Shapley Additive Explanations

1. Introduction

In recent years, environmental problems caused by air pollutants have attracted more and more attention [1]. Air pollution, which threats human health and eco-systems is a global environmental issue [2]. Air quality in any region is directly influenced by the local population and their activities [3]. Nitrogen dioxide is one of the principal pollutants worldwide because of its potential to become a toxic gas associated with several adverse health effects related to respiratory irritation [4]. The concentration of NO_2 is an important indicator to air pollution [5], because NO_2 leads to acid rain, acid fog, and photochemical smog [6], increases $PM_{2.5}$ concentrations [7-8], threats public health [9-12], and harms society and ecological environment [13]. Therefore, it is important to investigate air NO_2 pollution [14].

To systematically investigate the etiology of atmospheric pollution and develop source-oriented mitigation strategies, scholars have predominantly focused on two key research thrusts: (1) the spatiotemporal variations of the air pollution and (2) the identification of multi-scale drivers influencing pollutant dynamics [15]. Traditional ground-based air pollution monitoring and analysis method is sometimes unrepresentative because of (1) insufficient samples across spatial and temporal dimensions [16], and (2) the uncertainty of influence from other gases [17]. Satellite data, combined with ground-based meteorological information and advanced machine learning, can generate

reliable information on gas pollutant concentration (Jesus Rodrigo Cedeno Jimenez). Random Forest (RF), a common Machine Learning (ML) method, extracts discriminative features from high-dimensional datasets, serving as a more robust tool for pollution analysis [18].

Previous literatures have proposed several factors that may influence air pollution. For example, Wang et al. (2015) found that atmospheric pollutants in Wuxi's urban area during 2014 exhibited strong correlations with meteorological parameters including temperature, relative humidity, wind speed, and precipitation [19]. Liu et al. found that the level of urbanization and the intensity of human activities in Fangshan District, Beijing were significantly positively correlated with the concentration of environmental pollutants [20]. Other factors, such as vegetation coverage index [21], were also investigated by many scholars [22-30]. These factors have universality. Building upon these foundations, 15 independent variables were selected for studying NO_2 concentration based on principles of data representativeness, quantifiability, and accessibility, while considering the interconnected systemic effects of multiple factors. These variables include Pressure (atmospheric pressure), Lai (Leaf Area Index), Dew (dew point temperature), AOD (aerosol optical depth), WS (wind speed), Month (month), ET (evapotranspiration), RH (relative humidity), Pre (precipitation), Fire (fire activity), LST (land sur-face

temperature), Temp (air temperature), GPP (gross primary productivity), Year (year), and Snow (snow cover).

This study leveraged Google Earth Engine (GEE) platform to retrieve tropospheric NO_2 column concentration (TNO2CC) data, integrating a particle swarm optimization-enhanced random forest with Shapley Additive Explanations interpretation algorithms to systematically analyze the spatiotemporal variation patterns and driving factors of NO_2 pollution across China's prefecture-level cities. Building upon this framework, we quantitatively investigated the impacts of meteorological drivers on regional air quality dynamics. The methodological integration of satellite remote sensing and explainable machine learning provides a robust analytical paradigm for identifying pollution control priorities, thereby establishing a theoretical foundation for evidence-based atmospheric pollution mitigation strategies in China.

2. Materials and Methods

2.1 Study area and data sources

As the world's largest developing country, China covers vast territorial expanses and encompasses diverse natural environments. The nation extends across extensive latitudinal gradients and traverses multiple climatic zones, including tropical, subtropical, temperate, and frigid regions. These distinct

climatic regimes exhibit significant variations in temperature, humidity, and wind patterns. For instance, southern cities experience humid subtropical conditions, while northern regions are characterized by continental aridity. China's landforms are complex and diverse, comprising plateaus, mountains, plains, basins, and other landform types. Mountainous and plateau regions facilitate air mass exchange, whereas basin and plain terrains restrict pollutant diffusion. Urban development patterns vary markedly across China's 346 study units, which include sub-provincial cities, prefecture-level municipalities, and autonomous prefectures. These urban centers, predominantly concentrated in eastern China, display divergent industrialization trajectories, urbanization rates, and population densities. The selected cities encapsulate representative variations in terrain, climatic, socioeconomic conditions, and environmental governance frameworks, ensuring a comprehensive analysis of regional atmospheric dynamics. Research manuscripts reporting large datasets that are deposited in a publicly available database should specify where the data have been deposited and provide the relevant accession numbers. If the accession numbers have not yet been obtained at the time of submission, please state that they will be provided during review. They must be provided prior to publication.

Figure 1. Distribution map of major cities in China.

The Sentinel satellite series is a key component of the European Union's Copernicus Earth Observation Program. The Sentinel-5 Precursor (S5P) satellite, launched in October 2017, specializes in near-real-time global monitoring of atmospheric trace gases, aerosols, and cloud distributions with unprecedented spatial resolution of 7 × 3.5 km at nadir. This study employed S5P's Offline (OFFL) processing products, specifically the "COPERNICUS_S5P_OFFL_L3_NO2" dataset, to quantify tropospheric NO_2 column densities. The Level 3 product provided gridded daily averages derived from the TROPOMI spectrometer measurements, enabling a systematic characterization of NO_2 pollution patterns across China's urban agglomerations.

Other data fetch is as follows：

Table 1. Data source for the study area.

Date	Source
Temp	ECMWF/ERA5_LAND/MONTHLY
LST	MODIS/061/MOD11A2
Pressure	ECMWF/ERA5_LAND/MONTHLY
WS	ECMWF/ERA5_LAND/MONTHLY
RH	ECMWF/ERA5_LAND/MONTHLY
Dew	ECMWF/ERA5_LAND/MONTHLY
Pre	ECMWF/ERA5_LAND/MONTHLY
ET	MODIS/006/MOD16A2
Snow	MODIS/061/MOD10A1
GPP	MODIS/061/MYD17A3HGF
AOD	MODIS/061/MOD08_M3
Lai	MODIS/061/MCD15A3H
Fire	MODIS/061/MOD14A1

Date is the data name, and Source is the Source of the data obtained.

The vector boundaries of urban built-up areas were extracted from the China urban built-up areas 2020 dataset. Using ArcMap 10.8, the geographic coordinates (latitude and longitude) of each city's built-up area centroid were calculated using spatial analysis tools.

2.2 Research methods

(1) Data preprocessing:

Remote sensing data processing: The geemap package was used to call and process datasets on the GEE platform in Python 3.8.

Sentinel-5P series: Remote sensing data varies in spatial and temporal resolution. Remote sensing data with a resolution of 1 km was uniformly exported through the GEE platform. Filter out low-quality pixels, mask parts less than 0, synthesize annual and quarterly means, and conduct raster data statistics one by one using the boundaries of built-up areas.

(2) Local spatial autocorrelation analysis: The local spatial autocorrelation method effectively detected spatial heterogeneity in urban atmospheric pollution patterns by identifying the geographic locations of pollution clusters and their aggregation types, such as high-high (HH) and low-low (LL) clusters. The calculation formulas were as follows:

$$I = \frac{(x_i - \bar{x})}{S^2} \sum_{j = 1, j \neq i}^{n} W_{ij}(x_i - \bar{x}) \tag{1}$$

$$S^2 = \frac{1}{N}\sum_{i=1}^{n}(x_i - \bar{x})^2 \quad (2)$$

where I represents the local spatial autocorrelation index, x_i denotes the first element of the attribute, $\bar{x}$ denotes the mean value of the attribute, W_{ij} is the spatial weighting matrix, S^2 represents the variance of the attribute, N is the number of elements.

Based on the local Moran's I index, this study used LISA maps to identify regional clustering patterns, classifying spatial associations into four categories: high-high (HH) clusters, low-low (LL) clusters, high-low (HL) spatial outliers, and low-high (LH) spatial outliers [31]. This approach facilitates the detection of statistically significant localized clusters and spatial anomalies, offering critical insights into the spatial heterogeneity of atmospheric NO_2 pollution across China's urban agglomerations [32-33]. LISA cluster analysis was further developed [34]. Specifically, the four cluster types were defined as follows: HH clusters: Regions where both the focal area and its neighboring zones exhibit elevated NO_2 concentrations. HL outliers: Areas with high NO_2 levels surrounded by regions with low concentrations. LH outliers: Regions with low NO_2 concentrations adjacent to high-pollution neighbors. LL clusters: Areas where both the focal region and its surrounding area consistently demonstrate low NO_2 levels. Non-significant clusters: Regions lacking statistically significant spatial autocorrelation. Subsequently, based on the four aggregation

types, cities were categorized and analyzed according to their stability over five years, distinguishing between those that consistently maintained a specific type of aggregation, those that changed once, and those that have undergone multiple transitions.

(3) The Random Forest model: The RF model is an ensemble learning algorithm [35], proposed by Breiman and Cutler in 2001, with a decision tree at its base learners. During pre-diction, the RF algorithm utilizes the Bootstrap resampling method to draw samples from the original dataset. By constructing multiple decision trees through iterative sampling with replacement, the RF algorithm aggregates predictions from each tree and determines the final output via majority voting, ensuring robust generalization through ensemble learning [36]. Compared to the traditional linear model, the RF model effectively captures the complex interaction between various variables, offers fast training speed, and does not require a predefined functional form [37]. Additionally, the RF model has a simple structure with fewer parameters to adjust, making it well-suited for multidimensional and multi-factor prediction while delivering highly accurate prediction results [38]. The advantages of the RF model can be summarized as follows: Accuracy-computation balance: It enhances model accuracy without significantly increasing computational complexity compared to conventional algorithms.

This study employed the PSO algorithm to optimize the hyperparameters of the RF model, facilitating efficient exploration of the hyperparameter space to identify optimal configurations for enhanced predictive performance. The RF model was trained using seasonal average tropospheric NO_2 column concentrations (2019–2023) and 15 explanatory variables, including atmospheric pressure (Pressure), Leaf Area Index (Lai), dew point temperature (Dew), aerosol optical depth (AOD), wind speed (WS), Month (Month), evapotranspiration (ET), relative humidity (RH), precipitation (Pre), fire activity (Fire), land surface temperature (LST), air temperature (Temp), gross primary productivity (GPP), year (Year), and snow cover (Snow).

(4) Shapley Additive Explanations: SHAP is an additive explanation model that evaluates the impact of in-put variables on model predictions [39], SHAP quantifies the relative im-portance of input variables by assessing the average variation in model outputs due to changes in those variables [40], This is achieved through scatter plots and SHAP value distributions, which visualize variable contributions, model performance, and any biases in the estimates [41]. Applying SHAP values to interpret the optimized RF model provides deeper insights into the relative contributions of individual factors during training [42]. Assuming that the j-th predictor of the i-th target variable is x_{ij}, the model prediction for the i-th target variable is y_i, and the average predicted value of all target variables is y_{base}, the SHAP value follows the formula [43]:

$$y_i = y_{\text{base}} + f(x_{i1}) + f(x_{i2}) + f(x_{i3}) + \ldots + f(x_{iy}) \quad (3)$$

where $F(x_{ij})$ represents the SHAP value of the j-th predictor variable for the i-th target variable, indicating the marginal contribution of this predictor to the model's prediction of the target variable. In this study, the target variable was the NO_2 column concentration, with 15 explanatory variables as predictors. Absolute SHAP values measure the magnitude of influence exerted by each predictor on the model's output, enabling variable importance ranking. A higher absolute SHAP value indicates a greater impact of the corresponding predictor on NO_2 column concentration variability.

2.3 Technical ideas

This study consists of three main components: First, using seasonal and annual time windows, statistical models such as analysis of variance (ANOVA) and Tamhane's T2 post-hoc tests were applied to analyze the temporal evolution patterns of NO_2 column concentrations across 346 major Chinese cities from 2019 to 2023. Second, local Moran's I and LISA clustering techniques were employed to identify high- and low-concentration agglomerations and spatial autocorrelation patterns. Third, these results were validated against nationwide NO_2 distribution maps derived from Sentinel-5P L3 products. Seasonal-averaged NO_2 concentrations (2019–2023) were used as the dependent variable, while 15 natural driving factors, including meteorological,

vegetation, and anthropogenic indices, served as predictors. The model was optimized using PSO, resulting in the following hyperparameters: n_estimators=4953, max_depth=18, min_samples_split=4, min_ samples_leaf=1. This study identified key predictors of air pollution by integrating the built-in feature importance ranking of the RF model with SHAP interpretation algorithms. The top five influential factors were selected to generate partial dependence plots (PDPs), illustrating their nonlinear relationships with the dependent variable (NO_2 concentrations). Analyzing these relationships provides a basis for formulating effective urban air pollution prevention and control strategies in China.

Figure 2. Technology Roadmap.

This study used NO_2 column concentrations from 346 cities as the target variable. Based on existing research by domestic and international scholars on natural factors influencing atmospheric pollutant levels, 15 predictors were selected, including Pressure, Lai, ET, and others, to train the RF model. Predictor importance was ranked using the SHAP im-portance metric, quantified by the mean absolute SHAP value for each factor. Key drivers were subsequently identified, and their marginal effects on NO_2 column concentrations were analyzed through SHAP value decomposition, revealing nonlinear relationships and threshold behaviors in pollution dynamics.

3. Results

3.1. Temporal distribution characteristics of NO_2 concentration

This study examined NO_2 concentrations across 346 major Chinese cities from 2019 to 2023. Raster datasets were homogenized to compute annual and seasonal averages, with seasons defined as follows: spring (March–May), summer (June–August), autumn (September–November), and winter (December–February). ANOVA was applied to assess interannual differences in NO_2 concentrations, accompanied by characteristic maps illustrating inter-annual and seasonal variation patterns. The interannual variability characteristics of tropo-spheric NO_2 vertical column densities in major Chinese cities are illustrated (Fig.3). From 2019 to 2023, the average annual NO_2 concentration exhibited a wave-shaped trend, initially increasing, then decreasing, and rising again. Between 2019 and 2021, NO_2 concentration gradually increased, with a smaller difference between 2020 and 2021 compared to 2019. The average annual concentrations were 1.28×10^{-4}mol/m^2, 1.38×10^{-4} mol/m^2, and 1.44×10^{-4} mol/m^2 for 2019, 2020, and 2021, respectively. The highest concentration over these three years occurred in 2021. In 2022, NO_2 concentration dropped significantly to 1.29×10^{-4} mol/m^2. In 2023, concentrations increased slightly to 1.32×10^{-4} mol/m^2.

Figure 3. Interannual variation characteristics of troposphere nitrogen dioxide vertical column density in major Chinese cities.

This study applied ANOVA and Tamhane's T2 post-hoc tests to examine seasonal aver-ages of tropospheric NO_2 vertical column densities across major Chinese cities from 2019 to 2023. This approach enabled a rigorous comparison of interannual and intraseasonal variability in NO_2 pollution patterns, revealing statistically significant differences ($p < 0.05$) across climatic zones and urbanization gradients. As summarized in Table 2, the F-values for spring, summer, autumn, and winter were 30.66, 16.76, 8.38, and 55.19, respectively, with all corresponding p-values < 0.05. This finding confirmed statistically significant differences in NO_2 mean concentrations across the five years for each season. Notably, spring and summer exhibited relatively lower interannual variability, whereas winter demonstrated the most pronounced variability, possibly due to intensified heating emissions and stagnant meteorological conditions during the colder months.

Table 2. Table of seasonal differences in five years.

Season	F	P
Spring	30.66	< 0.05
Summer	16.76	< 0.05
Autumn	8.38	< 0.05
Winter	55.19	< 0.05

Season represents the name of each Season, P value (< 0.05) represents the difference between each Season, and F value represents the degree of seasonal difference between each year. The greater the F value, the greater the difference; On the contrary, the smaller the difference.

The seasonal variability of tropospheric NO_2 vertical column densities in China's major cities is depicted (Fig.4). Distinct seasonal patterns emerged, with NO_2 concentrations following a decreasing order: winter > autumn >

spring > summer. During 2019–2023, NO_2 demonstrated significant seasonal variations, with peak concentrations observed in winter, lowest levels in summer, and moderate values in autumn and spring. The marginal difference between autumn and spring contrasted sharply with the winter-summer disparity, highlighting the dominant influence of seasonal emission patterns such as meteorological stagnation and precipitation is relatively low, making it difficult for NO_2 pollutants to settle in winter. The graph indicates more anomalies in winter, primarily due to coal combustion for heating in some northern Chinese cities and increased emissions from the fireworks and firecrackers during the Spring Festival.

Figure 4. Seasonal variation characteristics of vertical column density of

nitrogen dioxide in the troposphere of major cities in China.

The points in the figure represent the annual average values of NO_2 concentrations in each city; The square represents the mean value, and the horizontal line represents the median value.

3.2. Spatial distribution characteristics of NO_2 concentration

Significant spatial disparities in NO_2 concentrations were evident across major Chinese cities (Fig. 5), following a general decreasing gradient from east to west. High NO_2 concentration zones were primarily located east of the Hu Line (Hu Huanxian Line) and north of the Yangtze River Basin. In northern China, elevated NO_2 levels were observed across the North China Plain, Central Plains Urban Agglomeration, and the Beijing-Tianjin-Hebei region. Similarly, high concentrations were detected in the eastern coastal zones, particularly in the Yangtze River Delta and the Pearl River Delta. In Northeast China, metropolitan hubs such as Harbin and Shenyang exhibited significant NO_2 accumulation. Besides, regional hotspots were identified in northern Xinjiang,

including Urumqi and Sichuan Basin. The highlands surrounding the Sichuan Basin restrict horizontal and vertical air circulation, leading to pollutant accumulation and elevated NO_2 concentrations. This effect is particularly pronounced in provincial capitals such as Chengdu, where dense anthropogenic activities exacerbate pollution levels.

Low NO_2 concentration areas were primarily located south of the Yangtze River and west of the Hu Line, encompassing regions such as northwest China (including southern Xinjiang, Ningxia, and Qinghai), southwest China (including Tibet, Yunnan, and Guizhou), southeast China (such as Guangdong and Fujian), and northeast China (including Heilongjiang and eastern Inner Mongolia). Eastern urban areas, serving as China's economic hubs, are characterized by high population density, frequent transportation, and intensive industrial activities. The spatial distribution of NO_2 concentration across China exhibits a distinct east-west gradient, with notably higher concentrations in the east and lower levels in the west.

Figure 5. Spatial distribution of annual mean tropospheric nitrogen dioxide vertical column concentrations in major cities in China.

A local spatial autocorrelation analysis model was applied to generate LISA cluster maps, illustrating the spatial heterogeneity of air pollution severity within the study area and its surrounding regions. Fig. 6 depicts the changes in

the number of cities across various cluster types during the five years. The number of cities classified as HH and LL clusters remained relatively stable over the five-year study period. In contrast, LH clusters exhibited moderate interannual fluctuations, with annual counts of 2, 1, 3, 5, and 2 cities, respectively. This spatial pattern is influenced by Yulin's unique geographic context. Although local NO_2 concentrations were relatively low, the city borders Shanxi Province to the east, a heavily industrialized region with elevated NO_2 levels, and Yan'an City to the south. The persistent LH classification reflects the combined effects of cross-regional pollutant transport and localized atmospheric stagnation. HL clusters appeared only in 2019, with Harbin City in Heilongjiang Province as the sole representative. As a provincial capital with a large population and high traffic volume, Harbin's NO_2 emissions are primarily driven by vehicle exhaust. Furthermore, industrial zones concentrated in Harbin contributed to significantly higher NO_2 concentrations and HL cluster status compared to its neighboring cities. This spatial disparity underscores the compounded impact of anthropogenic activity, such as industrial agglomeration and urban mobility patterns, on local air quality dynamics.

Figure 6. Changes in the number of cities of each agglomeration type in five years.

Based on the cluster types, cities were categorized into three groups: cities that consistently maintained specific cluster types across the five years, those

that transitioned once, and those that transitioned multiple times. A visual analysis of atmospheric pollutants in China's major cities revealed pronounced spatial clustering of annual average NO_2 concentrations (Fig. 7).

Figure 7. Local spatial autocorrelation analysis of vertical column concentration of tropospheric nitrogen dioxide in major cities of China.

Over the five years, 54 cities consistently maintained HH clusters, primarily concentrated in the Yellow-Huai-Hai Plain, the Yangtze River Delta, and industrial hubs such as Shenyang, Benxi, and Liaoyang in Liaoning Province. These regions, particularly northeast China's traditional industrial base centered in Shenyang, exhibited persistently high NO_2 concentrations due to intensive industrial activities. In contrast, 74 cities remained LL clusters, characterized by lower industrialization levels, moderate urbanization, and geographic isolation from high-emission zones, minimizing cross-regional pollution influence. Additionally, 34 cities transitioned once in cluster classification, including Qinhuangdao, Chengde, Heihe, and Luoyang. Among these, 20 cities, such as Nanping, Meizhou, and Daqing, demonstrated relatively stable clustering patterns for 3–4 years, while 14 cities, including Ma'anshan, Jieyang, and Ya'an, exhibited non-significant clustering for 1–2 years but maintained a single cluster type in other years. A total of 12 cities, such as Fuzhou, Dandong, and Kizilsu Kirghiz Autonomous Prefecture, underwent multiple transitions in cluster classification. Notably, Xuancheng,

Bayannur, Zhaotong, and four prefecture-level cities in Xinjiang, located near provincial capitals, and Zhongshan city near the Pearl River Delta, experienced unstable clustering due to significant influence from adjacent high-emission zones. Coastal cities such as Fuzhou, Yancheng, Dandong, Yantai, and Yangzhou, which are situated along the Huaihe River's waterway, exhibited heightened variability in cluster types. This variability was attributed to unstable meteorological conditions, including fluctuations in atmospheric pressure, temperature, humidity, and WS. The observed spatial-temporal heterogeneity highlights the combined effects of industrial agglomeration, meteorological volatility, and cross-regional pollutant transport in shaping NO_2 distribution patterns across China's urban landscapes.

3.3. *Analysis of factors influencing tropospheric NO_2 column concentration in major Chinese cities*

Using the processed seasonal average concentrations of NO_2 from 2019 to 2023, the following explanatory variables were selected: Pressure, Lai, Dew, AOD, WS, Month, ET, RH, Pre, Fire, LST, Temp, GPP, Year, and Snow. These variables formed the basis for constructing an optimized RF model to analyze the spatiotemporal drivers of NO_2 variability. Predictor importance was quantified using mean SHAP values, with the top five influential factors selected to generate PDPs. The RF model, combined with SHAP interpretation algorithms, facilitated an analysis of key drivers and used PDPs to isolate the

marginal relationships between these factors and NO_2 column concentrations while controlling for other variables. Results demonstrated strong agreement between modeled and observed values, with the training and validation groups exhibiting slopes of 0.86 and 0.74 and coefficient of determination (R^2) values of 0.92 and 0.76, respectively. Scatterplots revealed a close alignment of data points along the 1:1 regression line (Fig. 8), confirming the high predictive accuracy and robustness of the optimized RF model.

Figure 8. Performance of the Random Forest (RF) model for nitrogen dioxide concentration.

(a): modeling, (b): validation

Fig. 9 illustrates the overall importance of each predictor variable, with the y-axis representing ranked variable importance and the x-axis indicating mean SHAP values. Variables were ranked from top to bottom as follows: Pressure, Lai, ET, Dew, AOD, WS, Month, RH, Pre, LST, Fire, Temp, Year, GPP, Snow. The analysis demonstrated that Pressure exerted the strongest influence on NO_2 column concentrations, followed by Lai, ET, Dew, and AOD. Based on this ranking, the top five factors, including Pressure, Lai, ET, Dew, and AOD, were identified as key drivers for in-depth analysis, while the remaining variables contributed minimally to the model's explanatory power (Fig. 9).

Figure 9. Importance ranking of the affecting factors based on RF-SHAP model.

The study revealed a nonlinear relationship between these key drivers and NO_2 concentrations by applying PDPs to analyze the top five most influential factors. Pressure exhibited the highest importance among all predictors. As Pressure increased, NO_2 concentration exhibited an overall upward trend (Fig. 10a). The relationship between Pressure and NO_2 concentrations followed a wave-shaped pattern, rapidly escalating NO_2 response magnitude when Pressure reached approximately 95,000 Pa. A pronounced positive correlation was observed in high-Pressure regions (> 95,000 Pa), indicating that elevated atmospheric Pressure consistently coincided with increased NO_2 levels. The response of AOD to NO_2 concentration can be divided into two stages (Fig. 10b). In the low AOD range, the sensitivity of AOD to NO_2 concentration increases rapidly. When AOD reached approximately 330, the response gradually stabilized. NO_2 participates in photochemical reactions in the atmosphere with other compounds, forming secondary aerosols. These secondary aerosols increase PM concentration, thereby elevating AOD values. The influence of Lai on NO_2 concentration exhibited a nonlinear trend, initially declining and then stabilizing (Fig. 10c). Lower Lai values correspond to higher NO_2 concentrations, while higher Lai values correspond to low-er concentrations. ET exhibited a negative correlation with NO_2 concentration (Fig. 10d). As ET increases, NO_2 concentration decreases, following an overall nonlinear trend of an initial sharp decline followed by gradual stabilization. Additionally, Dew demonstrated a negative correlation with NO_2 concentration, characterized by

a brief increase followed by a continuous decrease, primarily divided into two phases (Fig. 10e). Below approximately 263 K (low Dew range), Dew and NO_2 concentration exhibited a positive correlation. When Dew reaches approximately 263 K (high Dew range), the relationship shifts from positive to negative correlation, with NO_2 concentration gradually decreasing as Dew increases.

Figure 10. Influence intensity of Pressure, AOD, Lai, ET, Dew

on nitrogen dioxide concentration.

Pressure: atmospheric pressure; AOD: aerosol optical depth; Lai: Leaf Area Index; ET: evapotranspiration; Dew: dew point temperature. The blue line represents the trend of the impact of key influencing factors on NO_2 concentration variation. A SHAP value > 0 indicates a positive contribution, confirming that the factor's influence on NO_2 concentration change increases as the SHAP value rises. Conversely, a SHAP value < 0 indicates a negative contribution, where a decreasing SHAP value corresponds to a stronger negative impact on NO_2 concentration changes.

4. Discussion

A previous study applied the geographically weighted regression model to examine the concentration of $PM_{2.5}$ determinants and explore atmospheric pollutant variations [44]. Research has employed principal component analysis and concluded that effective pollution control requires coordinated management of major pollutants, including PM_{10}, $PM_{2.5}$, and O_3 [45]. The RF

model offers several advantages, such as low parameter sensitivity to reduce overfitting issues [46], strong robustness against missing values and outliers, and the ability to enhance computational efficiency through optimized variable selection [47]. This study employed an optimized RF model to identify key factors influencing NO_2 concentrations, providing theoretical foundations and data-driven insights for atmospheric pollution control.

This study analyzed correlation coefficients between variables to gain deeper insight in-to the factors and the mechanisms influencing NO_2 concentrations (Fig. 11), the sample points comprised monthly average data from 346 cities spanning 2019 to 2023. Analysis of 15 variables determined the correlation coefficients between variable pairs. The results indicated strong correlations (coefficients: 0.5–1.0) between Lai and ET, GPP, Dew, Pre, LST, and Temp, whereas weak correlations (coefficients: 0–0.5) were observed between AOD, Year, and other factors. These findings suggest that the combined effects of vegetation and atmospheric processes primarily influence urban air pollution. Furthermore, the results vali-date the characteristic seasonal pattern of NO_2 pollution in China, which is higher in winter and lower in summer and exhibits strong cyclical fluctuations. The spatial distribution features of NO_2 concentration hotspots and cold spots were also confirmed.

Figure 11. Thermodynamic map of correlation of natural factors affecting NO_2 concentration.

AOD (aerosol optical depth), Dew (dew point temperature), ET (evapotranspiration), Fire (fire activity), GPP (gross primary productivity), Lai

(Leaf Area Index), LST (land surface temperature), Month(month), Pre (precipitation), Pressure (atmospheric pressure), RH (relative humidity), Snow (snow cover), TEMP (air temperature), WS (wind speed), Year (year).

The PSO algorithm was applied to optimize the RF model's hyperparameters and evaluate its robustness. Model performance was validated by calculating the R^2 using a linear regression model, indicating reasonable fitting accuracy (Fig. 8). The built-in feature im-portance ranking function of the RF model was employed to prioritize 15 factors, including Pressure, Lai, Dew, ET, RH, AOD, Fire, GPP, Temp, Year, Month, Pre, LST, WS, and Snow, across the studied cities. Combined with the SHAP interpretability algorithm (Fig. 9). The analysis identified Pressure, AOD, Lai, ET, and Dew as the top five factors influencing NO_2 concentration variations, emphasizing the significant roles of atmospheric pressure and vegetation in urban NO_2 pollutant dynamics. This finding contrasts with Wu et al who re-ported Temp and Pre as dominant drivers of NO_2 variability in the Shaanxi-Gansu-Ningxia region [48]. The discrepancy possibly arises from the region's unique topo-graphic and climatic characteristics, highlighting the spatial heterogeneity of NO_2 pollution mechanisms.

NO_2 concentrations exhibited strong seasonal variability, peaking in winter and declining in summer (Fig. 4). This pattern results from elevated atmospheric pressure and lower winter temperatures, which reduce the

planetary boundary layer height and hinder pollutant dispersion [49]. Stable air masses further contribute to NO_2 accumulation, increasing concentrations [50]. Centralized coal-based heating in northern China during winter also intensifies NO_2 emissions [51]. Conversely, summer conditions enhance pollutant dispersion. As depicted in Fig. 5, interannual trends indicate a gradual increase in annual average NO_2 concentrations from 2019 to 2023, peaking in 2021. This pattern aligns with Zhang et al, who reported similar NO_2 temporal variability in China, possibly influenced by prolonged high-Pressure systems during an extreme cold wave in December 2021 [52]. Notably, 2022 recorded the lowest annual NO_2 concentration within five years, followed by a rebound in 2023. Meteorological reports attribute the decline in 2022 to weaker cold air activity compared to previous years, alongside stringent pollution control measures during the 24th Winter Olympic Games in Beijing [53], collectively reducing emissions. In contrast, stronger cold air processes in 2023 may explain the renewed increase in NO_2 levels.

The model identified Lai, ET, and Dew as three of the top five influencing factors, all vegetation-related variables. Correlation analysis underscores vegetation as a key regulator of NO_2 concentrations. Lai, defined as the total one-sided green leaf area per unit ground surface [54], enhances photosynthetic efficiency, reducing atmospheric NO_2 through direct uptake and indirect effects on precipitation-driven deposition [55]. Seasonally, higher summer Lai

values correspond to lower NO_2 due to increased vegetation absorption and ET-driven rainfall. Spatially, southern China's dense vegetation exhibits stronger NO_2 removal capacity compared to northern regions, underscoring the role of urban greening and vegetation coverage in shaping NO_2 spatiotemporal patterns [56].

This study explored the spatiotemporal distribution of NO_2 across 346 Chinese cities and its driving mechanisms. The findings offer a theoretical and data-driven basis for enhancing air pollution mitigation strategies and environmental policies, providing actionable insights for prioritizing regional pollution control efforts.

5. Conclusions

This study analyzed the interannual and seasonal spatiotemporal distribution characteristics of NO_2 concentrations in 346 major Chinese cities from 2019 to 2023, considering the impact of the COVID-19 pandemic, using satellite remote sensing data, statistical methods, and local spatial autocorrelation analysis. The key findings are summarized as follows:

(1) NO_2 concentrations exhibited significant temporal heterogeneity across the five years. Annual trends demonstrated an initial rise, followed by a decline and rebound. Seasonally, concentration remained consistently higher in winter and lower in summer.

(2) NO_2 concentrations across 346 major Chinese cities displayed notable spatial clustering. High-concentration hotspots were mainly distributed in urban areas of the North China Plain, Yangtze River Delta, and northeastern China. In contrast, low-concentration zones were concentrated in northwestern, southwestern, southern coastal, and Inner Mongolia regions. The disparity between eastern and western China arises from denser populations and intensified socioeconomic activities in the east and central areas, leading to higher NO_2 emissions. Additionally, monsoon climates in eastern China contribute to increased summer Pre, which dilutes NO_2 levels, while centralized coal heating in northern regions during winters exacerbates NO_2 accumulation, resulting in pronounced seasonal contrasts.

An RF model incorporating 15 influencing factors was optimized through iterative hyperparameter tuning, achieving high predictive accuracy with training and validation datasets closely aligned along the 1:1 fit line. Feature importance ranking using the SHAP algorithm identified Pressure, AOD, Lai, ET, and Dew as the top five drivers of NO_2 variability. SHAP analysis revealed the following patterns: Pressure exhibited a positive correlation with NO_2 concentrations, following a wave-shaped upward trend. AOD transitioned from negative to positive effects on NO_2 concentrations at thresholds of approximately 330. Lai and ET displayed negative correlations, with NO_2 concentrations decreasing initially and stabilizing as these factors increased.

Dew exerted a greater influence on NO_2 concentrations near 263 K. Three different temperature measurements, dew point temperature, surface temperature, air temperature. They themselves have a strong correlation. This shows that NO_2 concentration is strongly influenced by temperature.

Author Contributions:

Conceptualization: Feifei Cheng.Qing Sun and Heming Yang.

Methodology: Feifei Cheng. Qing Sun. Jiaqi Zhang and Jiahe Liu.

formal analysis: Feifei Cheng and Qing Sun.

data curation: Feifei Cheng and Qing Sun.

writing—original draft preparation: Feifei Cheng and Qing Sun.

writing—review and editing: Feifei Cheng and Qing Sun.

visualization: Feifei Cheng. Qing Sun. Jiaqi Zhang. Jiahe Liu. Wenwen Lü. Fapeng Shen.

Supervision: Heming Yang.

funding acquisition: Heming Yang.

Acknowledgments: This research was funded by Jilin Provincial Department of science and technology, Approval No.: YDZJ202401520ZYTS.

References

1. Wu, Z, C.; Tian, Y, P.; Li, M, Z.; Wang, B.; Quan, Y.; Liu, J, Y. Prediction of air pollutant concentrations based on the long short-term memory neural network. *J. Hazard. Mater.* 2024, 465, 133099. https://doi.org/10.1016/j.jhazmat.2023.133099
2. Accarino, G.; Lorenzetti, S.; Aloisio, G. Assessing correlations between short-term exposure to atmospheric pollutants and COVID-19 spread in all Italian territorial areas. *Env. pollution*. 2021, 268, 115714. https://doi.org/10.1016/j.envpol.2020.115714

3. Rahaman, S.; Tu, X.; Ahmad, K.; Qadeer, A. A real-time assessment of hazardous atmospheric pollutants across cities in China and India. *J. Hazard. Mater*. 2024, 479, 135711. https://doi.org/10.1016/j.jhazmat.2024.135711
4. Ojeda-Castillo, V.; Murillo-Tovar, M.A.; Hernández-Mena, L.; Saldarriaga-Noreña, H.; Vargas-Amado, M.E.; Herrera-López, E.J.; Díaz, J. Tropospheric NO_2: Anthropogenic Influence, Global Trends, Satellite Data, and Machine Learning Application. *Remote Sens*. 2025, 17, 49. https://doi.org/10.3390/rs17010049
5. Huangfu, P, J.; Atkinson, R. Long-term exposure to NO_2 and O_3 and all-cause and respiratory mortality: a systematic review and meta-analysis. *Environ Int.* 2020, 144, 105998. https://doi.org/10.1016/j.envint.2020.105998
6. Rani, B.; Singh, U.; Chuhan, A. K.; Sharma, D.; Maheshwari, R. Photochemical Smog Pollution and Its Mitigation Measures. J *Adv Res*. 2011, 2, 28-33. https://www.sciensage.info/index.php/JASR/article/view/56
7. Lim, Chul-Hee.; Ryu, J.; Choi, Y.; Seong, W, J. Understanding global $PM_{2.5}$ concentrations and their drivers in recent decades (1998–2016). *Environ Int*. 2020, 144, 106011. https://doi.org/10.1016/j.envint.2020.106011
8. Xian, Y.; Wang, L, L.; Shan, L. NO_2 Pollution Characteristics in Autumn and Winter of Jiangsu Province in 2020-2021. *Environ. Monit. Forewarning.* 2021, 13, 47-53. https://doi.org/10.3969/j.issn.1674-6732.2024.06.014
9. Michelle, S.W. Kusters.; Laura, G.; Sami, P.; Henning T.; Ryan L.M.; Mònica G. Exposure to residential air pollution and the development of functional connectivity of brain networks throughout adolescence. *Environ Int.* 2025, 196, 109245. https://doi.org/10.1016/j.envint.2024.109245
10. Coker, E.S.; Cavalli, L.; Fabrizi, E.; Guastella, G.; Lippo, E.; Parisi, M.L.; Pontarollo, N.; Rizzati, M.; Varacca, A.; Vergalli, S. The effects of air pollution on COVID-19 related mortality in Northern Italy. *Environ. Re-sour. Econ.* 2020, 76, 611–634. https://doi.org/10.1007/s10640-020-00486-1
11. Diener, A.; Mudu, P. How can vegetation protect us from air pollution? A critical review on green spaces' mitigation abilities for air-borne particles from a public health perspective-with implications for urban planning. *STOTEN*. 2021, 796, 148605. https://doi.org/10.1016/j.scitotenv.2021.148605
12. Ma, Y, D, Y.; Su, B, B.; Li, D, K.; Cui, F, P.; Tang, L, X.; Wang, J, N.; Tian, Y, H.; Zheng, X, Y. Air pollution, genetic susceptibility, and the risk of atrial fibrillation: A large prospective cohort study. *Proc Natl Acad Sci USA*. 2023, 120, e2302708120. https://doi.org/10.1073/pnas.2302708120
13. Li, X, Y.; Lin, X, Q.; Wang, D. Spatiotemporal evolution characteristics and influencing factors of urban air quality in China. *J. Xi'an Univ. Technol*. 2022, 38, 487-499. https://doi.org/10.19322/j.cnki.issn.1006-4710.2022.04.004
14. Müller, I.; Erbertseder, T.; Taubenböck, H. Tropospheric NO_2: Explorative analyses of spatial variability and impact factors. *Remote Sens. Environ*. 2022, 270, 112839. https://doi.org/10.1016/j.rse.2021.112839
15. Han, J, D.; Liu, H.; Zhu, H, S. Kill Two Birds with One Stone: A Multi-View Multi-Adversarial Learning Approach for Joint Air Quality and Weather Prediction. *IEEE Trans. Knowl. Data Eng.* 2023, 35, 11515-11528. https://doi.org/10.1109/TKDE.2023.3236423
16. Khajavi, H.; Rastgoo, A. Predicting the carbon dioxide emission caused by road transport using a Random Forest (RF) model combined by Meta-Heuristic Algorithms. *SCS.* 2023, 93, 104503. https://doi.org/10.1016/j.scs.2023.104503
17. Forster, P.M.; Smith, C.; Walsh, T.; Lamb, W.F., et al. Indicators of Global Climate Change 2022: annual update of large-scale indicators of the state of the climate system and human influence. *Earth Syst. Sci. Data.* 2023, 15, 2295–2327. https://doi.org/10.5194/essd-15-2295-2023
18. Gupta, K, V.; Gupta, A.; Kumar, D.; Sardana, A. Prediction of COVID-19 Con-firmed, Death, and Cured Cases in India Using Random Forest Model. *Big Data Min. Anal.* 2021, 4, 116-123. https://doi.org/10.26599/BDMA.2020.9020016

19. Wang, T.; Chen, M, P.; Zhou, M, P.; Li, J.; Wu, S, J.; Luo, M.; Xia, Z, H. Pollution Characteristics and Influence factors of air pollutants in Wuxi City. *Environ. Pollut. & Control.* 2015, 37, 74-78. https://doi.org/10.15985/j.cnki.1001-3865.2015.12.015
20. Liu, Y, Y.; Li, Y.; Miao, S, G. Spatial-temporal Characteristics and Meteorological Factors Analysis of Air Pollution in Fang Shan District of Beijing. *Met&EnvSci.* 2018, 41, 62-71. https://d.wanfangdata.com.cn/periodical/hnqx201804009
21. Kong, F, B.; Cheng, D.; Zhang, Q. Spatiotemporal Changes and Influencing Factors of Dust fall in Gansu Province During the 13th Five-Year Plan Period. *Environmental Monitoring in China.* 2022, 38, 25-31. https://doi.org/10.19316/j.issn.1002-6002.2022.03.04
22. Li, J.; Chen, H.; Li, Z.; Wang, P.; Cribb, M.; Fan, X. Low-level temperature inversions and their effect on aerosol condensation nuclei concentrations under different largescale synoptic circulations. *Adv. Atmos. Sci.* 2015, 32, 898–908.
23. You, T.; Wu, R.; Huang, G.; Fan, G. Regional meteorological patterns for heavy pollution events in Beijing. *Meteorological. Res.* 2017, 31, 597–611. https://doi.org/10.1007/s13351-017-6143-1
24. Islam, A.R.M.T.; Hasanuzzaman, M.; Azad, M.A.K.; Salam, R.; Toshi, F.Z.; Khan, M.S.I.; Monirul Alam, G.M.; Ibrahim, S.M. Effect of meteorological factors on COVID-19 cases in Bangladesh. *Environ. Dev. Sustain.* 2020, 23, 9139-9162. https://doi.org/10.1007/s10668-020-01016-1
25. Yin, Z.; Wang, H.; Chen, H. Understanding severe winter haze events in the north China plain in 2014: roles of climate anomalies. *Atmos. Chem. Phys.* 2017, 17, 1–27. https://doi.org/10.5194/acp-17-1641-2017
26. Sangkham, S.; Thongtip, S.; Vongruang, P. Influence of air pollution and meteorological factors on the spread of COVID-19 in the Bangkok Metropolitan Region and air quality during the outbreak. *Environ. Res.* 2021, 197, 111104. https://doi.org/10.1016/j.envres.2021.111104
27. Chen, Z, Q. Air quality, influence factors and control countermeasure in Quanzhou, southeastern coast of China. *Journal of Earth Environment.* 2019, 10, 201-209. https://doi.org/10.7515/JEE182075
28. Li, S.; Ren, A, L.; Guo, B.; Du, Z.; Zhang, S.; Tian, M.; Wang, S. Influence of Meteorological Factors and VOCs on $PM_{2.5}$ during Severe Air Pollution Period in Shijiazhuang in Winter. 2015 2nd International Conference on Machinery, Materials Engineering, Chemical Engineering and Biotechnology. https://doi.org/10.2991/mmeceb-15.2016.116
29. Guo, L, C.; Zhang, Y.; Lin, H.; Zeng, W.; Liu, T.; Xiao, J.; Rutherford, S.; You, J.; Ma, W. The washout effects of rainfall on atmospheric particulate pollution in two Chinese cities. *Environ. Pollut.* 2016, 215, 195–202. https://doi.org/10.1016/j.envpol.2016.05.003
30. Guo, W, W.; Chen, Y, J.; Liu, G.; Song, K, S.; Tao, B, X. Analysis on the characteristics and influencing factors of air quality of urban agglomeration in the middle reaches of the Yangtze River in 2016 to 2019. *Ecology and Environment Sciences.* 2020, 29, 2034-2044. https://doi.org/10.16258/j.cnki.1674-5906.2020.10.014
31. Tsui, T.; Derumigny, A.; Peck, D.; Timmeren, A.V. Spatial clustering of waste reuse in a circular economy: A spatial autocorrelation analysis on locations of waste reuse in the Netherlands using global and local Moran's I. Frontiers in Built Environment. 2022, 8. https://doi.org/10.3389/fbuil.2022.954642
32. Anselin L. Local Indicators of Spatial Association—LISA. *Geogr. Anal.* 1995, 27, 1538-4632. https://doi.org/10.1111/j.1538-4632.1995.tb00338.x
33. Anselin L.; Sridharan S.; Gholston S. Using exploratory spatial data analysis to leverage social indicator databases: The discovery of interesting patterns. *Soc. Indic. Res.* 2007, 82, 287-309. https://link.springer.com/article/10.1007/s11205-006-9034-x
34. Littenberg, T.B.; Cornish N.J. Prototype Global Analysis of LISA Data with Multiple Source Types. *PRD.* 2023, 107, 063004. https://doi.org/10.1103/PhysRevD.107.063004
35. Gromping U. Variable importance assessment in regression: Linear regression versus random forest. *TAS.* 2009, 63, 308-319. https://doi.org/10.1198/tast.2009.08199

36. Zhang, Z, H.; Chen, N.; Zhu, B.; Tao, H, T.; Cheng, H, R. Source Analysis of Ambient $PM_{2.5}$ in Wuhan City Based on Random Forest Model. *Environ. Sci.* 2022, 43, 1151–1158. https://doi.org/10.13227/j.hjkx.202108051
37. Huang, L.; Zhu, Y, H.; Zhai, H, H.; Xue, S, H.; Zhu, T, Y.; Shao, Y.; Liu, Z, Y.; Emery, C.; Yarwood, G.; Wang, Y, J.; Fu, J.; Zhang, K.; Li, L. Recommendations on benchmarks for numerical air quality model applications in China–Part 1: $PM_{2.5}$ and chemical species. *ACP.* 2021, 21, 2725–2743. https://doi.org/10.5194/acp-21-2725-2021
38. Cao, Y.; Gong, M, Y.; Shen, F.; Ma, J, J.; Yang, G.; Lin, X, W. Estimation of $PM_{2.5}$ Concentration in China and Analysis of Influencing Factors. Journal of Atmospheric and Environmental Optics. 2023, 18, 245–257. http://gk.hfcas.ac.cn/EN/Y2023/V18/I3/245
39. Lundberg, S, M., LEE, S, I. A unified approach to interpreting model predictions. Proceedings of the 31st International Conference on Neural Information Processing Systems. *ACM.* 2017. https://doi.org/10.48550/arXiv.1705.07874
40. Tan, Z, H.; Ma, S.; Han, D.; Gao, D.; Yan, W. FY-4A cloud base height estimation method based on random forest algorithm. *Infrared Millim. Waves.* 2019, 38, 381-388. https://doi.org/10.11972/j.issn.1001-9014.2019.03.020
41. Pakdehi, M.; Ahmadisharaf, E. Azimi, P.; Yan, Z.; Keshavarz, Z.; Caballero, C.; Allen, J.G. Modeling the latent impacts of extreme floods on indoor mold spores in residential buildings: Application of machine learning algorithms. *Environ Int.* 2025, 196, 109319. https://doi.org/10.1016/j.envint.2025.109319
42. Zhang, X, S.; Nie, D, W.; Chen, Z, Z.; Wang, R, Z.; Su, J. Analysis of spatiotemporal evolution characteristics and influencing factors of carbon emissions in the construction industry in western China. *Environ. Sci.* 2024, 1-22. https://doi.org/10.13227/j.hjkx.202408170
43. Wang, S.; Peng, H.; Hu, Q.; Jiang, M. Analysis of runoff generation driving factors based on hydrological model and interpretable machine learning method. *J HYDROL-REG STUD.* 2022, 42, 101139. https://doi.org/10.1016/j.ejrh.2022.101139
44. Li, M, H.; Que, X.; Liu, J, F.; Su, S, Q.; Ding, X, T. Spatial heterogeneity analysis of $PM_{2.5}$ influencing factors in Chinese cities. *Journal of Chifeng University (Natural Science Edition)*. 2021, 37, 37-41. https://doi.org/10.3969/j.issn.1673-260X.2021.01.012
45. Bu, X, B.; Fang, Z, L.; Feng, Q.; Liao, C.; Wang, H, Z. Comprehensive Evaluation of Air Quality Based on Principal Component Analysis—Take 21 Cities in Sichuan Province as an Example. *SICHUAN ENVIRONMENT.* 2023, 42, 51-56. https://doi.org/10.14034/j.cnki.schj.2023.03.009
46. Dietterich, T, G. An experimental comparison of three methods for constructing ensembles of decision trees: bagging, boosting, and randomization. *ML*. 2000, 40, 139-157. https://doi.org/10.1023/A:1007607513941
47. Yang, X, T.; Kang, P.; Wang, A, Y.; Zang, Z, L.; Liu, L. Prediction of ozone pollution in Sichuan basin based on random forest model. *Environ. Sci.* 2024, 45, 2507-2515. https://doi.org/10.13227/j.hjkx.202304226
48. Wu, Y, R.; Wang, H.; Wang, M, J. Spatiotemporal variation and impact factors of tropospheric NO_2 column density in Shanxi-Gansu-Ningxia region based on OMI satellite data. *Journal of atmospheric and environmental and optics.* 2023, 18, 553-568. https://doi.org/10.3969/j.issn.1673-6141.2023.06.005
49. Feng, J, J.; Shen, J, F.; Liang, R, Z.; Mo, C, H. Analysis of Relationship of Meteorological Elements and PM10 in Guangzhou. *Environmental Monitoring in China*. 2009, 25, 78-82. https://api.semanticscholar.org/CorpusID:132431231
50. Wang, H.; Chen, X, Q.; Yu, Y, J.; Chen, B, B.; Sui, P. Preliminary analysis of distribution characteristics of $PM_{2.5}$, PM2.5/PM10 and their relationship with meteorological conditions in Fuzhou. *J. Trop. Meteorol.* 2014, 30, 387-391.

51. Dai, L, H. How Trade Openness Affects $PM_{2.5}$ Causal Effect Studies Based on Heating Policy Differences between the Two Sides of the Huai River. *Management Review.* 2017, 29, 237-245. http://123.57.61.11/jweb_glpl/EN/Y2017/V29/I5/237
52. Liu, Z, K.; Yuan, W, J. Diagnostic Analysis of a Cold Wave Weather Process in December 2021. *Climate Change Research Letters.* 2023, 12, 18-30. https://doi.org/10.12677/CCRL.2023.121003
53. Shi, L, J.; Suo, N.; Ma, B, J.; Tang, T.; Wang, S.; Ma, X, Y.; Ji, X, T.; Tian, Y, T.; Zhu, H.; Niu, H, Y. Evolution of $PM_{2.5}$ and O_3 pollution in Qin Huang Dao City during the period of continuous air quality improvement from 2018 to 2022. *Environ. Sci.* 2024, 10, 13227. https://doi.org/10.13227/j.hjkx.202402150
54. Zhou, W, W.; Qian, C, M.; Shu, Q, T.; Qiu, S.; Huang, J, J.; Yu, J, G.; Gao, Y, Q.; Guo, C, S. Lai Estimation of Pinus densata in Northwest Yunnan Based on UAV and Sentinel–2 Data. *Journal of Southwest Forestry University (Natural Sciences).* 2024, 44, 141–149. https://doi.org/10.11929/j.swfu.202310009
55. Wang, Y.; Teng, Z, Y.; Zhang, X, L.; Che, Y, H.; Sun, G, Y. Research progress on the effect of atmospheric NO_2 on plant growth and metabolism. *Chinese Journal of Applied Ecology.* 2019, 30, 316-324. https://doi.org/10.13287/j.1001-9332.201901.028
56. Wang, L. Analysis of Purification Effect of Landscape Vegetation on Urban Air Pollution. *ESM.* 2024, 49, 162-166. https://doi.org/10.3969/j.issn.1673-1212.2024.07.035

Supporting information captions: All additional documents have been submitted to the system.

Click here to access/download;Figure;Figure_1.tiff

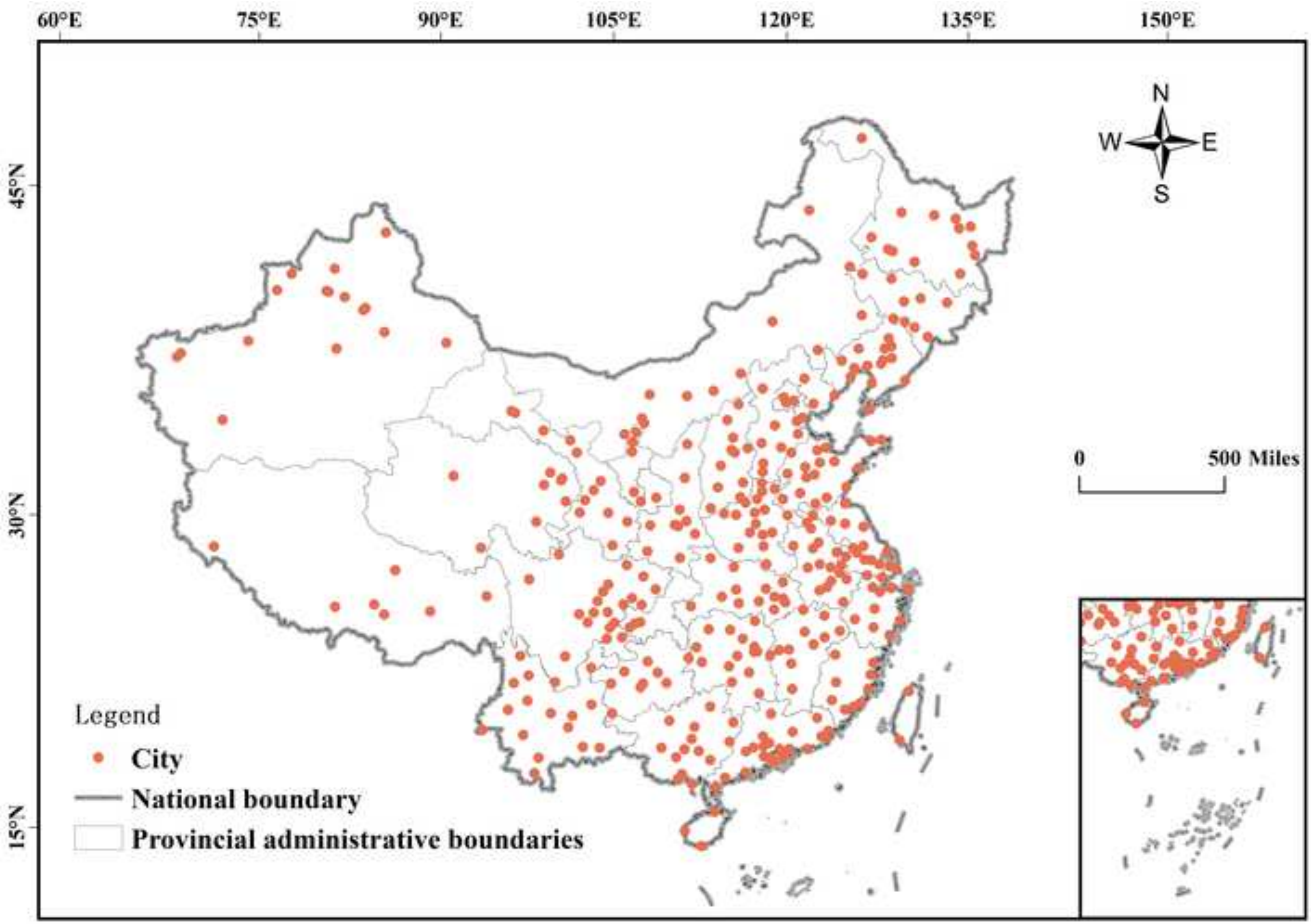

Click here to access/download;Figure;Figure_2.tiff

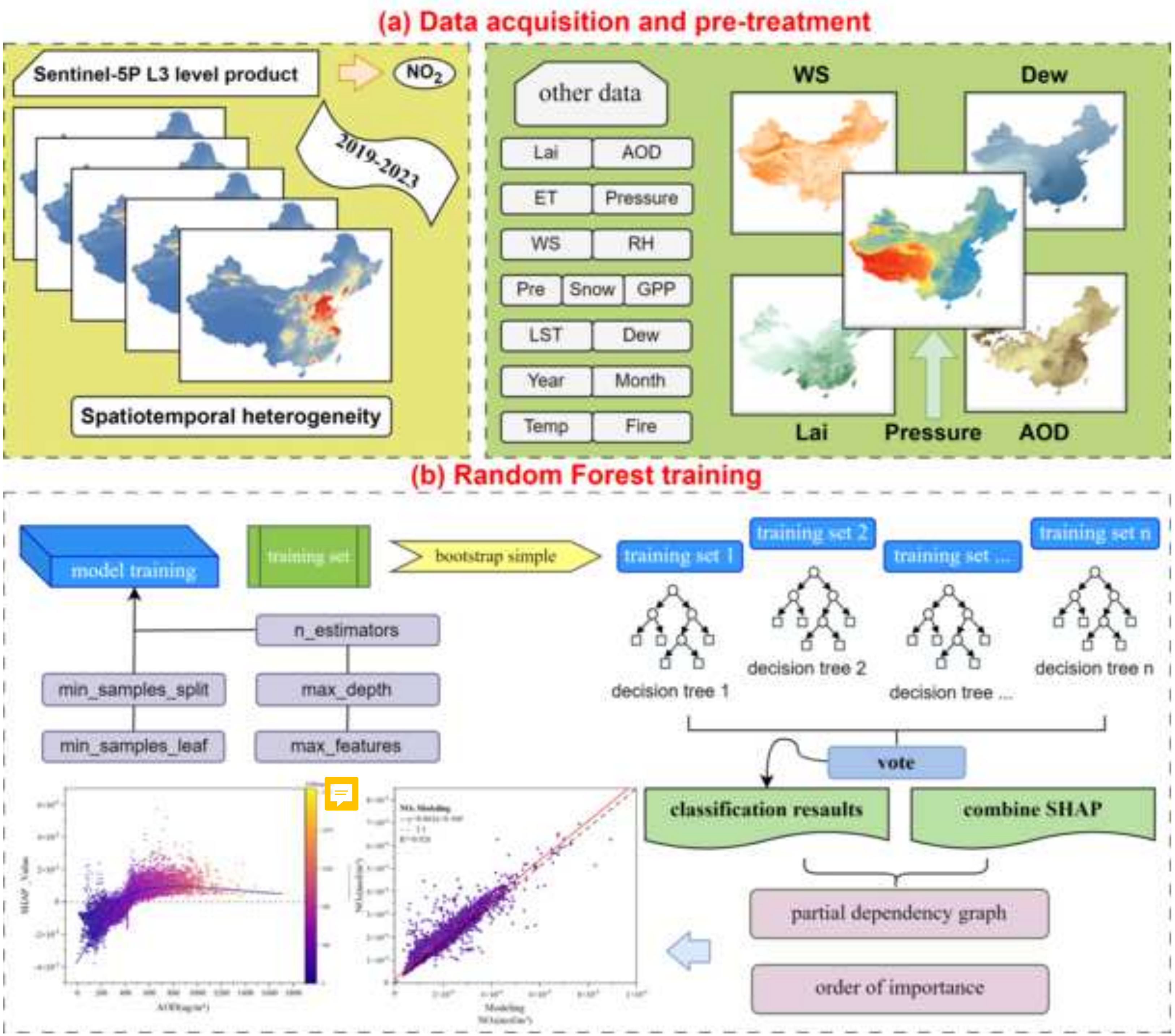

Click here to access/download;Figure;Figure_3.tiff

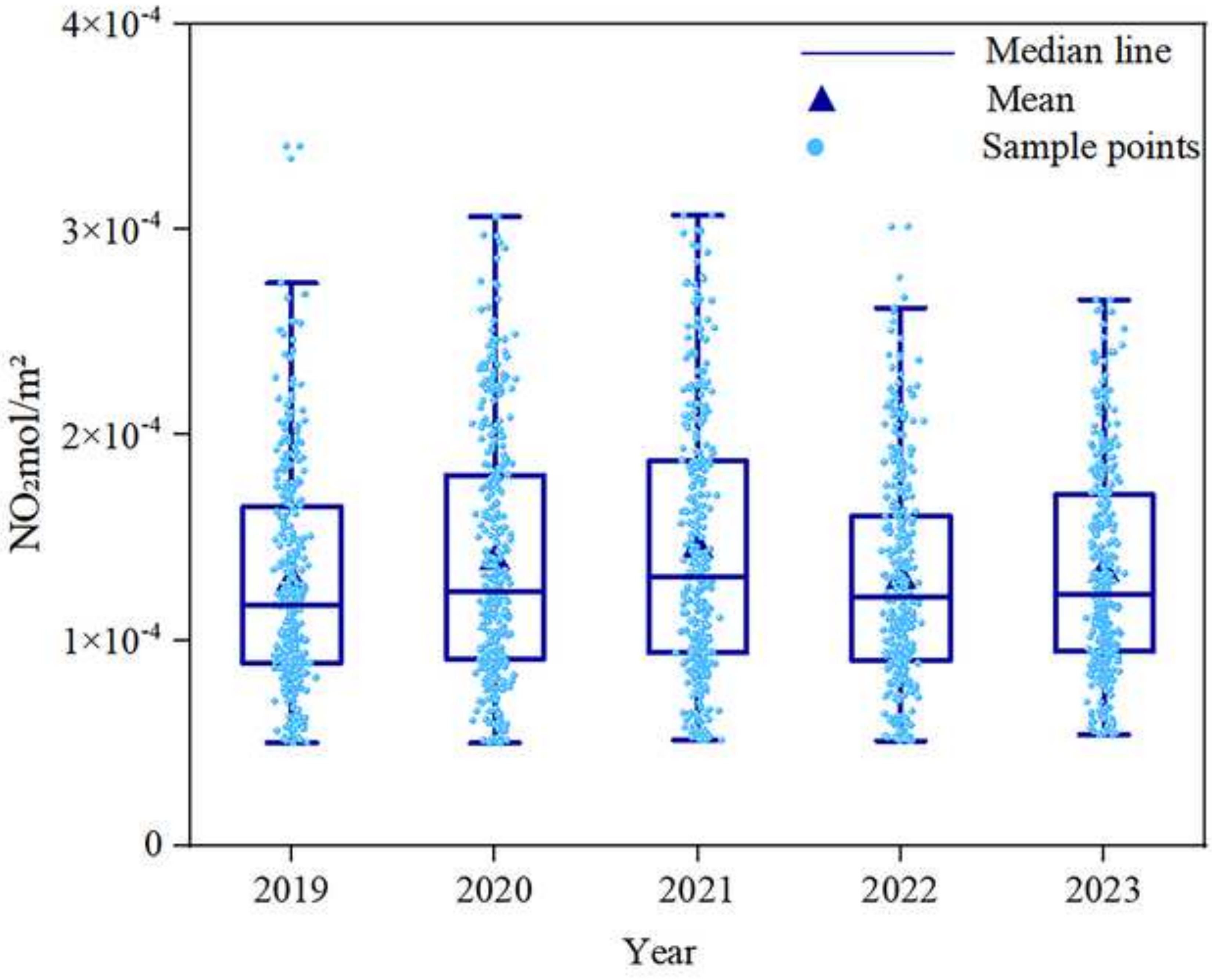

Click here to access/download;Figure;Figure_4.tiff

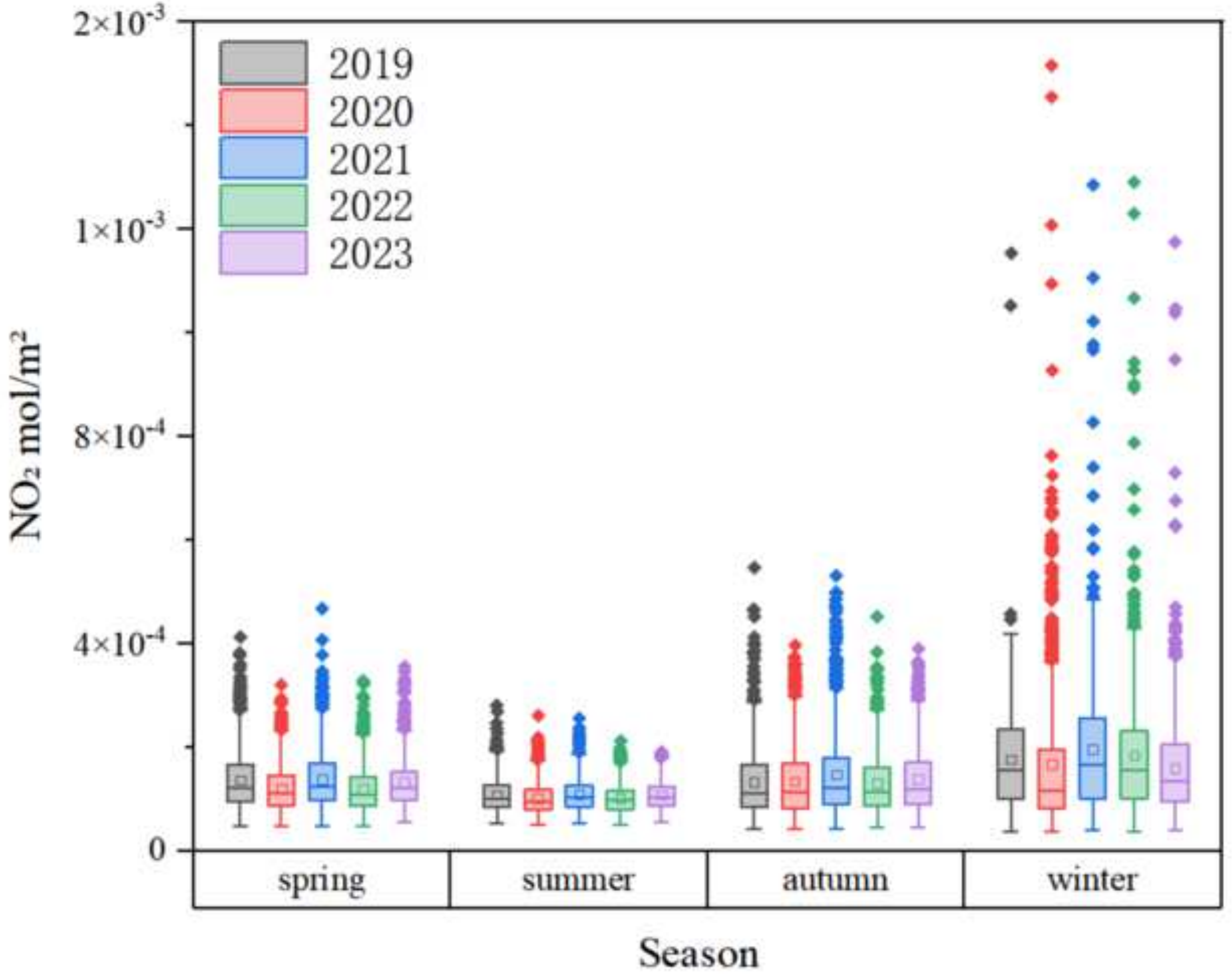

Click here to access/download;Figure;Figure_5.tiff

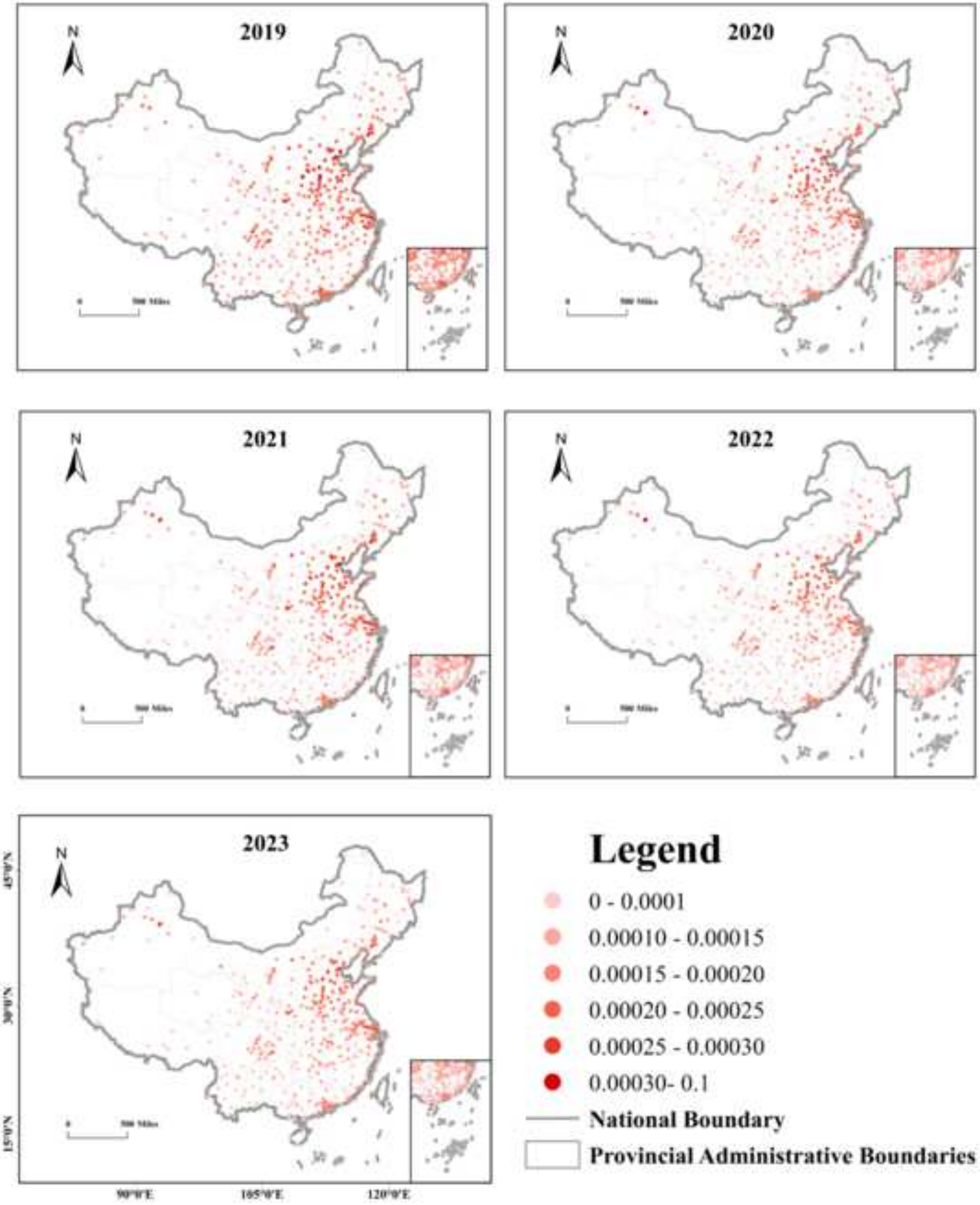

Click here to access/download;Figure;Figure_6.tiff

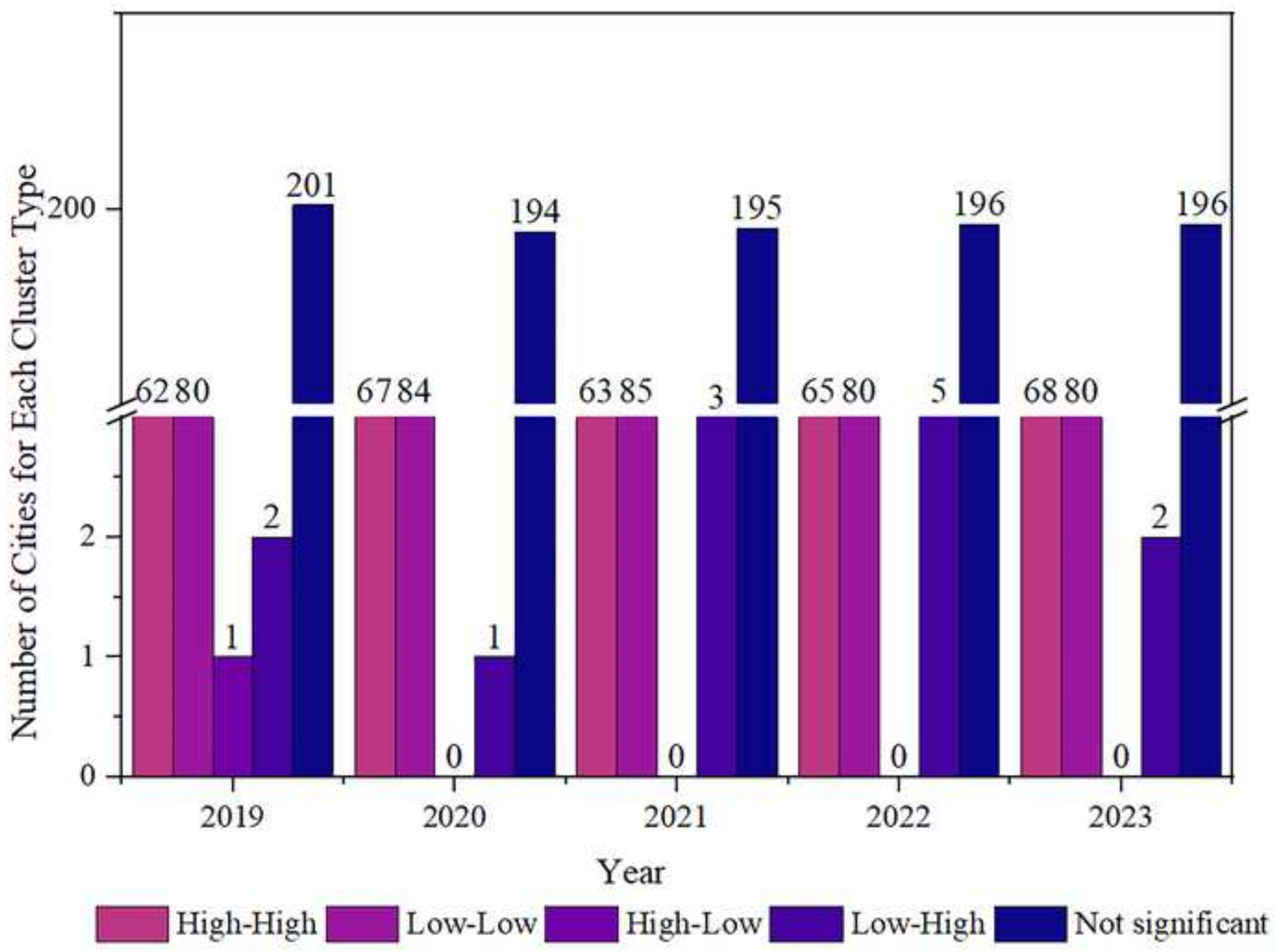

Click here to access/download;Figure;Figure_7.tiff

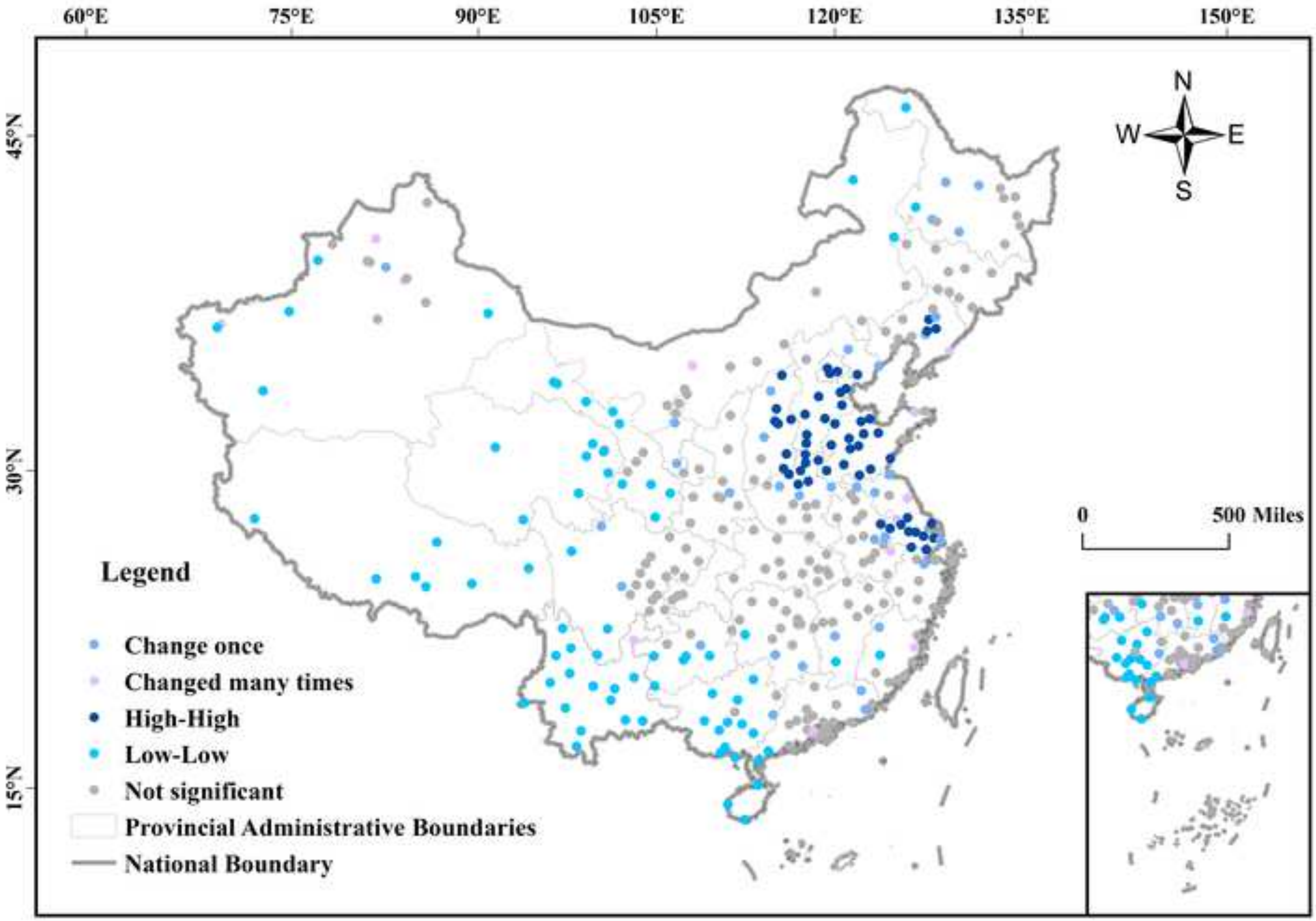

Click here to access/download;Figure;Figure_8.tiff

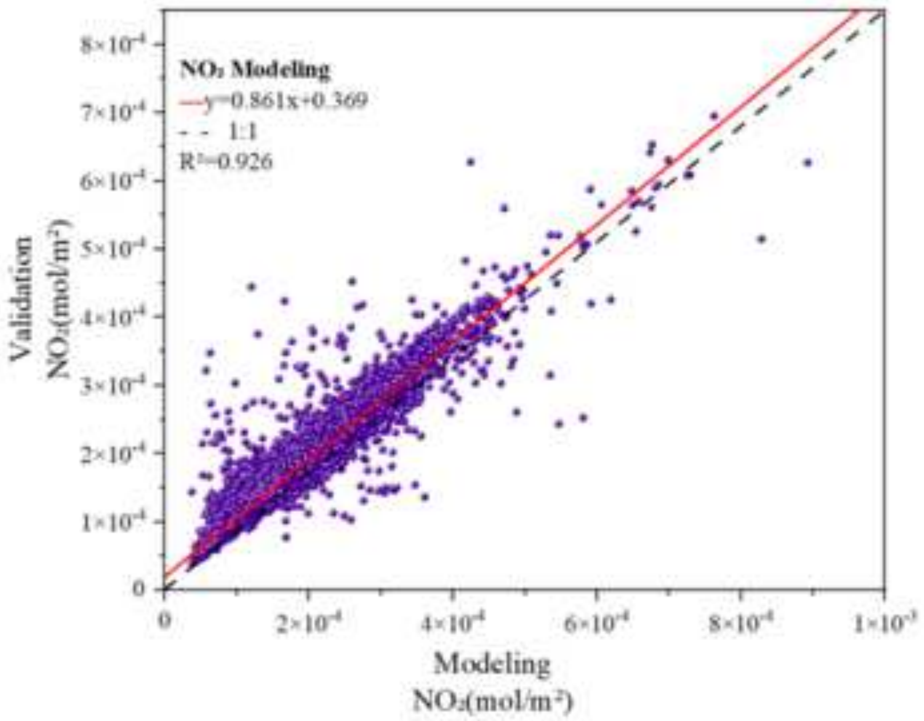

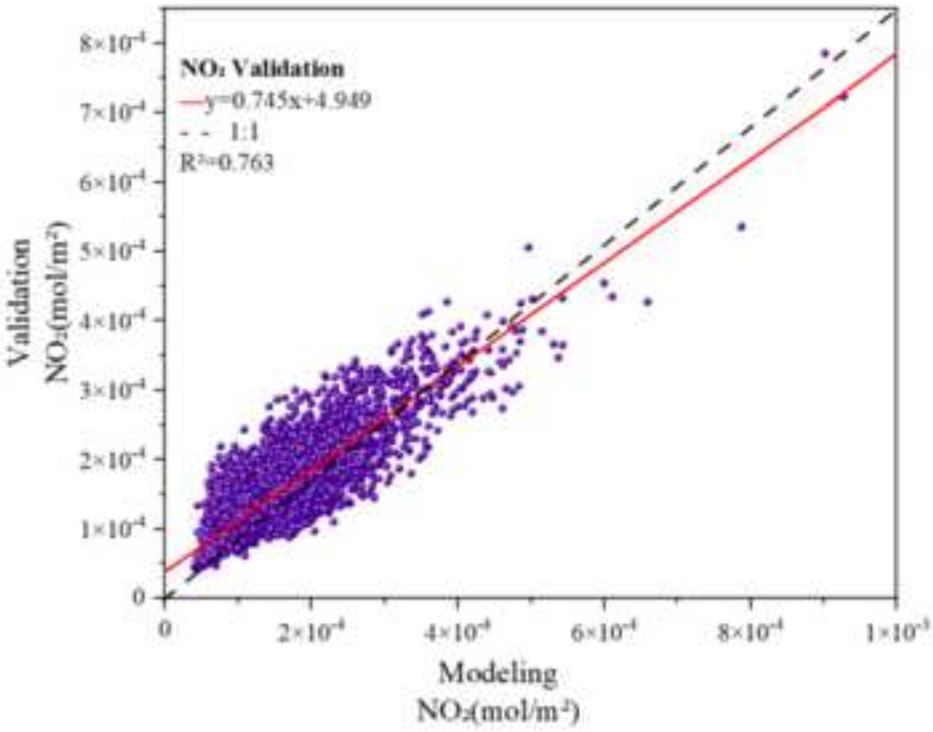

Click here to access/download;Figure;Figure_9.tiff

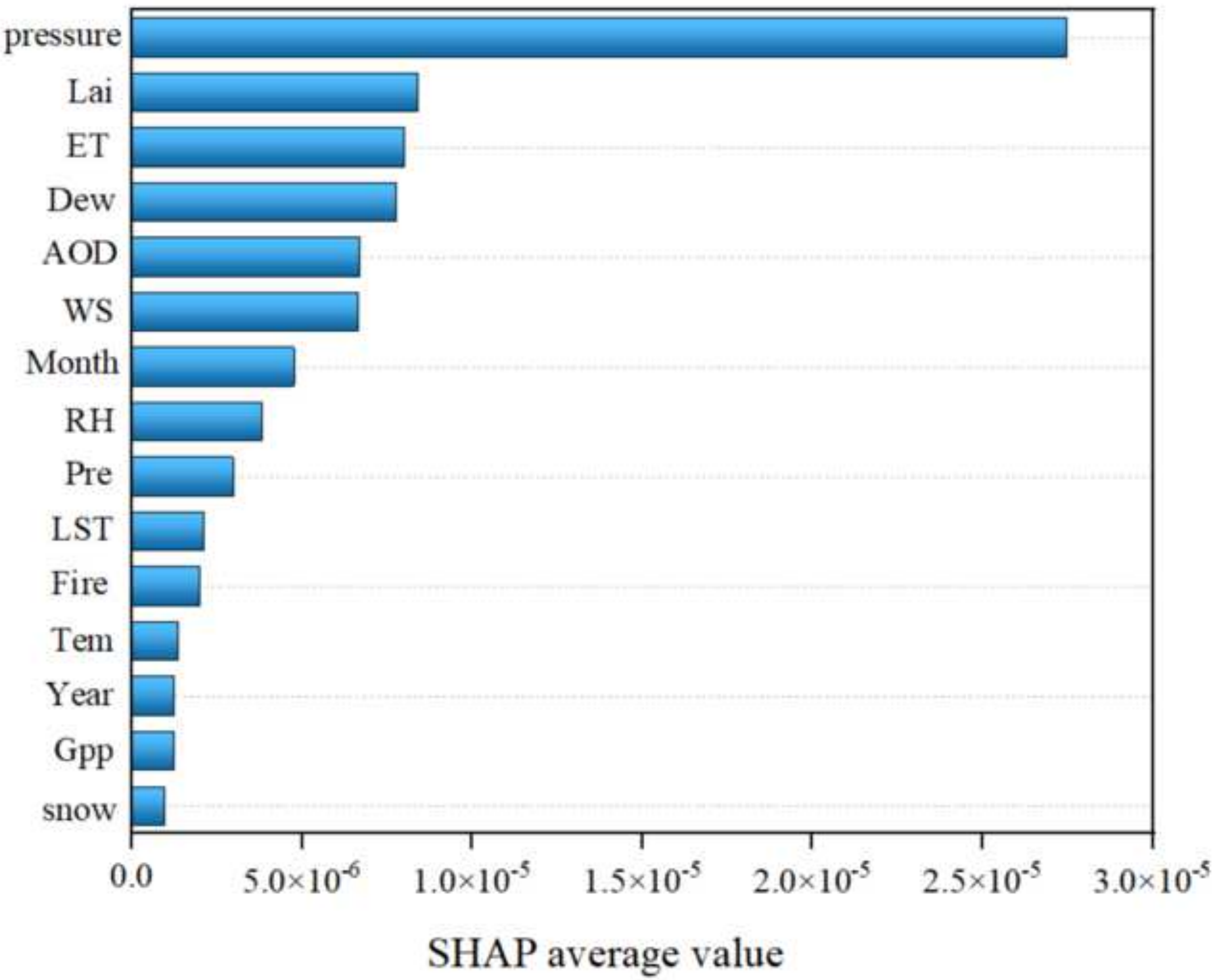

Click here to access/download;Figure;Figure_11.tiff

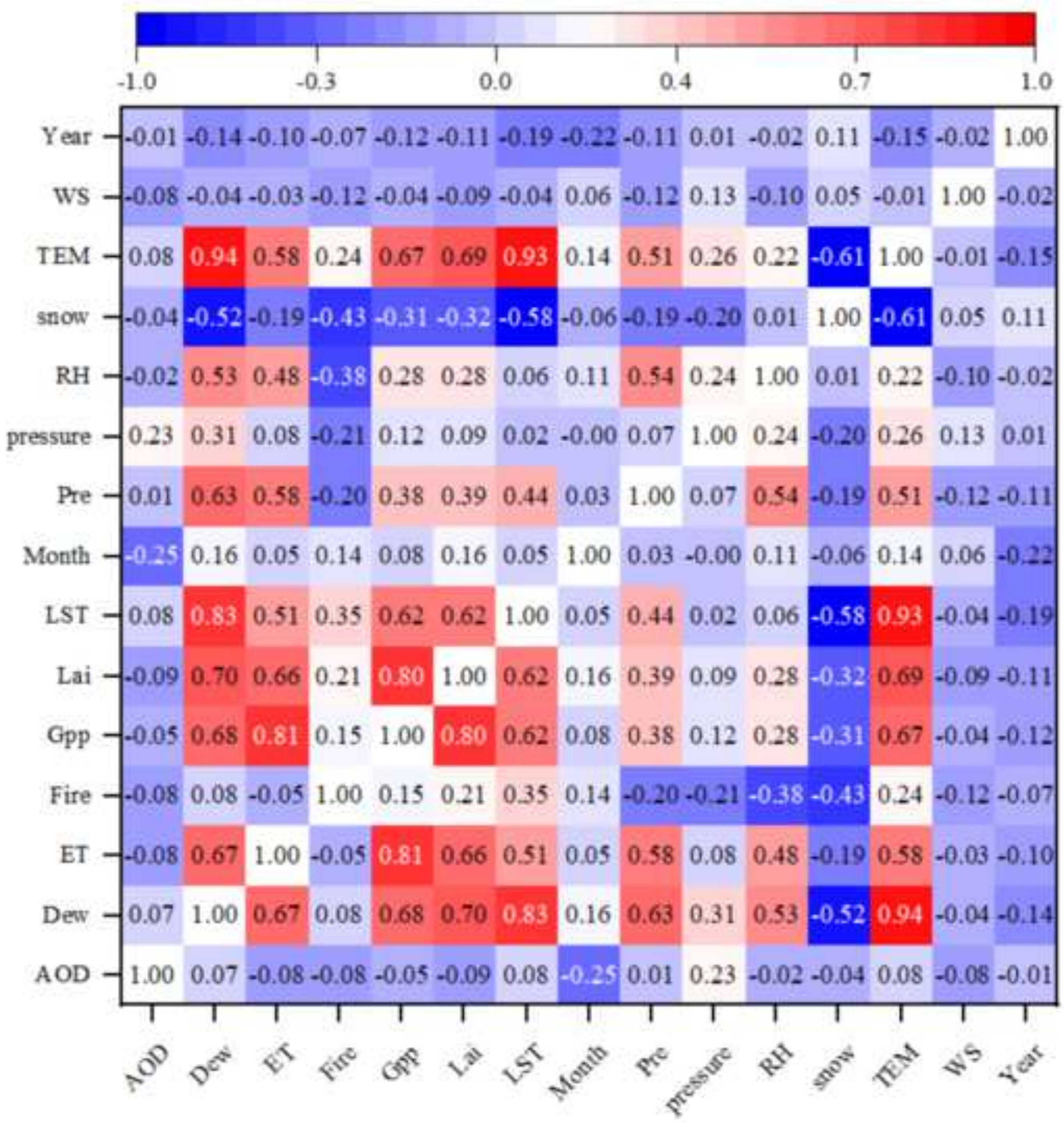

Click here to access/download;Figure;Figure_10(a).tiff

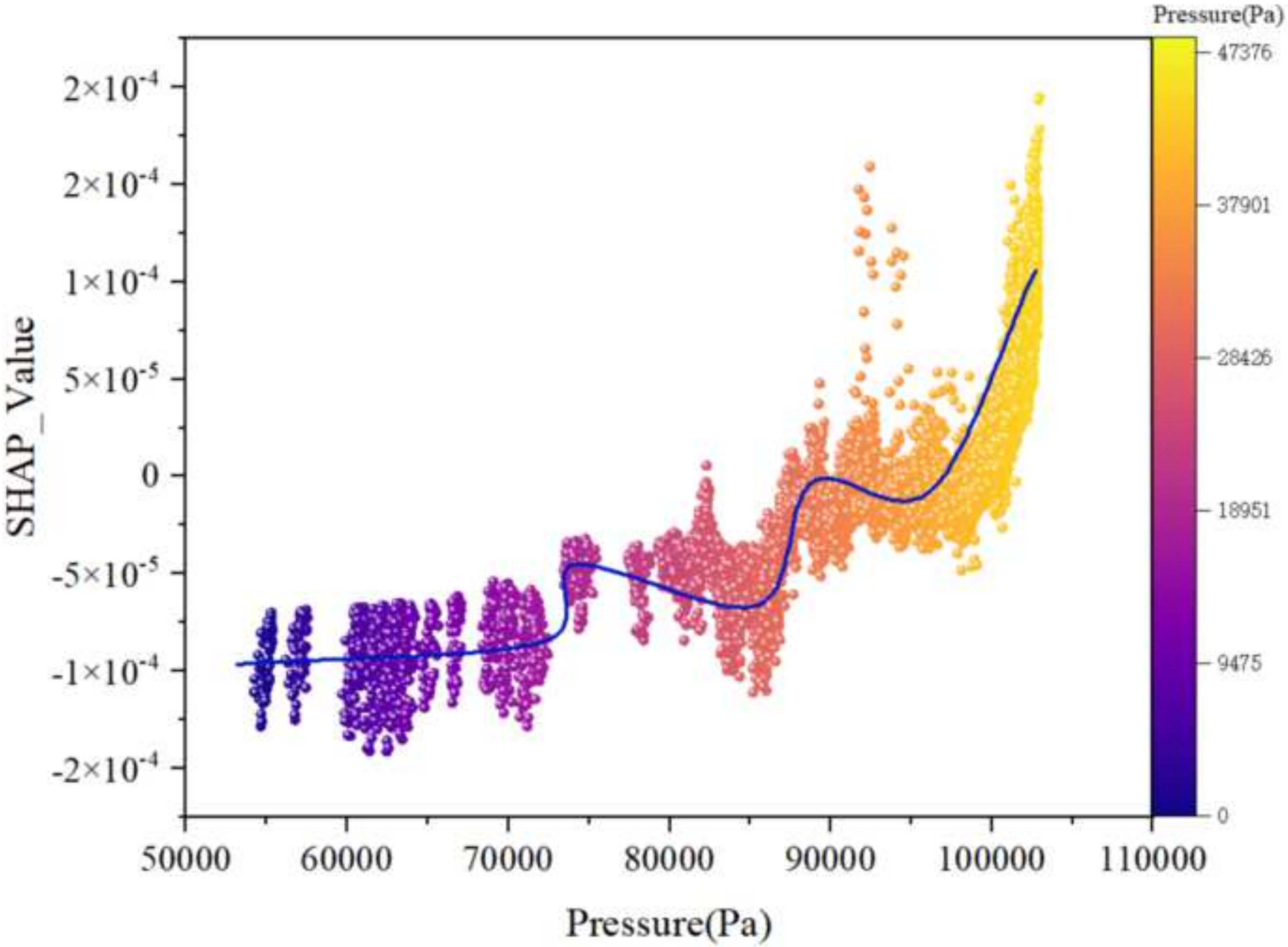

Click here to access/download;Figure;Figure_10(b).tiff

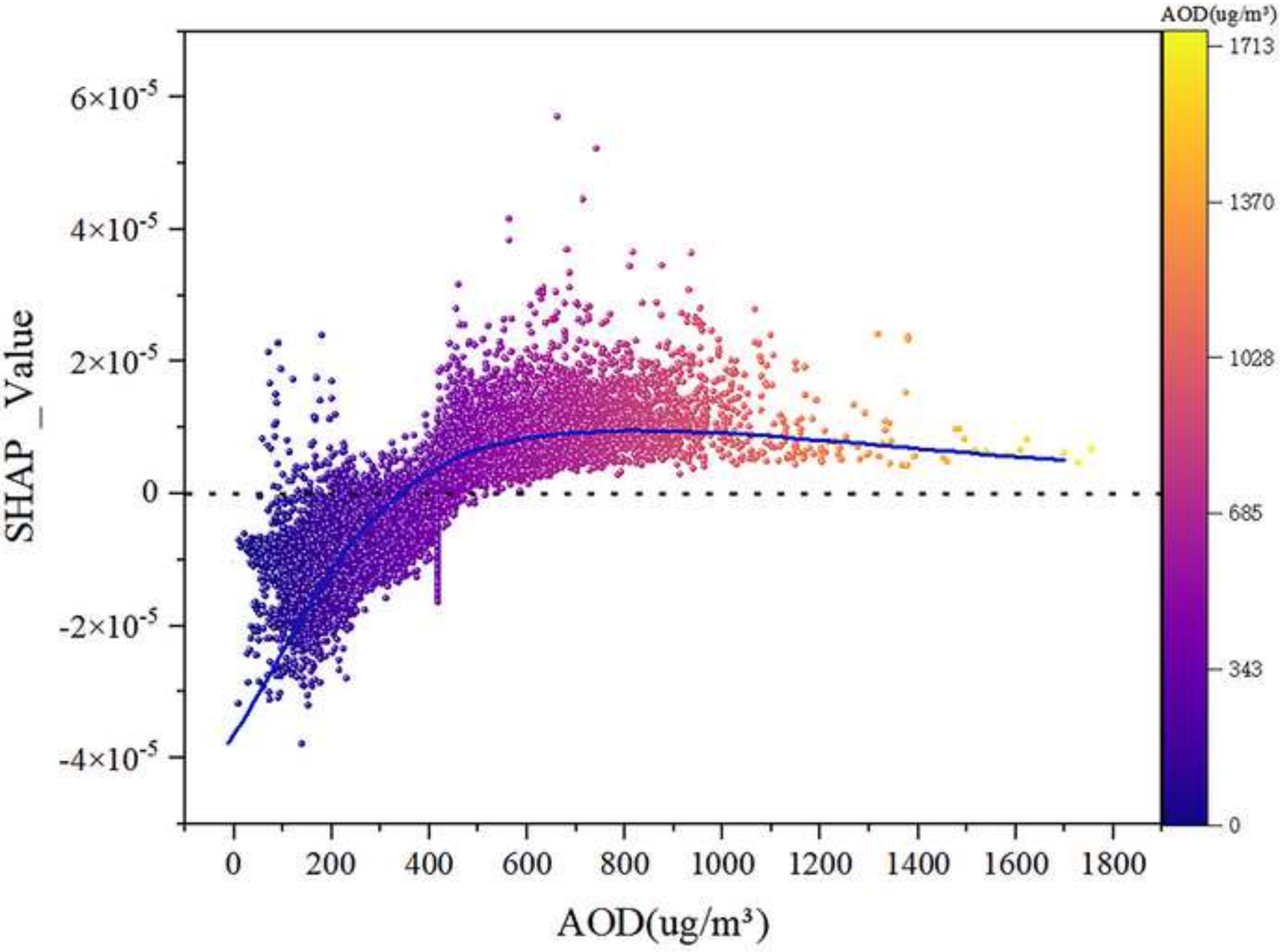

Click here to access/download;Figure;Figure_10(c).tiff

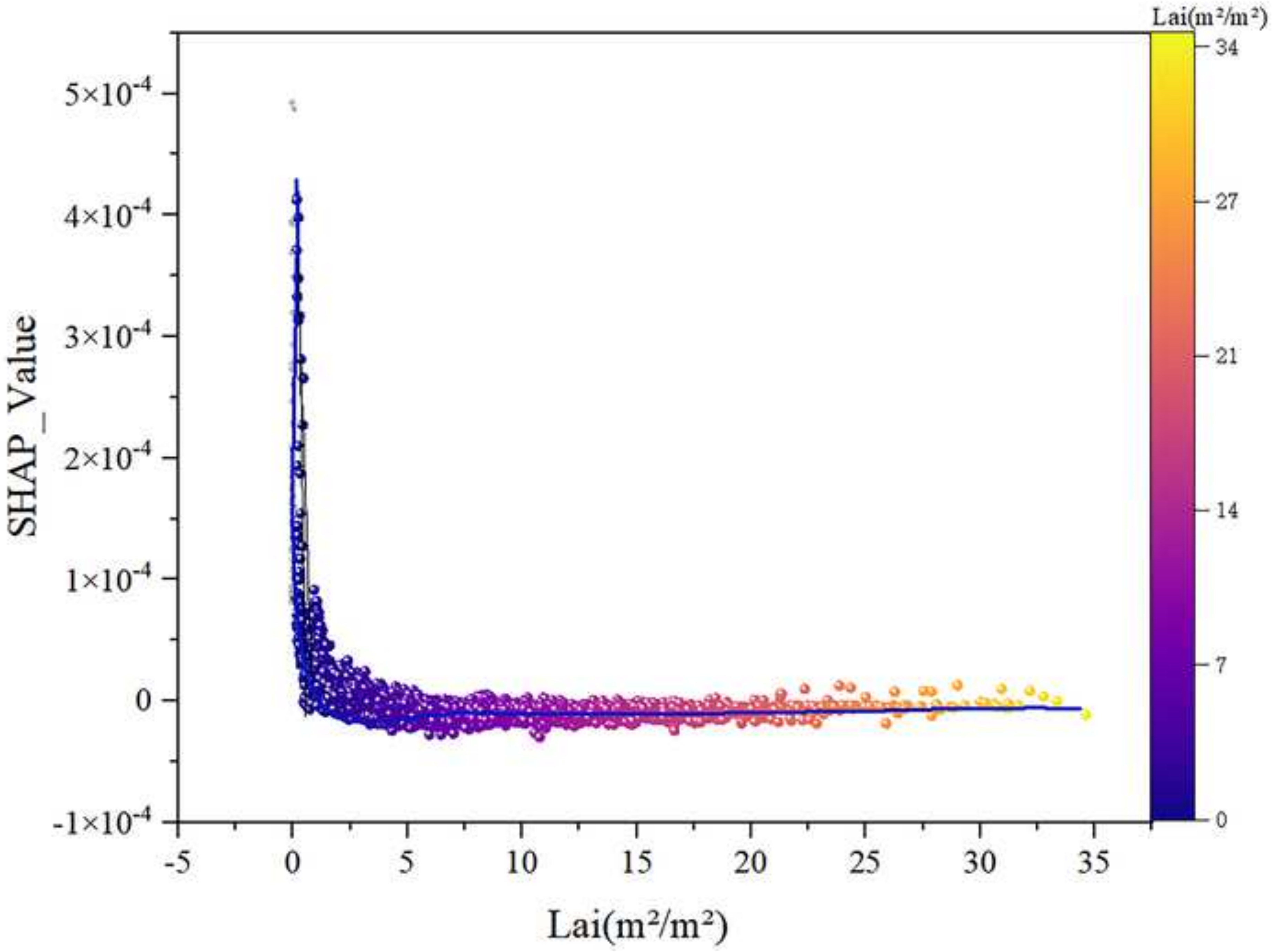

Click here to access/download;Figure;Figure_10(d).tiff

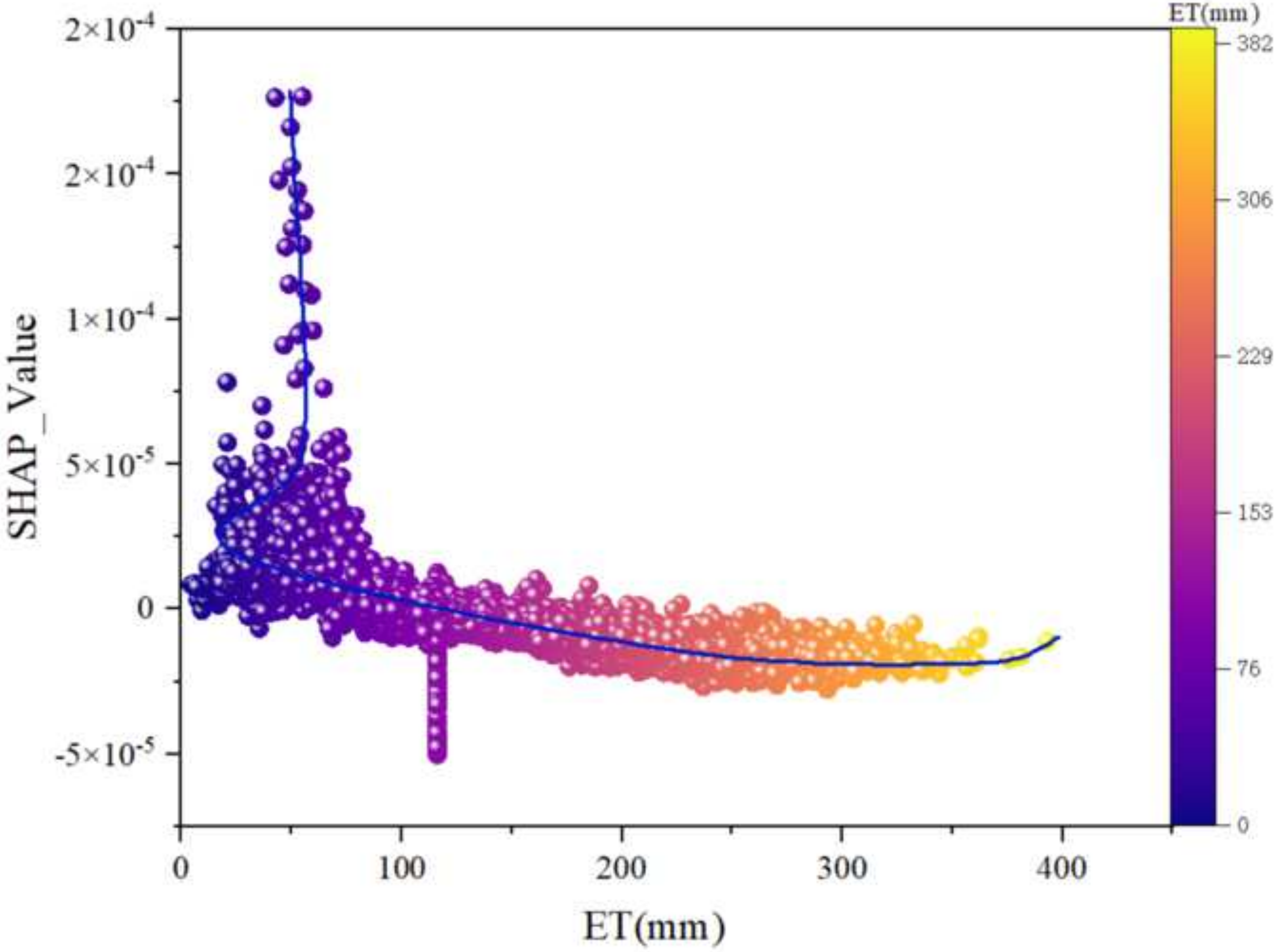

Click here to access/download;Figure;Figure_10(e).tiff

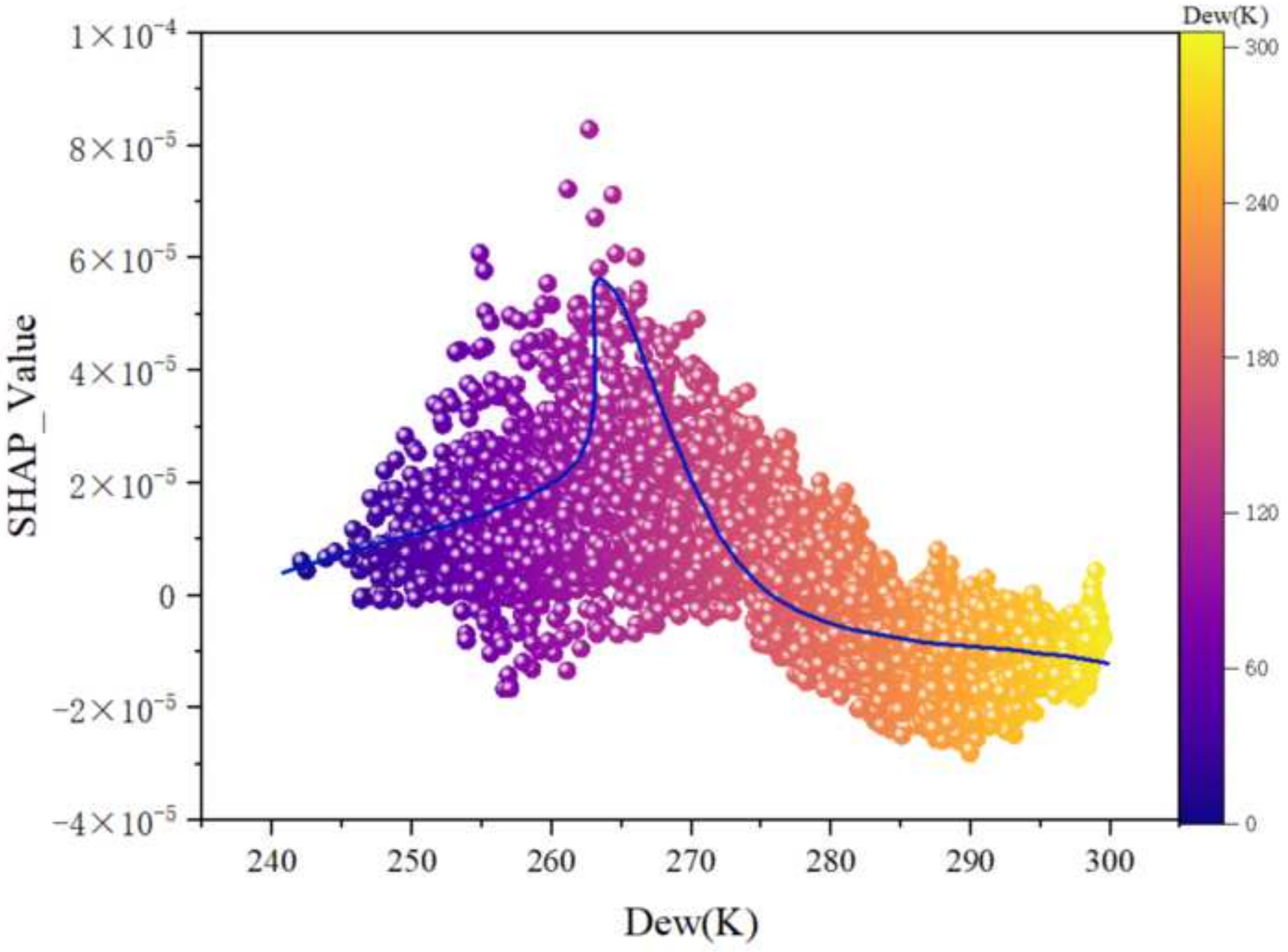

Click here to access/download
Supporting Information - Compressed/ZIP File Archive
Supporting Information.zip
