# Supplementary material for: Nitrogen dioxide pollution in 346 Chinese cities: Spatiotemporal variations and natural drivers from multi-source remote sensing data
Source: PLoS One. 2025 Nov 7;20(11):e0334535. doi: 10.1371/journal.pone.0334535 (PMC12594365; doi:10.1371/journal.pone.0334535)
Supplement: Attachment — Submitted filename: PONE-D-25-30016_reviewer_to authors.pdf [file pone.0334535.s007.pdf]

Dear Authors,

The proposal of **"Spatiotemporal Dynamics and Multidimensional Drivers of Nitrogen dioxide concentration Pollution in 346 Chinese Cities: A Remote Sensing Data Fusion Approach"** presents statistical and local spatial autocorrelation analyses to investigate the spatial-temporal distribution and variation of nitrogen dioxide concentration in urban areas along the Chinese Territory in a period of five years. Additionally, the study applied a machine learning methodological approach to analyze the influence of 15 factors related to climatic and land cover properties on the tropospheric NO2 behavior.

Major observations

The study assesses a wide variety of factors, most of them climatological (eight climatological factors, four factors related to land cover, one factor related to land cover-atmosphere interaction, and two temporal factors); however, the authors do not detail the influence of emission sources and atmospheric chemical interactions of pollutants emitted from these urban centers, which are widely documented as the main factors driving the behavior of tropospheric NO2.

Differentiation of NO2 behavior according to regional climatic characteristics is proposed, however, it is not described or discussed in the document.

Regarding methodology, the use of machine learning is novel due to its growing acceptance as an exploratory method for identifying factors controlling the spatiotemporal behavior of air pollutants. In particular, Random Forest, combined with a predictor evaluation method such as SHAP, offers a non-analytical approach to understanding the interactions of this pollutant based on available input information.

In this sense, it appears to be a relevant and well-implemented method for assessing climate, land cover, land cover-atmosphere interactions, and temporal factors; however, its potential to include additional factors such as predominant emission sources (mobile or stationary sources), as well as the interactions of NO2 with other pollutants such as PM2.5 or O3 itself in the troposphere, has been underutilized.

The information sources for NO2 (SP5), MODIS, and ECMWF/ERA5, along with their inherent uncertainty, were not described in detail. Therefore, questions remain about the impact of source uncertainty and its role in the assessment process, as well as about the response of the prediction model. The model is also not validated with information derived from local grid monitoring sources, or at least from estimates such as emissions inventories.

Therefore, it is advisable to specify the research objective in such a way that it emphasizes the evaluation of the spatiotemporal behavior of NO_2, particularly in relation to climatic factors and their relationship with land cover. Or, conversely, to rethink and expand the research, at least on the role of emission sources (by source type) and to delve deeper into the analysis of the specific weight of the country's climatic diversity.

Minor observations

It is important to explain why the seasonal analysis of the data was chosen instead of monthly. This provides greater temporal resolution for the time series analysis regarding trends and variability.

Nothing is mentioned or highlighted about the effect of hydroelectric plants or forest fires, much less the emission sources, which are primarily related to tropospheric NO2 air pollution. The use of Sentinel offers such spatial resolution that these emission sources can be spatially delimited and their regional influence highlighted.

It should be regard the uncertainty inherent in the data source (Sentinel-5).

It is suggested that complementary sources of information, such as monitoring systems or national pollutant inventories, be consulted to corroborate and discuss the marked seasonal and spatial contrasts.

Punctual observations (all of them in the manuscript file)

Title

In reality, this work does not address the dynamic behavior of atmospheric NO2 chemistry, but rather an analysis of its transient behavior on a monthly or seasonal scale.

This is important to consider, both in the title and in the conceptual basis that underpins this research, whether for correcting or presenting results and developing the discussion around the physical and chemical phenomena that govern the mechanisms and reactions in the troposphere, the products and sub-products resulting from its interaction with specific environmental conditions, as well as its relationship with emission sources.

Abstract

Few lines relate to the methodology efficiency would reinforce the abstract.

In four years, is difficult establishing a trend pattern with specific breakpoints... It should be managed as interannual variation linked to a specific weather proxy.

This temporal variation is similar over all the cities? It should be significant differences by region!

Introduction

Please indicate what this data are and their sources of information!

Is this a reference? It's not included in the reference list!

Materials and Methods

These can easily be separated into two subheadings to address them separately and go into more detail if needed!

This term is uncommon! Could it be a subarctic region?

Sorry but I didn't understand what you mean!

It is important to describe: 1) The data source and the factors that generate uncertainty in the Sentinel 5P product, 2) The time window of the source data for estimating seasonal and annual averages, 3) Its characteristics: level product, spectral regions, pixel size, local time measurement, standard unit of measurement (even more so if tropospheric NO2 is presented in this work with a different numerical scale), 4) Accuracy estimates, 5) Quality assurance value (qa_value) for interpreting data quality (e.g., pixel qe_value greater than 0.75 or 0.5 for cloudy scenes) and, 6) Advantages and disadvantages.

Assuming we are talking specifically about tropospheric NO2... For example, it does not make any difference whether they are using vertical column density measurements, taking into account the differences between NO2 (slanted column density) vs. NO2 (vertical column density), or stratospheric vertical column vs. tropospheric vertical column.

It is worth placing the access link to the database used.

It is worth highlighting why it is better to use Sentinel-5P Offline L3 NO2 instead of Sentinel-5P Near Real-Time NO2 or Reprocessed (RPRO-NO2), as well as their advantages and disadvantages.

Which band(s) were used for the analysis? Explain your choice.

Why did you found the centroid? Please, it is necessary conclude this idea.

It's necessary to follow the journal's hierarchical format; it's confusing. It gives the impression, and it seems, that it's the methodological description, but it doesn't seem to be organized in a hierarchy according to the journal's format. I mention this because it could cause confusion if the hierarchical order of a section heading isn't clearly defined.

Explain this idea in more detail. Clarify that it's a limitation for spatial and temporal analysis, but from there, move on to the next sentence with the problem solved. It's not clear if or how you processed that data.

What attributes are evaluated? The Sentinel-5P series or the parameters in Table 1? Is the analysis performed layer by layer, i.e., individually?

Regardless of whether cluster analysis is applied to the Sentinel series or to the data layers in Table 1, it is important to consider whether the mean, median of a fixed time scale (e.g., monthly average), or snapshot (image/band) is being evaluated.

Please refer to the corresponding equation.

Did you mean: clusters?

Results

It could be suppressed!

This fits into the methodology section.

This is already summarized in Table 2. It is suggested to delete it.

This is an interpretation that should be moved and discussed in the discussion section!

It is necessary to include graphic information or a reference about this argument!

Annual NO2 autocorrelation maps would help highlight clusters with their own degree of significance, rather than Figures 5a-e.

This line is not defined on the maps, nor is it previously mentioned!

There is no map that delineates these natural features (geomorphological, rivers, or deltas), climatic regions, watersheds, or specific elements such as cities that stand out as highly polluted urban centers.

Units should be indicated in the caption of this image. A multi-color scale would help highlight cities or regions with critical concentrations, rather than urban density.

This cluster classification, combined with the location of the cities and their relationship to the climatic region, would provide elements for discussion on the influence of this factor, which was highlighted at the beginning of the study area section.

Is it necessary to explain what this refers to?

This is unclear! Furthermore, there are no references to support this type of argument.

The inset in this image appears to show a larger (zoomed-out) region not shown on the map. The "changed many times" class is indistinguishable from the "No significant" class; please change the dot color or font. Note on formatting this figure type (map). If presented as a map, please note the following: 1) Incorporate the reference system and map projection information, 2) Add a grid or marks to the effective map area, 3) Complete the coordinate information in the lower right ribbon.

Unless this line of thinking is part of the partial conclusions drawn from a detailed analysis of seasonal variability... It is difficult to assume these relationships without supporting data. No correlation analysis or multifactorial comparison, to mention a few examples, is presented that would allow us to understand or assume this causal relationship.

This subheading is very long, please work on summarizing it and the subheadings for sections 3.1 and 3.2.

At this point in the document, there is no evidence of NO2 measurements from monitoring systems or pollutant inventories that corroborate or validate the model's results. If the training and validation process were conducted using the satellite data itself, the inherent uncertainty of the Sentinel 5P product with respect to its spatiotemporal analysis becomes relevant.

What are these observed values? They do not refer to any data source or local NO2 monitoring system. They are not previously mentioned, nor are data or access links presented.

On the “Y” axis, what is the validation data source?

It is not necessary here, this information is in Figure 9. Perhaps the most important factors could be mentioned here.

Since remote sensing of atmospheric gases involves the study of complex masses of substances in a dynamic environment, the estimation of a gas such as NO2 is subject to a wide variety of phenomena and factors that influence its spectral response. In this regard, it is necessary to assess whether any byproducts (e.g., real-time snow and ice extent, wind direction, surface pressure, vapor pressure, albedo) from other satellite platforms (e.g., MODIS) or models (GEOS-5) favor the collinearity of some variables identified in this study with the Sentinel-5P Offline L3 NO2 satellite measurements.

It is interesting to see the relationship between variables and how they play a greater role in predicting NO2 concentrations. However, since prediction models based on machine learning or deep learning are codependent on the volume of data and their own analytical and predictive capacity, a physical or chemical explanation is necessary to broaden the discussion regarding for instance, the climatological gradient according to the region in which a city or cluster of cities is located.

What was the criterion for choosing between AOD and wind? Why were both not included in the analysis and discussion?

Please review the measurement system in this and the other figures to ensure it conforms to conventional expressions. For example, atmospheric pressure is commonly expressed in hPa or millibars.

Atmospheric pressure is commonly expressed in hPa or millibars.

It is necessary to label the SHAP value units, a brief description in the figure caption, and in the body of the document, indicate what it means for each of the variables presented (if the value is different between variables).

Discussion

I understand that the RF model identifies the weight of influence based on the factors or variables that one includes in the modeling process, therefore the identification of key factors is subject to our discretion and not to the analytical capacity of the model.

Please review the color scale, it is incomplete and does not seem to correspond to the classification in the matrix.

Why was the coefficient of determination not chosen?

A value lower than or close to 0.7 is considered moderate or weak. Why did you treat these values as strong correlations? Under what criteria are they considered strong correlations?

Please be clear and concise. This is a correlation matrix.

Seasonal change is directly related to meteorological factors and their interactions (available light-temperature-humidity), and to atmospheric pressure at the synoptic scale. Given that these seasonal changes strongly influence changes in NO2 concentrations, why don't these environmental factors appear at the top of the list in the SHAP analysis? How do you explain such relationships?

This is not clearly noticeable!

The literature widely suggests that emission sources, atmospheric chemical reactions, and meteorological processes are the main factors controlling NO2 concentrations in the troposphere. It is surprising that the LAI appears as one of the main factors, so I believe its importance should be emphasized in the manuscript's statement, discussion, and conclusions.
