# Supplementary material for: Nitrogen dioxide pollution in 346 Chinese cities: Spatiotemporal variations and natural drivers from multi-source remote sensing data
Source: PLoS One. 2025 Nov 7;20(11):e0334535. doi: 10.1371/journal.pone.0334535 (PMC12594365; doi:10.1371/journal.pone.0334535)
Supplement: Attachment — Submitted filename: yang_plos_one_comments.docx [file pone.0334535.s005.docx]

**Major Comments**

I thank the editors and the authors for the opportunity to review this very stimulating study that applies random forest modeling to predict column NO_2_ over a set of cities in China. The authors leverage SHAP to determine the most influential variables on NO_2_ concentrations in their model and perform a spatial autocorrelation analysis. I particularly enjoyed this latter component and think it will be useful for future work to cite as a method for uncovering how predictive variables influence ML outcomes for air pollution modeling.

I think that overall, this study is very interesting and that it will eventually make a great contribution to PLOS One; however, in its current state it still feels a little raw and missing crucial components. My largest issue is that the authors comment multiple times on how emissions patterns and anthropogenic features could be driving the seasonality and spatial patterns in NO_2_; however, these variables are crucially excluded from their RF model which primarily focuses on vegetation and meteorological conditions. I would encourage the authors to add some anthropogenic variables associated with NO_2_ (e.g., road systems, built environment, population density, bottom-up emission inventories, etc.) before making these conclusive statements. Alternatively, they could exclude these discussions from the manuscript.

It would also be useful for the authors to more clearly plot the predictions of NO_2_ from their model. As it is currently plotted, it appears that there is no real spatial variation but I think that has more to do with the color scale than anything else.

Lastly, I encountered many grammatical and formatting mistakes in this manuscript including entire sections that appeared to have no relation to the area of the text that they were in (e.g., L489). I suggest that the authors thoroughly review their manuscript and ensure that it is more polished before resubmitting.

There were no line numbers in the original document, so all of the line numbers referred to below were determined using the “line number” feature in Microsoft Word.

**Minor Comments**

L12-13: “data” is said twice

L16: “utilized” generally refers to using something beyond its intended purpose which I would argue is not the case here, I suggest replacing with “used”

L17: “and etc.” is incorrect, it should be “etc.”

L24: I wonder if the AOD impact is more due to reduced coverage? I’ll keep reading to see.

L25: Can you be a little more specific about how these findings could benefit policymaking / pollution remediation?

L45: “threatens” not “threats”

L48-L50: I would rephrase this as NO2 is both a pollutant in its own right (see Anenberg et al. 2022, Achakulwisut et al. 2019, or the most recent GBD study) and a precursor to other pollutants O3 via photochemical reactions with VOCs and PM2.5 via nitrate formation.

L50-53: You never define the NO2 abbreviation

L64-65: Check citation, this looks incorrect

L80-85: I am a little concerned about the lack of land use data (e.g., built environment and road systems) in this model as many studies (e.g., Larkin et al., 2017, 2023; Nawaz et al., 2025) have shown strong dependence on these features. Is there a reason that they are excluded? These can be easily extracted using the python osmnx library (for road system data) and built environment data is available on GEE.

L87: Maybe mention specifically you are pulling L3 TROPOMI data here?

L117-122: This seems out of place.

L128: Actually, since Aug 2019 it’s even better at 5.5 x 3.5 km

L133: It’s probably worth mentioning somewhere in this paragraph that TROPOMI measures at the same time during the day (early afternoon) each day, so these findings are representative of early afternoon NO2.

L147: How are you determining low quality pixels?

L238: Maybe I am misunderstanding, but it looks like you have only one anthropogenic index (GPP)? If so you should change this to “an anthropogenic index” instead. This is connected to my earlier point that more land-use information could be useful to include.

L257: It would be useful to comment somewhere in this methods section how well correlated TROPOMI L3 data is with surface-level monitoring. I am guessing prior studies have done this for these cities before?

L261: How was this homogenization done explicitly? I ask as there are specific data unavailability in the daily TROPOMI data so caution is needed to ensure that averaging is not specifically biased towards certain seasons (and if it is, e.g., a cloud bias, this should be commented on here).

L296-299: I am somewhat surprised that although Autumn has the second highest concentrations, it has the lowest F value, my intuition is that generally seasons with higher NO2 would have higher interannual variability. It’s probably wroth commenting on why this is happening in the manuscript.

L301-305: This sentence has some mistakes. Meteorological stagnation is not a “seasonal emission pattern”. I suggest rewriting this.

L306: How do you know that these anomalies are “primarily due to coal combustion … and increased emissions from fireworks”. I generally think that the seasonality plays a bigger part of the increased NO_2_ in winter (i.e., from a weaker photochemical sink).

Figure 4: I wonder if you could cap outlier values here to make the interannual variations by season clearer? And then comment on this in the caption. Right now its hard to determine this.

Figure 5: Are these the training data or the predicted values? This needs to be clarified. Regardless it’s difficult to identify any spatial trends using the current colormap / figure style. I wonder if zooming in on a few regions and using a more dynamic color range (e.g., gist_ncar, cube_helix, jet, etc.) might allow you to pick out clearer spatial patterns?

L412: I think it would be helpful to include a map of the predicted values (if this is not what Figure 5 is).

L436: Isn’t SHAP mesasuring the inverse (i.e., the sensitivity of NO2 to AOD **not** the sensitity of AOD to NO2)? Also if this is true the formation of secondary inorganic aerosol is mediated by meteorological and seasonal variation, did you look into this?

L482-483: I don’t think you can really say this conclusively without looking into land-use features specifically NO_2_ emissions. If you want you could soften this sentence by restricting it to your model, but generally I don’t believe this is true; i.e., emissions modify NO_2_ concentrations but you can’t really comment on this as these are not considered in your model.

L484: I think instead of “validate” you mean “corroborate” or “confirm”.

L489-493: I am not sure why this is here.

L527 / Table 1: Can you include information in Table 1 on the spatial and temporal resolution (and coverage) of these predictor variables? Oftentimes in ML models the finest resolution data may have the most influence but this does not necessarily mean that these variables have the greatest contribution to the predictor, rather just that they capture more spatial variability.

L557-563: I don’t know if you can conclude any of this based on your study. You don’t look into population data or emissions as predictor variables and GPP has little influence on your model. And the same with coal heating. I would rewrite this to say that these may be causing the spatial clustering and cite studies that have found this in the past.

**References**

Larkin, A., Geddes, J. A., Martin, R. V., Xiao, Q., Liu, Y., Marshall, J. D., et al. (2017). Global Land Use Regression Model for Nitrogen Dioxide Air Pollution. *Environmental Science & Technology*, *51*(12), 6957–6964. https://doi.org/10.1021/acs.est.7b01148

Larkin, A., Anenberg, S., Goldberg, D. L., Mohegh, A., Brauer, M., & Hystad, P. (2023). A global spatial-temporal land use regression model for nitrogen dioxide air pollution. *Frontiers in Environmental Science*, *11*. https://doi.org/10.3389/fenvs.2023.1125979

Nawaz, M. O., Goldberg, D. L., Kerr, G. H., & Anenberg, S. C. (2025). TROPOMI Satellite Data Reshape NO2 Air Pollution Land-Use Regression Modeling Capabilities in the United States. *ACS ES&T Air*, *2*(2), 187–200. https://doi.org/10.1021/acsestair.4c00153
