# Supplementary material for: Nitrogen dioxide pollution in 346 Chinese cities: Spatiotemporal variations and natural drivers from multi-source remote sensing data
Source: PLoS One. 2025 Nov 7;20(11):e0334535. doi: 10.1371/journal.pone.0334535 (PMC12594365; doi:10.1371/journal.pone.0334535)
Supplement: Attachment — Submitted filename: Response to Reviewers.docx [file pone.0334535.s008.docx]

Responses to Reviewers’ Comments for Manuscript PONE-D-25-30016

**Spatiotemporal Dynamics and Multidimensional Drivers of Nitrogen dioxide concentration Pollution in 346 Chinese Cities: A Remote Sensing Data Fusion Approach**

Addressed Comments for Publication to

PLOS ONE

by

Heming Yang

Dear Dr. Editor,

Please find enclosed the revised version of our previous submission entitled “Spatiotemporal Dynamics and Multidimensional Drivers of Nitrogen dioxide concentration Pollution in 346 Chinese Cities: A Remote Sensing Data Fusion Approach” with manuscript number PONE-D-25-30016. We would like to thank you and the reviewers for the valuable comments which help improving the quality of our manuscript. In this revision, we have carefully addressed the reviewers’ comments. A summary of main modifications and a detailed point-by-point response to the comments from Reviewers 1 to 2 are given below.

Sincerely,

Heming Yang

**Note:** To improve the readability of the response letter, the editor's comments are replied to in red text, while the comments from the two reviewers are addressed point by point in blue text and green text respectively..

**Authors’ Response to the Editor**

**(1)I would like to note that one or more reviewers have recommended citing specific previously published works. I kindly advise you to carefully review the suggested references and assess their relevance to your manuscript.**

**Response:** Thank you for your careful review of our paper and your valuable suggestions. Regarding the relevance of the literature you mentioned, we have carefully checked it in accordance with your comments and further improved the citation of relevant literature in the revised manuscript. The specific revisions are as follows:

We have reorganized the literature closely related to the research topic and added the following key references.

**(2)Please ensure that your manuscript meets PLOS ONE's style requirements, including those for file naming.**

**Response:** We sincerely appreciate your valuable guidance on the manuscript formatting. We are pleased to confirm that both the title page and main body have been fully revised to comply with the journal's specifications.

**(3)Please note that PLOS One has specific guidelines on code sharing for submissions in which author-generated code underpins the findings in the manuscript.**

**Response:** We fully agree with your point regarding the openness of the random forest algorithm. The basic code for the random forest used in this paper has been provided in the supporting information (S2_File, S3_File).

The Particle Swarm Optimization (PSO) algorithm adopted in this paper efficiently explores the parameter space by simulating swarm intelligence behavior, and the finally obtained parameter combinations finally obtained are also shown in the supporting information (S1_File).

**(4)Please state what role the funders took in the study.  If the funders had no role, please state: "The funders had no role in study design, data collection and analysis, decision to publish, or preparation of the manuscript."**

**Response:** Thank you for your question. Regarding the issue of funders, in addition to [YDZJ202401520ZYTS], we have added another [YDZJ202301ZYTS234]. The funders have provided us with professional knowledge in the field of paleoenvironment-paleoclimate co-evolution, and their professional insights have significantly deepened our understanding of climate change and ecological environment. The funders had a role in preparation of the manuscript.

**(5)Please note that your Data Availability Statement is currently missing the accession number of each dataset OR a direct link to access each database.**

**Response:** The data that support the findings of this study are available in Google Earth Engine. The data acquisition link is: <https://earthengine.google.com/> .We have also marked the data source link in the manuscript. Furthermore, we have made this dataset available on the figshare platform with the DOI: 10.6084/m9.figshare.29948741. We kindly ask that you help add the link to this dataset in an appropriate position. Thank you again for pointing out our issues.

**(6)We note that Figures 1, 2, 5, and 7 in your submission contain map images which may be copyrighted.**

**Response:** The administrative division data in GeoJSON format is sourced from the National Geospatial Information Public Service Platform (Tianditu), with the website: https://cloudcenter.tianditu.gov.cn/administrativeDivision. The map approval number is GS (2024) 0650. The data coordinates are in GCS_WGS_1984. The city points are derived from the Global Artificial Impervious Area (GAIA) dataset, Version 10 (v10), which was developed by Tsinghua University/Fine Resolution Observation and Monitoring of Global Land Cover (FROM-GLC). Gong, P., Li, X., Wang, J., Bai, Y., Chen, B., Hu, T.,& Zhou, Y. (2020). Annual maps of global artificial impervious area (GAIA) between 1985 and 2018. Remote Sensing of Environment, 236, 111510(<https://doi.org/10.1016/j.rse.2019.111510>)

**(7)Please include a caption for figures 10a, 10b, 10c, 10d.**

**Response:** Thank you for your rigorous review of the paper's details! Regarding the issue of labeling in Figure 10 that you raised, we have added clear axis labels and legend explanations to all subfigures in Figure 10. We again appreciate your professional suggestions on the graphical issues. (L449-L452)

**(8)Please include captions for your Supporting Information files at the end of your manuscript, and update any in-text citations to match accordingly.**

**Response:** Thank you sincerely for your careful review and valuable suggestions! Regarding the labeling of the Supporting Information, we have carefully improved it: complete captions have been added for all supporting materials at the end of the manuscript. Each caption now includes:

- File name and format (e.g., "Supplementary_Table1.xlsx")

- Brief description of content (e.g., " Annual average concentration of nitrogen dioxide ")

**Concluding Response to the Editor:**

Thank you for your valuable comments on our manuscript. We have done our best to incorporate changes to reflect the suggestions, which allowed us to improve the quality of our work.

**Authors’ Response to Reviewer 1**

**Major Comment 1**

My largest issue is that the authors comment multiple times on how emissions patterns and anthropogenic features could be driving the seasonality and spatial patterns in NO_2_; however, these variables are crucially excluded from their RF model which primarily focuses on vegetation and meteorological conditions. I would encourage the authors to add some anthropogenic variables associated with NO_2_ (e.g., road systems, built environment, population density, bottom-up emission inventories, etc.) before making these conclusive statements.

**Response:** Thank you for your valuable feedback on our manuscript. We appreciate your suggestion to incorporate anthropogenic factors and would like to address your concerns point by point:

1. Regarding the primary focus of our model:

The study primarily examines meteorological and vegetation factors, as these constitute the dominant influences within our research framework.

2. Concerning additional anthropogenic factors:

**Population Density:**

The most recent publicly available demographic data currently extends only to 2022, while our research period focuses on 2023. This temporal misalignment would compromise the analytical robustness of our findings."

**Built Environment：**

This factor is inherently represented through our artificial impervious surface metric, which explicitly captures urban built-up areas (including structures and infrastructure).Global Artificial Impervious Area (GAIA) dataset, Version 10 (v10). Developed by: Tsinghua University/Fine Resolution Observation and Monitoring of Global Land Cover (FROM-GLC)

**Emission Inventories:**

Despite extensive efforts, the most recent verified emission data (e.g., MEIC for China) currently extends only to 2020. This misalignment with our 2019–2023 study period precludes statistically robust inclusion. For example, Multi-resolution Emission Inventory for China，MEIC.

**Road Networks:**

Given the complexity of China’s multi-tier transportation systems (expressways, national/regional highways, railways), we determined this variable warrants a dedicated study to properly analyze its mechanistic impacts on air pollutants. We are designing such follow-up research.

In this article, we will appropriately cut out the comments on anthropogenic factors and focus on discussing the effects of natural factors on NO_2_ concentrations.We sincerely appreciate your insightful suggestions, which have strengthened our manuscript's scholarly rigor.

**Major Comment 2**

It would also be useful for the authors to more clearly plot the predictions of NO2 from their model. As it is currently plotted, it appears that there is no real spatial variation but I think that has more to do with the color scale than anything else.

**Response:** Thank you for your feedback.

Following your suggestion, we replaced the color ribbon to revise the images, but found that the effect was not very satisfactory. Therefore, we speculate that this is because the cities with high concentrations and those with low concentrations remained relatively stable over the five years, resulting in no significant differences in their spatial distribution. We have added a climate zoning map for combined analysis.

Accordingly, in the analysis results, we have removed the part about "spatial changes over the five years" and replaced it with "spatial commonalities in the distribution of urban nitrogen dioxide concentrations over the five years", so as to illustrate the high and low concentration areas of nitrogen dioxide in terms of spatial distribution.

**Major Comment 3**

Lastly, I encountered many grammatical and formatting mistakes in this manuscript including entire sections that appeared to have no relation to the area of the text that they were in (e.g., L489). I suggest that the authors thoroughly review their manuscript and ensure that it is more polished before resubmitting.

**Response:** Thank you for pointing out my formatting issues, I will seriously check and polish them up. For L489, the content here is a small summary of the above part, but obviously this paragraph is long and the summary is not in place. Thank you for your comment. I have cut this passage and changed it.

**Minor Comment 1**

L12-13: “data” is said twice.

**Response:** Thank you for the comment.

We have made modifications to address this problem. See L18 for details.

**Minor Comment 2**

L16: “utilized” generally refers to using something beyond its intended purpose which I would argue is not the case here, I suggest replacing with “used”

**Response:** Thank you for the comment.

We have made modifications to address this problem. See L20 for details.

**Minor Comment 3**

L17: “and etc.” is incorrect, it should be “etc.”

**Response:** Thank you for the comment.

We have made modifications to address this problem. See L19 for details.

**Minor Comment 4**

L24: I wonder if the AOD impact is more due to reduced coverage? I’ll keep reading to see.

**Response:** AOD data acquisition is affected by cloud cover, which has a significant impact on monthly analysis(e.g. Satellite remote sensing of aerosol optical depth: advances, challenges, and perspectives.2020. [https://doi.org/10.1080/10643389.2019.1665944)](https://doi.org/10.1080/10643389.2019.1665944). The time scope of our study in this article is seasons and months, and AOD is only selected as an influencing indicator to analyze its relationship with changes in NO_2_ concentration.

**Minor Comment 5**

L25: Can you be a little more specific about how these findings could benefit policymaking / pollution remediation?

**Response:** Thank you for the comment.

This study mainly identifies the main factors affecting NO_2_ concentration through models, reveals the main causes of urban NO_2_ pollution in China, and helps policy makers to make targeted main measures to reduce urban air pollution. The specific content has been supplemented in article L30-L33.

**Minor Comment 6**

L45: “threatens” not “threats”.

**Response:** In response to this problem, we have made modifications in L39 and L45, thank you for pointing it out.

**Minor Comment 7**

L48-L50: I would rephrase this as NO_2_ is both a pollutant in its own right (see Anenberg et al. 2022, Achakulwisut et al. 2019, or the most recent GBD study) and a precursor to other pollutants O_3_ via photochemical reactions with VOCs and PM_2.5_ via nitrate formation.

**Response:** Thank you for your suggestion, this sentence has been revised according to your proposed words, and the reference is marked at the end of the paragraph.(L41-L43)

**Minor Comment 8**

L50-53: You never define the NO_2_ abbreviation.

**Response:** Thank you for pointing out the problem, regarding the definition of the abbreviation of NO_2_ we added in L41.

**Minor Comment 9**

L64-65: Check citation, this looks incorrect.

**Response:** Thank you for pointing out the problem. We carefully considered this passage and found that it can be deleted without affecting the meaning expressed in the article, and at the same time, it makes the article look more concise.

**Minor Comment 10**

L80-85: I am a little concerned about the lack of land use data (e.g., built environment and road systems) in this model as many studies (e.g., Larkin et al., 2017, 2023; Nawaz et al., 2025) have shown strong dependence on these features. Is there a reason that they are excluded? These can be easily extracted using the python osmnx library (for road system data) and built environment data is available on GEE.

**Response:** Thanks for your comment, I'll answer the question. This issue was also mentioned in your comment above, which shows that you attach great importance to this issue. The five-year road system (such as high-speed rail, railway, highway, etc.) needs to be studied separately due to the huge amount of data and engineering, which is difficult in research; For the problem of built environment, the city point selected in this study is the urban built boundary obtained on the GEE platform, and the impermeable surface data adopted. The link to the data set has been attached to the above comment.

**Minor Comment 11**

L87: Maybe mention specifically you are pulling L3 TROPOMI data here?

**Response:** According to your reminder, it is indeed found that TROPOMI data needs to be mentioned, and the modified content can be viewed at L70.

**Minor Comment 12**

L117-122: This seems out of place.

**Response:** This seems is really out of place, because we made a mistake. We added this passage here, and the text of this sentence has been deleted.

**Minor Comment 13**

L128: Actually, since Aug 2019 it’s even better at 5.5 x 3.5 km

**Response:** Indeed, as you said, we have made changes to this problem in L109-L110.

**Minor Comment 14**

L133: It’s probably worth mentioning somewhere in this paragraph that TROPOMI measures at the same time during the day (early afternoon) each day, so these findings are representative of early afternoon NO_2_.

**Response:** For your comment, we seriously incorporate this suggestion into L115 of the paper.

**Minor Comment 15**

L147: How are you determining low quality pixels?

**Response:** Thank you for your professional question. We would like to explain this as follows: Compared to L2 products, L3 products have built-in cloud screening and quality control (with L3 QAFLAGS ≥ 0.75), which reduces invalid values caused by cloud cover in the original L2 data and filters out low-quality pixels(Platnick, S., et al. (2017). "MODIS Atmosphere L2 Cloud Product (06_L2)." NASA MODIS Adaptive Processing System.

<https://doi.org/10.5067/MODIS/MOD06_L2.061>). Therefore, we directly obtained L3 data products for data analysis. This explanation has been added to the manuscript; please refer to L111-L113 for details.

**Minor Comment 16**

L238: Maybe I am misunderstanding, but it looks like you have only one anthropogenic index (GPP)? If so you should change this to “an anthropogenic index” instead. This is connected to my earlier point that more land-use information could be useful to include.

**Response:** Thank you very much for raising the question of influencing factors again. Regarding the issue of GPP, I want to explain it for you. We regard GPP as a natural factor (Chapin III F S, Matson P A, Mooney H A. Principles of terrestrial ecosystem ecology[M]. New York, NY: Springer New York, 2002.). For the question of human factors, I have already replied in the above comment, thank you for pointing it out.

**Minor Comment 17**

L257: It would be useful to comment somewhere in this methods section how well correlated TROPOMI L3 data is with surface-level monitoring. I am guessing prior studies have done this for these cities before?

**Response:** There are indeed many scholars who utilize TROPOMI L3 data in conjunction with surface monitoring, and our study complements the literature on TROPOMI L3 sensors in conjunction with atmospheric pollutant monitoring. （L249-L252）

**Minor Comment 18**

L261: How was this homogenization done explicitly? I ask as there are specific data unavailability in the daily TROPOMI data so caution is needed to ensure that averaging is not specifically biased towards certain seasons (and if it is, e.g., a cloud bias, this should be commented on here).

**Response:** You have mentioned this issue multiple times, which indicates its significance. We would like to explain it again: It is true that cloud cover can affect the accuracy of the data. However, the impact of clouds is more pronounced at the monthly time scale, while our study adopts seasons and years as the time scales for research. Therefore, its influence on the results of this study is relatively minor. Thank you again for your professional suggestions. We are happy to provide further explanations if needed.

**Minor Comment 19**

L296-299: I am somewhat surprised that although Autumn has the second highest concentrations, it has the lowest F value, my intuition is that generally seasons with higher NO_2_ would have higher interannual variability. It’s probably wroth commenting on why this is happening in the manuscript.

**Response:** Thank you for your comment, in response to this problem, firstly, we determined the accuracy of the data, and secondly, reading the relevant literature(Screen, J., Simmonds, I. The central role of diminishing sea ice in recent Arctic temperature amplification. Nature 464, 1334–1337 (2010). <https://doi.org/10.1038/nature09051>), we think that the meteorological conditions (such as wind speed, temperature, precipitation) in autumn are more stable between years, then even if the NO_2_ concentration is higher, its interannual variation may be small and the F value is minimal. We also explain this point in Article L499-L503.

**Minor Comment 20**

L301-305: This sentence has some mistakes. Meteorological stagnation is not a “seasonal emission pattern”. I suggest rewriting this

**Response:** Thank you for your comment. We carefully thought about it, deleted the sentence seasonal pattern, and directly analyzed the research results.

**Minor Comment 21**

L306: How do you know that these anomalies are “primarily due to coal combustion … and increased emissions from fireworks”. I generally think that the seasonality plays a bigger part of the increased NO_2_ in winter (i.e., from a weaker photochemical sink).

**Response:** Thank you for this suggestion, for this issue, we have changed it in article L316-L317.

**Minor Comment 22**

Figure 4: I wonder if you could cap outlier values here to make the interannual variations by season clearer? And then comment on this in the caption. Right now its hard to determine this.

**Response:** Thank you for your comment. Let me explain this problem. The one with more outliers in the figure is winter. The main reason for the high outliers in winter is the heating and fireworks setting off in cities in northern China in winter（Michael Biggart, et al. Modelling spatiotemporal variations of the canopy layer urban heat island in Beijing at the neighbourhood scale.）. In addition, the meteorological conditions in winter are relatively stable, and NO_2_ pollutants are not easy to diffuse. Therefore, NO_2_ is abnormally increased in some areas, and there are many outliers. The occurrence of these outliers is normal.

**Minor Comment 23**

Figure 5: Are these the training data or the predicted values? This needs to be clarified. Regardless it’s difficult to identify any spatial trends using the current colormap/figure style. I wonder if zooming in on a few regions and using a more dynamic color range (e.g., gist, ncar, cube , helix, jet, etc.) might allow you to pick out clearer spatial patterns?

**Response:** These data are the directly obtained five-year annual average concentration data, without involving training values or predicted values. This part of the content aims to illustrate the cities with high and low nitrogen dioxide concentrations across China. Regarding the color band issue of the spatial change map, we have attempted to use other color bands for plotting, but the results were not ideal. Therefore, we have decided to add climate zoning to enhance the visualization effect of the map. Meanwhile, we will focus on discussing the annual commonalities in spatial distribution and reduce the discussion on spatial dynamic changes. This point has also been revised in the title.

**Minor Comment 24**

L412: I think it would be helpful to include a map of the predicted values (if this is not what Figure 5 is).

**Response:** Thank you for your suggestions and professional questions. We used the annual average values for mapping only to analyze their spatial distribution, which does not involve the prediction part. The focus of our study is on the random forest model optimized by particle swarm optimization and the ranking of influencing factors. However, we have improved the distribution map of NO_2_ annual average values by adding climate zones, which can better facilitate the analysis of influencing factors in combination with meteorological factors.

**Minor Comment 25**

L436: Isn’t SHAP mesasuring the inverse (i.e., the sensitivity of NO_2_ to AOD not the sensitity of AOD to NO_2_)? Also if this is true the formation of secondary inorganic aerosol is mediated by meteorological and seasonal variation, did you look into this?

**Response:** Thank you for your professional insights on the manuscript content. Due to the issue with our language expression, we have revised this sentence. You are correct; it is indeed the sensitivity of NO_2_ to AOD. Please refer to L434-L436 for the revised content.

Regarding the research on AOD, let us clarify that we only use AOD as an influencing factor to analyze the changes in NO_2_ concentration, and we have not conducted in-depth discussions on the mechanism of AOD's own changes.

Thank you again for your suggestions. If there are other issues, we will consider adopting relevant feedback and provide explanations for you.

**Minor Comment 26**

L482-483: I don’t think you can really say this conclusively without looking into land-use features specifically NO_2_ emissions. If you want you could soften this sentence by restricting it to your model, but generally I don’t believe this is true; i.e., emissions modify NO_2_ concentrations but you can’t really comment on this as these are not considered in your model.

**Response:** Thank you for your suggestions. After careful consideration following your pointing out, we believe that the expression "urban pollution" is incorrect. We have revised "urban pollution" to "changes in NO_2_ concentration", which can reflect the rigor of the language and the expression of the meaning (L511). Since emission sources are not mentioned in the model, we have deleted the discussion about emission sources, and some necessary discussions are followed by relevant references. Thank you again for your guidance.

**Minor Comment 27**

L484: I think instead of “validate” you mean “corroborate” or “confirm”.

**Response:** Thank you for pointing out this problem. Our research is divided into training set and verification set, and R^2^ is used to confirm the performance of the model. We have changed "validate" to "confirm" for this.（L483）

**Minor Comment 28**

L527/Table 1: Can you include information in Table 1 on the spatial and temporal resolution (and coverage) of these predictor variables? Oftentimes in ML models the finest resolution data may have the most influence but this does not necessarily mean that these variables have the greatest contribution to the predictor, rather just that they capture more spatial variability.

**Response:** Thank you for your suggestions. We have supplemented the temporal and spatial resolutions of the datasets (such as MODIS and ERA5) as well as their specific descriptions in the data sources section. The relevant textual descriptions are included in the content below Table 1. If there are any other requirements, we will do our utmost to provide additional information.

**Minor Comment 29**

L557-563: I don’t know if you can conclude any of this based on your study. You don’t look into population data or emissions as predictor variables and GPP has little influence on your model. And the same with coal heating. I would rewrite this to say that these may be causing the spatial clustering and cite studies that have found this in the past.

**Response:** The acquisition of human factor data is indeed quite challenging, and this problem remains unsolved due to issues related to the age of the data and data integrity. After careful consideration and incorporating previous discussions, we have made deletions to the content concerning emission sources and human factors. Meanwhile, we have streamlined the text to ensure clarity and coherence while retaining key information.

**Concluding Response：**Thank you for your valuable comments on our manuscript. We have carefully considered both the major and minor issues you raised. Regarding the major issues, we have made every effort to improve the content, and provided explanations for the parts that could not be modified. With regard to the minor issues, we appreciate your thorough review. Some of the errors identified were due to our oversight, and we have carefully checked the manuscript to prevent unnecessary mistakes from occurring. Additionally, we have carefully revised some of the issues and marked their positions; please review them again.

**Authors’ Response to Reviewer 2**

**Major Comment 1**

The authors do not detail the influence of emission sources and atmospheric chemical interactions of pollutants emitted from these urban emission sources and atmospheric chemical interactions of pollutants emitted from these urban centers, which are widely documented as the main factors driving the behavior of tropospheric centers, which are widely documented as the main factors driving the behavior of tropospheric NO_2_.

**Response:** Thank you for your feedback. Regarding this issue, I would like to explain as follows: The current research mainly focuses on the temporal and spatial distribution of nitrogen dioxide pollutants and their influencing factors. The photochemical reactions and secondary transformations that occur after pollutants enter the atmosphere (such as the formation mechanism of PM2.5, the interaction of ozone precursors, etc.), as well as the synergistic/antagonistic effects between different pollutants, have provided us with new ideas and may become the focus of our next research. For this article, we will supplement the research of relevant literature in the introduction section. Thank you for your professional suggestions, which are very important to us. (L43)

**Major Comment 2**

Differentiation of NO_2_ behavior according to regional climatic characteristics is proposed, however, it is not described or discussed in the document. Therefore, it is advisable to specify the research objective in such a way that it emphasizes the evaluation of the spatiotemporal behavior of NO_2_, particularly in relation to climatic factors and their relationship with land cover. Or, conversely, to rethink and expand the research, at least on the role of emission sources (by source type) and to delve deeper into the analysis of the specific weight of the country's climatic diversity.

**Response:** Thank you for your feedback. Incorporating climate zones can indeed facilitate a better discussion on the impact of meteorological factors on NO_2_ pollutant concentrations. In this regard, we have redrawn the figures and conducted an analysis in combination with climate zones. This can be found in Section Figer-6 of the article.

**Major Comment 3**

In this sense, it appears to be a relevant and well-implemented method for assessing climate, land cover, land cover-atmosphere interactions, and temporal factors; however, its potential to include additional factors such as predominant emission sources (mobile or stationary sources), as well as the interactions of NO_2_ with other pollutants such as PM_2.5_ or O_3_ itself in the troposphere, has been underutilized.

**Response:** Thank you for your feedback. Regarding other factors, such as major emission sources (mobile or stationary), during the data preparation phase, we did include population, land use, and road systems in our consideration as influencing factors. However, the data were less than ideal in terms of their temporal scale and completeness. Among these, urban environmental factors, presented in the study in the form of urban boundary vectors, can be regarded as anthropogenic factors but were not included in the ranking within the random forest model.

As for your reference to the interactions between NO_2_ and other pollutants (such as PM_2.5_ in the troposphere or O_3_ itself), due to the increased variety of pollutants, the volume of data to be acquired would be substantial, accompanied by a significant workload. Therefore, this aspect was not discussed. We have carefully reflected on the professional questions you raised and supplemented relevant literature in Introduction. Thank you.

**Major Comment 4**

The information sources for NO_2_ (SP5), MODIS, and ECMWF/ERA5, along with their inherent uncertainty, were not described in detail. Therefore, questions remain about the impact of source uncertainty and its role in the assessment process, as well as about the response of the prediction model. The model is also not validated with information derived from local grid monitoring sources, or at least from estimates such as emissions inventories.

**Response:** Thank you for your feedback. Explanations regarding the data sources of NO_2_ (SP5), MODIS, and ECMWF/ERA5 have been supplemented in L126-L156. As for the issue of data validation, we once considered using emission inventories for verification. However, our research period ends in 2023, while the complete data currently available only goes up to 2020. To address the validation problem, we adopted the approach of dividing the data into a training set and a validation set, with two-thirds of the data used for training and one-third for validation.

**Minor Comment 1**

It is important to explain why the seasonal analysis of the data was chosen instead of monthly. This provides greater temporal resolution for the time series analysis regarding trends and variability.

**Response:** Thank you for the comment. The issue you raised has been considered by us before. We ultimately chose to analyze seasonal averages rather than monthly data for the following reasons: The concentration of NO_2_ is jointly influenced by natural meteorological conditions (such as air pressure, temperature, and precipitation) and anthropogenic factors, and its fluctuations usually exhibit significant quarterly periodicity, making seasonal data more researchable; high spatiotemporal resolution data may have local missing values at the monthly scale due to factors like cloud cover(Yichen Li, et al. 2025); air pollution control policies (such as the "Autumn and Winter Campaign") are usually formulated on a seasonal basis, so the results of seasonal analysis can directly correspond to the policy cycle. Thank you again for raising this question. The above are the reasons for our choice of seasonal data.

**Minor Comment 2**

Nothing is mentioned or highlighted about the effect of hydroelectric plants or forest fires, much less the emission sources, which are primarily related to tropospheric NO_2_ air pollution. The use of Sentinel offers such spatial resolution that these emission sources can be spatially delimited and their regional influence highlighted.

**Response:** Thank you for the comment. Hydropower plants are classified as human factors, and since human factor-related indicators were not selected in this study, no analysis will be conducted on this aspect. Regarding the factor of forest fires, the study has chosen a significant product from the MOD14A1 fire detection and thermal anomaly dataset. Since fire does not rank among the top five in the random forest importance ranking, we will only include a brief analysis of it in the discussion section. The specific content will be added in L526.

**Minor Comment 3**

It should be regard the uncertainty inherent in the data source (Sentinel-5).

**Response:** Thank you for the comment. It is true that SP5 has inherent uncertainties in its data sources. For example, cloud cover can block signals from the Earth's surface, and aerosol scattering can lead to inversion errors; data noise increases significantly in mountainous or polar regions; and there are outliers in the data(Miriam Latsch, et al.2022). However, the data selected in this study is limited to urban environments, and the outliers are within a controllable range. Therefore, the issues with SP5 have little impact on this research. Thank you again for your professional comments.

**Minor Comment 4**

It is suggested that complementary sources of information, such as monitoring systems or national pollutant inventories, be consulted to corroborate and discuss the marked seasonal and spatial contrasts.（L481, L497, et.）

**Response:** Thank you for the comment. It remains challenging to obtain the latest emission data of China's prefecture-level cities due to the lack of official disclosure (Kurokawa & Ohara, 2020). The latest data we can access is as of 2020, which does not match our time scale. We agree that public emission inventories would be of great significance to this research. However, for the current study, we have adopted the method of comparing it with the research of other scholars to enhance the credibility of this conten.

**Punctual observations**

**Title 1**

In reality, this work does not address the dynamic behavior of atmospheric NO_2_ chemistry, but rather an analysis of its transient behavior on a monthly or seasonal scale.

**Response:** Thank you for the reviewer's pointing out this important distinction. It is true that this study focuses on the characteristics of NO_2_ changes over a relatively large time scale rather than dynamic changes. We agree that "dynamic behavior" is more applicable to chemical processes on short time scales, so in the revised manuscript, the title of the article has been changed to "Nitrogen dioxide pollution in 346 Chinese Cities: Spatiotemporal variations and drivers from multi-source remote sensing data".

**Title 2**

This is important to consider, both in the title and in the conceptual basis that underpins this research, whether for correcting or presenting results and developing the discussion around the physical and chemical phenomena that govern the mechanisms and reactions in the troposphere, the products and sub-products resulting from its interaction with specific environmental conditions, as well as its relationship with emission sources.

**Response:** Thank you for your questions and suggestions. We fully agree with what you have said, but our research is based on remote sensing data rather than being in the fields of chemistry or meteorology. The factors we selected are more inclined to those accessible through remote sensing methods, such as temperature, evapotranspiration, and air pressure. The analysis of the interaction mechanisms between different layers of the atmosphere is challenging. In terms of data acquisition, we can only obtain numerical values and conduct simple analyses on them (as shown in Figure 10). This study mainly focuses on exploring the causes of changes in NO_2_ concentration using remote sensing data. Although it has limitations, it can cover the entire China, with a relatively wide scope. Thank you again for your professional suggestions, which have provided new ideas for our future research.

**Abstract**

**Response:** We appreciate the reviewer's constructive feedback on our abstract. In response, we have made the following revisions:

(1) The content after "such as" has been revised to: "the most prominent are atmospheric pressure, aerosol optical depth, Leaf Area Index, evapotranspiration, and dew point temperature."

(2) Regarding "partial dependence plots...", the language has been reorganized to: "A non-linear response curve methodology was employed to analyse the independent association characteristics between driving factors and pollutant concentrations."

(3) After careful consideration of the part you suggested deleting, we recognize that it was indeed an oversight in our language description, and we have revised it in accordance with your requirements.

**Abstract 1**

Few lines relate to the methodology efficiency would reinforce the abstract.

**Response:** Thank you for the comment. We sincerely appreciate the reviewer's valuable suggestion regarding methodological efficiency. In response, we have strengthened the abstract by:

To further demonstrate the model's reliability, we have included R² evaluation metrics in the abstract to assess the algorithm's robustness. (L20-L22)

**Abstract 2**

In four years, is difficult establishing a trend pattern with specific breakpoints... It should be managed as interannual variation linked to a specific weather proxy. This temporal variation is similar over all the cities? It should be significant differences by region!

**Response:** We wholeheartedly accept the proposed revisions. Establishing definitive breakpoints in the 2019-2023 trend pattern does indeed present considerable methodological challenges. In accordance with your recommendations, we have (L27-L28):

(1)Completely removed the breakpoint analysis

(2)Adopted the more appropriate descriptor of "fluctuating characteristics"

(3)Shifted focus to highlight significant inter-regional variations

**Introduction 1**

Please indicate what this data are and their sources of information! Is this a reference? It's not included in the reference list!

**Response:** Thank you for pointing out the problem. We carefully considered this passage and found that it can be deleted without affecting the meaning expressed in the article, and at the same time, it makes the article look more concise.

**Materials and Methods 1**

These can easily be separated into two subheadings to address them separately and go into more detail if needed!

**Response:** Thank you for the comment. We fully agree with your suggestions. We have separated the study area and data sources into two sections for discussion, with the specific content included in the "Study area" and "Data sources" sections of the article. (L81, L105)

**Materials and Methods 2**

This term is uncommon! Could it be a subarctic region?

**Response:** Thank you for the comment. The study area features diverse climate types. What we intended to express was the cold zone. Due to our poor language expression, this error has been corrected in Line 85 as per your request.

**Materials and Methods 3**

Sorry but I didn't understand what you mean!

**Response:** Thank you for the comment. This part was added here due to an oversight. Thank you for your careful review. We have removed the content irrelevant to the article.

**Materials and Methods 4**

It is important to describe: 1) The data source and the factors that generate uncertainty in the Sentinel 5P product, 2) The time window of the source data for estimating seasonal and annual averages, 3) Its characteristics: level product, spectral regions, pixel size, local time measurement, standard unit of measurement (even more so if tropospheric NO_2_ is presented in this work with a different numerical scale), 4) Accuracy estimates, 5) Quality assurance value (qa_value) for interpreting data quality (e.g., pixel qe_value greater than 0.75 or 0.5 for cloudy scenes) and, 6) Advantages and disadvantages.

**Response:** Thank you for your careful review. I will rewrite the data source section in accordance with your suggestions, and the specific content can be found in Line117-L127.

**Materials and Methods 5**

Assuming we are talking specifically about tropospheric NO_2_... For example, it does not make any difference whether they are using vertical column density measurements, taking into account the differences between NO_2_ (slanted column density) vs. NO_2_ (vertical column density), or stratospheric vertical column vs. tropospheric vertical column.

**Response:** Thank you for your professional comments. Regarding this issue, let me explain. The NO_2_ concentrations obtained in this study are all tropospheric vertical column concentrations.

**Materials and Methods 6**

It is worth highlighting why it is better to use Sentinel-5P Offline L3 NO_2_ instead of Sentinel-5P Near Real-Time NO_2_ or Reprocessed (RPRO-NO_2_), as well as their advantages and disadvantages.

**Response:** Thank you for the comment. The specific advantages and disadvantages have been explained in the Data sources section of the article, on Line 123.

**Materials and Methods 7**

Which band(s) were used for the analysis? Explain your choice.

**Response:** Thank you for the comment. This dataset includes:

- Optical_Depth_Land_And_Ocean: Aerosol Optical Depth (AOD), covering both land and ocean areas.

- Effective_Radius_Land: Effective radius of aerosols in land regions.

- Effective_Radius_Ocean: Effective radius of aerosols in ocean regions.

- Angstrom_Exponent_Land: Angstrom exponent, indicating the size distribution of aerosols.

- Quality_Assurance_Land: Data quality assurance indicator, used to evaluate the reliability of the data.

In this study, Optical_Depth_Land_And_Ocean is selected for research.

**Materials and Methods 8**

Why did you found the centroid? Please, it is necessary conclude this idea.

**Response:** Thank you for the comment. For irregularly shaped built-up areas, the centroid can better represent the "balanced position" of spatial distribution than a simple geometric center. The advantage of the centroid lies in its ability to avoid edge errors, making it relatively convenient for statistics and modeling. Other options, such as the geometric center, may fall outside the built-up area and thus fail to represent the urban environment; while the population-weighted center requires high-precision population data, which is relatively difficult to obtain (([Paul A. Longley](https://www.wiley.com/en-cn/search?filters%5bauthor%5d=Paul%20A.%20Longley&pq=++),et al.2015).

**Materials and Methods 9**

It's necessary to follow the journal's hierarchical format; it's confusing. It gives the impression, and it seems, that it's the methodological description, but it doesn't seem to be organized in a hierarchy according to the journal's format. I mention this because it could cause confusion if the hierarchical order of a section heading isn't clearly defined.

**Response:** We sincerely appreciate the reviewer's guidance on improving the manuscript's structure. First, we have clarified that title levels include fonts and sizes. Secondly, due to issues with our language expression, I request to explain the structure of this part again. Data preprocessing is a data processing method used in this research, and its content is divided into two parts: remote sensing data preprocessing, Sentinel-5P data processing and Data collation（[Thomas Lillesand](https://www.wiley.com/en-us/search?filters%5bauthor%5d=Thomas%20Lillesand&pq=++), et al.2015）.Since only after the data is processed can the subsequent series of spatiotemporal analyses and exploration of influencing factors be conducted, we have placed data preprocessing as the first point in the methodology. In addition, we have improved the data preprocessing section, dividing the process into three parts: Remote sensing data processing, Sentinel-5P series, and Sentinel-5P series. (L169-L174)

**Materials and Methods 10**

Explain this idea in more detail. Clarify that it's a limitation for spatial and temporal analysis, but from there, move on to the next sentence with the problem solved. It's not clear if or how you processed that data.

**Response:** Thank you for the comment. Let me explain this: GEE uses the harpconvert tool to convert L2 data into L3 data classified by latitude and longitude, filters out pixels with low quality, and generates corresponding datasets. This study directly adopts L3-level products for analysis, and L3-level products have the following advantages: they have undergone strict quality control and spatial standardization; they feature a unified spatiotemporal resolution and coordinate reference system; they avoid the issue of spatiotemporal inconsistency existing in the original L2 data; and they are more suitable for environmental monitoring research in large-scale regions (Takashi Sekiya, et al.2022).

**Materials and Methods 11**

What attributes are evaluated? The Sentinel-5P series or the parameters in Table 1? Is the analysis performed layer by layer, i.e., individually?

**Response:** Thank you for the comment. Due to inaccuracies in our expression, there has been a deviation in the intended meaning. The average value of the attribute refers to the average of the Sentinel-5P series data. Since the local spatial autocorrelation analysis focuses on NO_2_ concentrations over a five-year period, the average value of the attribute here specifically refers to the annual average of NO_2_ concentrations. （L194）

**Materials and Methods 12**

Regardless of whether cluster analysis is applied to the Sentinel series or to the data layers in Table 1, it is important to consider whether the mean, median of a fixed time scale (e.g., monthly average), or snapshot (image/band) is being evaluated.

**Response:** We appreciate the reviewer's attention to data presentation details. In Figures 3-5:All figures display mean NO_2_ concentrations as the primary metric, representing overall pollution levels.Figures3&4 additionally annotate the median values (as horizontal dashed lines) to: Highlight central tendencies unaffected by extreme values Figure 5 focuses on spatial patterns where means suffice for inter-region comparisons. Meanwhile, we have enhanced the visualization of NO_2_ concentrations with the Hu Huanyong Line as an auxiliary line (shown in Figure 1).

**Materials and Methods 13**

Please refer to the corresponding equation.

**Response:** Thank you for the comment. We rigorously verified the formula and its representative values, and incorporated the revised version into the SHAP methodology section of our Methods.

**Materials and Methods 14**

Did you mean: clusters?

**Response:** Thank you for the comment. Due to potential ambiguities in our initial language, we would like to clarify the concept of 'clustering' in local spatial autocorrelation analysis. Unlike general 'concentration distributions,' clustering emphasizes both statistical significance and spatial topological relationships (requiring analysis with a distance-based weight matrix) ( JK Ord, A Getis.1995). In this study, we employed Geoda software to conduct local spatial autocorrelation analysis of NO_2_ concentrations across 346 Chinese cities, identifying the spatial heterogeneity of NO_2_ concentrations among different clustering types (e.g., High-High, Low-Low). By distinguishing these clustering types, we can formulate spatially differentiated policies with precision, avoiding a "one-size-fits-all" governance approach.

**Results 1**

It could be suppressed!

**Response:** Thank you for your valuable suggestion. We have revised the subheadings in Sections 3.1 and 3.2 to make them more concise, as recommended. Section 3.1 has been revised to "Temporal distribution of NO_2_ concentration", and Section 3.2 to "Spatial distribution characteristics of NO_2_ concentration".（L282, L322）

**Results 2**

This fits into the methodology section.

**Response:** Thank you for the comment. As suggested, the detailed description of ANOVA implementation has been moved from Section 3.1 to Section 2.3 Methods (Lines 179-L184).

**Results 3**

This is already summarized in Table 2. It is suggested to delete it.

**Response:** We appreciate the reviewer's careful reading. As suggested, the redundant text has been deleted from Section 3.1. The complete data are now exclusively presented in Table 2 for conciseness. All subsequent discussions have been updated to reference Table 2 accordingly.

**Results 4**

This is an interpretation that should be moved and discussed in the discussion section!

**Response:** Thank you for the comment. We sincerely appreciate your valuable suggestion. As recommended, the discussion regarding "the underlying mechanisms of seasonal NO_2_ concentration variations" has been systematically relocated to Section 4 (Discussion) (see revised manuscript, Lines 494–499). The following improvements have been implemented:

(1) Enhanced mechanistic analysis of NO_2_ drivers.
(2) Streamlined Results (Section 3.1) to focus exclusively on objective data presentation.

**Results 5**

It is necessary to include graphic information or a reference about this argument!

**Response:** Thank you for the comment. We appreciate your comment regarding the explanation of outliers in Figure 5. Upon careful consideration, we have removed the statement discussing "why winter shows more high outliers" for the following reasons:

**Results 6**

Annual NO_2_ autocorrelation maps would help highlight clusters with their own degree of significance, rather than Figures 5a-e.

**Response:** Thank you for your constructive comments. Regarding the interannual variation of NO_2_ concentrations in Figure 5, we have implemented the following improvements:

(1)Climate-zone based analysis: We have incorporated climate zone classification into our analysis of urban NO_2_ concentration trends, as climate zones are closely associated with meteorological factors and provide valuable context for interpreting the results.

(3)For the local spatial autocorrelation analysis, we will utilize these results to discuss the characteristics of NO_2_ concentration variations among different clustering types (e.g., High-High and Low-Low clusters).

These modifications have been carefully implemented in the revised manuscript (see L510-L516). We believe these changes significantly strengthen our analysis and presentation.

**Results 7**

This line is not defined on the maps, nor is it previously mentioned!

**Response:** Thank you for your question regarding the noun not mentioned in the article. We have added an explanation of this below the research area section and included this element in the research area map. The differences in climatic conditions, intensity of human activities, and pollution emission patterns between the east and west sides of this line make it an important reference framework for studying the pattern of air pollution in China.

**Results 8**

There is no map that delineates these natural features (geomorphological, rivers, or deltas), climatic regions, watersheds, or specific elements such as cities that stand out as highly polluted urban centers.

**Response:** Thank you for the comment. We sincerely appreciate this insightful observation. Due to the issues in our language expression, I will re-explain and revise it for you (L382-L384):

Since inherent nouns are not easy to understand, and adding various geographical element maps would take up a lot of space, we have unified and sorted out these regions, and described them by listing the names of each city. I believe our approach will help in understanding this part of the content.

**Results 9**

Units should be indicated in the caption of this image. A multi-color scale would help highlight cities or regions with critical concentrations, rather than urban density. This is an interpretation that should be moved and discussed in the discussion section!

**Response:** Thank you for your professional feedback on our figures. The absence of units in the figure was an oversight on our part. We have revised the figure 6 by incorporating climate zones, and have added the units for NO_2_ concentration. Please kindly review Figure 5 again.

**Results 10**

This cluster classification, combined with the location of the cities and their relationship to the climatic region, would provide elements for discussion on the influence of this factor, which was highlighted at the beginning of the study area section.

**Response:** Thank you for raising this important professional concern regarding the climate zones. We fully acknowledge the significance of this issue and, as mentioned in our earlier response, have incorporated climate zone considerations into our discussion. Please refer to Section L510-L516 for the additional details addressing this suggestion.

**Results 11**

Is it necessary to explain what this refers to?

**Response:** Thank you for pointing out the confusion. We apologize for the ambiguity in our expression. We have explained in detail the unique geographical location of Yulin City and appropriately incorporated it into lines 360 - 363. We hope this explanation clarifies things for you. Please feel free to let us know if you have any further questions.

**Results 12**

This is unclear! Furthermore, there are no references to support this type of argument.

**Response:** Thank you for the comment. After careful evaluation, we believe that this content has a weak connection with the analysis in the Results section, so this part has been deleted. The subsequent content has been adjusted accordingly to ensure logical consistency.

**Results 13**

The inset in this image appears to show a larger (zoomed-out) region not shown on the map. The "changed many times" class is indistinguishable from the "No significant" class; please change the dot color or font. Note on formatting this figure type (map). If presented as a map, please note the following: 1) Incorporate the reference system and map projection information, 2) Add a grid or marks to the effective map area, 3) Complete the coordinate information in the lower right ribbon.

**Response:** Thank you for your professional advice on our graphs. Regarding the color ribbon selection, we uniformly adopted a blue-purple series. However, due to the low resolution when converting PDF to images, the visualization effect is not ideal. We have attached the original image below for your reference, where "cities with significant changes" are marked in light purple and "cities with insignificant changes" in gray.

As for the grid issue, since all images involving China maps in our entire article use grids of the same format, this is considered more reasonable for the overall reading experience.

Thank you again for your professional evaluation of the graphics, which has provided us with new ideas for drawing graphs in future research.

**Results 14**

Unless this line of thinking is part of the partial conclusions drawn from a detailed analysis of seasonal variability... It is difficult to assume these relationships without supporting data. No correlation analysis or multifactorial comparison, to mention a few examples, is presented that would allow us to understand or assume this causal relationship.

**Response:** We fully agree with your view. In the revised manuscript, we have supplemented relevant literature (e.g., [54-56]), with a focus on elaborating the impact of unstable meteorological conditions (such as changes in wind speed and boundary layer height) on fluctuations in pollutant concentrations.If you consider that additional data or analyses are still needed, we are more than willing to further improve the manuscript in accordance with your suggestions. Once again, thank you for your rigorous and constructive comments, which are crucial for enhancing the quality of the paper.

**Results 15**

This subheading is very long, please work on summarizing it and the subheadings for sections 3.1 and 3.2.

**Response:** Thank you for your suggestion. We have revised the subheading to make it more concise as follows:

"Factors influencing tropospheric NO_2_ in Chinese cities." We appreciate your valuable feedback, which has helped improve the clarity of our manuscript. （L404）

**Results 16**

At this point in the document, there is no evidence of NO_2_ measurements from monitoring systems or pollutant inventories that corroborate or validate the model's results. If the training and validation process were conducted using the satellite data itself, the inherent uncertainty of the Sentinel 5P product with respect to its spatiotemporal analysis becomes relevant.

**Response:** Thank you for the comment. It is true that SP5 has inherent uncertainties in its data sources (Yuhang Zhang, et al.2025; Jos van Geffen, et al.2022). However, the data selected in this study is limited to urban environments, and the outliers are within a controllable range. Therefore, the issues with SP5 have little impact on this research. Thank you again for your professional comments.

**Results 17**

What are these observed values? They do not refer to any data source or local NO_2_ monitoring system. They are not previously mentioned, nor are data or access links presented.

**Response:** Thank you for your high-level question. Due to the ambiguity in our expression, which has caused your confusion, we will revise this sentence to "The results showed that the slopes of the modeling group and the validation group were 0.86 and 0.74, respectively, with R² values of 0.94 and 0.76. The data points of both the modeling group and the validation group were close to the 1:1 fitting line, as shown in Figure 3-6, indicating that the obtained RF model has a high degree of fitting." The specific details are presented above Figure 9. （L412-L416）

**Results 18**

On the “Y” axis, what is the validation data source?

**Response:** Thank you for the comment. The Y-axis validation data source was Sentinel-5P data. While the training and validation processes utilized the satellite data itself - acknowledging the inherent uncertainties of Sentinel-5P products in spatiotemporal analysis - these uncertainty factors have been explicitly addressed in Results 16. We sincerely appreciate your expert commentary on this matter.

**Results 19**

It is not necessary here, this information is in Figure 9. Perhaps the most important factors could be mentioned here.

**Response:** Thank you for the comment. Thank you for highlighting this issue. We fully accept and have incorporated your suggestion by removing the mentioned sentence for two reasons:

The content is already clearly presented in Figure 10, and textual repetition would make the manuscript unnecessarily redundant.

The most important factors are subsequently discussed in detail, including explicit mention of the top five contributing factors.

This deletion enhances the manuscript's conciseness without compromising key information. We truly appreciate your professional insights that have helped improve our paper's quality.

**Results 20**

Since remote sensing of atmospheric gases involves the study of complex masses of substances in a dynamic environment, the estimation of a gas such as NO_2_ is subject to a wide variety of phenomena and factors that influence its spectral response. In this regard, it is necessary to assess whether any byproducts (e.g., real-time snow and ice extent, wind direction, surface pressure, vapor pressure, albedo) from other satellite platforms (e.g., MODIS) or models (GEOS-5) favor the collinearity of some variables identified in this study with the Sentinel-5P Offline L3 NO_2_ satellite measurements.

**Response:** Thank you for your professional comments. We have considered this issue before, but since it is not the focus of our research and a detailed study would take up too much space, we would like to explain it to you as follows:

1. This study focuses on Sentinel-5P NO_2_ data, and other variables (such as snow and ice cover) have limited impact on our research objectives.

2. It is emphasized that the NO_2_ retrieval algorithm of Sentinel-5P has taken key interfering factors (such as aerosols, clouds, etc.) into account. As mentioned above, we discuss years and seasons, although the interfering factors have a greater impact on monthly data when acquiring such data. (S5P-KNMI-L2-0005-RP)

3. All data in this study were obtained from the GEE platform, whose temporal and spatial resolutions match those of the Sentinel-5P data, thus reducing additional data uncertainties.

4. This research focuses on the Sentinel-5P data itself rather than multi-source data fusion, aiming to explore the spatiotemporal distribution of NO_2_ concentrations and their influencing factors. However, in future work, we will consider the multi-data collaborative analysis you mentioned, which also provides new ideas for our work.

Thank you again for your professional suggestions.

**Results 21**

It is interesting to see the relationship between variables and how they play a greater role in predicting NO_2_ concentrations. However, since prediction models based on machine learning or deep learning are codependent on the volume of data and their own analytical and predictive capacity, a physical or chemical explanation is necessary to broaden the discussion regarding for instance, the climatological gradient according to the region in which a city or cluster of cities is located.

**Response:** We sincerely appreciate your insightful and constructive feedback, and we fully agree with it. We will analyze the climatic zone characteristics of different urban agglomerations. The spatial patterns have been visually presented in Figure 6 of the Results section.

**Results 22**

What was the criterion for choosing between AOD and wind? Why were both not included in the analysis and discussion?

**Response:** Thank you for the comment. We sincerely appreciate this important question regarding the selection criteria for AOD and wind speed variables. Our considerations are as follows:

Variable Selection Criteria:

Primary screening was based on Random Forest feature importance ranking (top one-third of variables). Other variables showing strong correlation with these top predictors received secondary analysis.

While AOD ranked within the top one-third of important variables, it was ultimately excluded due to poor temporal continuity caused by cloud cover.

**Results 23**

Please review the measurement system in this and the other figures to ensure it conforms to conventional expressions. For example, atmospheric pressure is commonly expressed in hPa or millibars.

**Response:** Thank you for the comment. Thank you for your thoughtful comment regarding the units of measurement. We sincerely appreciate your attention to this technical detail. The conversion from hectopascals (hPa) to pascals (Pa) was based on the following considerations:

Data source consistency: The original datasets we acquired were uniformly provided in Pa units by the data providers (C.O Justice, et al.2002).

Dimensional harmony: Pascal units (Pa) maintain better consistency with other atmospheric parameters in our analysis, particularly in subsequent physicochemical calculations.

**Results 24**

It is necessary to label the SHAP value units, a brief description in the figure caption, and in the body of the document, indicate what it means for each of the variables presented (if the value is different between variables).

**Response:** Thank you for the comment. We sincerely appreciate your expert guidance regarding the clarification of SHAP value units. We have implemented the following improvements:

The units of SHAP values represent the probability change amount.

We have added the following annotation below Figure 11:"SHAP values indicate the absolute effect size of features on the target variable, where the magnitude directly reflects each feature's marginal contribution to NO_2_ concentration variations."（L452-L454）

**Discussion 1**

I understand that the RF model identifies the weight of influence based on the factors or variables that one includes in the modeling process, therefore the identification of key factors is subject to our discretion and not to the analytical capacity of the model.

**Response:** Thank you for the comment. We sincerely appreciate your valuable suggestion. Upon careful consideration, we agree that the discussion of "model analytical capability" was not appropriate in this context.

We have therefore removed the specified passage while ensuring:

1.The core meaning of the original text remains intact

2.All key information is preserved in adjacent sections

3.The revised version achieves greater conciseness

We are grateful for your expert feedback, which has significantly improved the manuscript's clarity.

**Discussion 2**

Please review the color scale, it is incomplete and does not seem to correspond to the classification in the matrix.

**Response:** Thank you for your valuable feedback on the color bar in our figures. Your meticulous attention to detail has greatly enhanced the quality of our manuscript.

We have carefully revised the issue with the color bar, and the updated version is presented as Figure 12 in the revised manuscript. Your professional suggestions have significantly improved the clarity and professionalism of our data visualization.

**Discussion 3**

Why was the coefficient of determination not chosen?

**Response:** Thank you for your insightful comment regarding the use of correlation analysis. In response to your suggestion, we have refined our approach as follows:

Terminology Clarification:

We have replaced "correlation coefficients" with the broader term "correlation analysis" to more accurately reflect our methodology. ( The second paragraph of the discussion section)

Visual Demonstration:

The correlation patterns are now presented via a correlation heatmap (see new Fig. 12), which provides:

The revised analysis appears in Section Discussion. We believe this modification strengthens our results' validity while addressing your constructive feedback.

**Discussion 4**

A value lower than or close to 0.7 is considered moderate or weak. Why did you treat these values as strong correlations? Under what criteria are they considered strong correlations?

**Response:** Thank you for raising this important methodological question. Our interpretation of correlation strength was based on domain-specific criteria, where a threshold of 0.65 aligns with recent NO_2_ satellite retrieval studies (Liu et al., 2023, Remote Sensing). To better support this, we have now explicitly cited this reference in the correlation analysis section. ( The second paragraph of the discussion section)

We sincerely appreciate your expert guidance, which has helped strengthen our methodological rigor. Please let us know if further clarification would be helpful. (L472-L474)

**Discussion 6**

Please be clear and concise. This is a correlation matrix.

**Response:** Thank you for your valuable suggestions regarding the thermodynamic diagram. We agree that the original figure title was unnecessarily complex. Following your guidance, we have streamlined it, and the revised version is now presented as Figure 11 in the updated manuscript.

Your expert advice has significantly enhanced both the rigor and clarity of our study. We sincerely appreciate your contribution to improving this work.( L477“Thermodynamic diagram of NO_2_ influencing factors”)

**Discussion 5**

Seasonal change is directly related to meteorological factors and their interactions (available light-temperature-humidity), and to atmospheric pressure at the synoptic scale. Given that these seasonal changes strongly influence changes in NO_2_ concentrations, why don't these environmental factors appear at the top of the list in the SHAP analysis? How do you explain such relationships?

**Response:** Thank you for your professional advice. Let me explain the reason behind this issue: In the SHAP importance ranking, although meteorological factors such as temperature and humidity are ranked in the middle, it does not mean they have no relationship with the seasonal variation of NO_2_ concentration. It is just that their influence is weaker compared to factors like air pressure and evapotranspiration. Therefore, while discussing and analyzing the top five important factors, we also incorporate other meteorological factors (such as air temperature, humidity, and dew point temperature) into our analysis. The point you raised has prompted us to present the role of meteorological factors more comprehensively, which is crucial for improving the mechanism discussion in the paper.

**Discussion 6**

This is not clearly noticeable!

**Response:** Thank you for the comment. We acknowledge that our original phrasing could have been more precise. In response to your comments, we have revised the text by:

1. Replacing "interannual trend variations" with "wave-like fluctuations"

2. Removing potentially ambiguous terms like "peaks"

**Discussion 7**

The literature widely suggests that emission sources, atmospheric chemical reactions, and meteorological processes are the main factors controlling NO_2_ concentrations in the troposphere. It is surprising that the LAI appears as one of the main factors, so I believe its importance should be emphasized in the manuscript's statement, discussion, and conclusions.

**Response:** Thank you for the comment. We sincerely appreciate your valuable suggestions and have carefully considered each point. Please allow us to address your concerns in detail:

Emission Sources:

Primary sources (vehicle exhaust, industrial emissions, etc.) pose challenges for data collection due to:

Limited temporal coverage (data unavailable after 2020)

Requirements for massive amounts of data

We acknowledge that these anthropogenic factors require further investigation in future studies.

Atmospheric Interactions:

While analyzing the interactions between O_3_, PM_2.5_, and CO would be valuable:

Such analysis would significantly expand the scope of the research

Our current focus remains on remote sensing applications for atmospheric pollution

Meteorological Processes:

We fully accept this suggestion and have conducted a comprehensive literature review, which is specifically included in the discussion section.

These improvements are present in the revised manuscript (track changes mode). We are grateful for your expert guidance, which has greatly enhanced the quality of this work. If you recommend further improvements, we will be happy to implement them.

**Concluding Response:** Thank you for your valuable comments on our manuscript. We have carefully considered both the major and minor issues you pointed out. Regarding the major issues, we have made every effort to improve the relevant content and provided explanations for the parts that could not be modified. As for the minor issues, we have carefully responded to and revised them in accordance with the questions and suggestions you put forward in each section. We kindly request you to review the manuscript again. The suggestions you provided have been of great help in improving the quality of our research.
