# Supplementary material for: Nitrogen dioxide pollution in 346 Chinese cities: Spatiotemporal variations and natural drivers from multi-source remote sensing data
Source: PLoS One. 2025 Nov 7;20(11):e0334535. doi: 10.1371/journal.pone.0334535 (PMC12594365; doi:10.1371/journal.pone.0334535)
Supplement: Attachment — Submitted filename: Response_to_Reviewers_auresp_2.docx [file pone.0334535.s009.docx]

Responses to Reviewers’ Comments for Manuscript PONE-D-25-30016R1

**Nitrogen dioxide pollution in 346 Chinese Cities: Spatiotemporal variations and drivers from multi-source remote sensing data**

Addressed Comments for Publication to

PLOS ONE

by

Heming Yang

Dear Dr. Editor,

We greatly appreciate your kind letter and positive feedback, which have been a great source of encouragement for us. We will definitely take each comment from the review experts seriously, revise the manuscript meticulously, and strive for perfection. We look forward to the smooth publication of the manuscript under your guidance.

Please find enclosed the revised version of our previous submission entitled “Nitrogen dioxide pollution in 346 Chinese Cities: Spatiotemporal variations and drivers from multi-source remote sensing data” with manuscript number PONE-D-25-30016R1. We would like to thank you and the reviewers for the valuable comments which help improving the quality of our manuscript.

In this revision, we have carefully addressed the reviewers’ comments. A summary of main modifications and a detailed point-by-point response to the comments from Reviewers 1 to 2 are given below.

Sincerely,

Heming Yang

**Note:** To improve the readability of the response letter, the editor's comments are replied to in red text, while the comments from the two reviewers are addressed point by point in blue text and green text respectively..

**Authors’ Response to the Editor**

**(1)** **Please ensure that you refer to Figure 1,2,7 in your text as, if accepted, production will need this reference to link the reader to the figure.**

**Response:** Thank you for your reminder. We have promptly checked the manuscript and added citations for Figures 1, 2, and 7 to meet your requirements. These citations are marked in red font at Lines 90, 271, 399, and 416 of the "Revised Manuscript with Track Changes".

**(2)If the reviewer comments include a recommendation to cite specific previously published works, please review and evaluate these publications to determine whether they are relevant and should be cited. There is no requirement to cite these works unless the editor has indicated otherwise.**

**Response:** Your opinion on "not citing literature blindly" is very insightful. After re-examining the manuscript, I realize that some of the literature citations do have the issue of being included merely for the sake of citation, with insufficient connection to the argumentation in the context. My original intention was to present the relevant research background as comprehensively as possible, but the approach indeed needs improvement. Moving forward, I will make revisions from the following aspects:

Check each citation in the text one by one to ensure that it directly supports or serves the core argument of the paragraph.

Delete those literature sources that have weak relevance and are only used to inflate the citation count, so as to achieve the goal of making citations necessary and accurate.

**(3)In particular, Reviewer 1 emphasizes the problem of exclusion bias arising from the lack of anthropogenic variables in your models, noting that the omission of key NO₂ sources in urban environments (e.g., road networks) may distort the importance of meteorological and vegetation predictors. While impervious surface area partly captures built environment, some proxy for road density or major highways should be included to strengthen the analysis and avoid misrepresentation of pollution drivers.**

**Response:** Thank you very much for your valuable suggestions. Your proposal to introduce the road density factor to improve the model is a highly insightful perspective, and it is indeed of great importance for capturing the spatial distribution pattern of pollutants.

After carefully considering your suggestion, we also conducted in-depth discussions on such static spatial factors during the initial stage of the model design. We ultimately did not incorporate them into this dynamic model trained on a monthly basis, mainly due to the following two reasons:

Mismatch in time scales: Road density is a static or quasi-static spatial background factor with an extremely long change cycle (on an annual basis). However, the core of this study is to capture the monthly-scale dynamic changes in nitrogen dioxide concentrations, whose driving factors are mainly dynamic variables such as meteorological conditions.

We have added the limitation regarding our decision not to include human factors in the discussion section of the paper. Thank you for your valuable comments.(L531-L539)

**(4)Reviewer 1 also stresses the need to clarify how cloud cover and seasonal variability in TROPOMI sampling were handled, since differences in observation counts across seasons may bias annual averages.**

**Response:** Thank you for the editor's reminder. Regarding this issue, we have already provided a detailed response in the point-by-point replies to the reviewer's comments below. These responses include adding persuasive references and supplementing explanations of limitations in the Discussion section, among other measures.

**(5)Reviewer 2 similarly notes that the characterization of pollution sources remains insufficient and requests that the manuscript explicitly state the use of tropospheric NO₂ data rather than total column values. They further recommend integrating methodological details more directly into the discussion, including justification for the use of Random Forest, explanation of the training/validation split, and reflection on the strengths, limitations, and parameter choices of the model.**

**Response:** Regarding the issue of pollution sources, we have provided a detailed explanation and response to Reviewer 2.

As for the question of whether it refers to nitrogen dioxide concentration or vertical column concentration, we have redefined the term and thoroughly checked the manuscript to ensure terminological consistency.

Additionally, we have added the advantages of the random forest algorithm and the reasons for its selection to both the Discussion section and the Methodology section.(L231-L248, L464-L470)

**(6)Finally, Reviewer 2 points out the absence of independent validation against monitoring data or emissions inventories, which should at least be discussed as a limitation.**

**Response:** The reviewer is absolutely correct. The lack of validation using independent monitoring data or emission inventories is a significant limitation of our study, and we should discuss this in the paper. We sincerely apologize for this previous oversight. To actively respond to your suggestion, we have added a separate paragraph in the Discussion section of the paper specifically addressing this limitation. In this paragraph, we:

Clearly state that the lack of independent validation is one of the core limitations of this study;

Suggest that future research should prioritize establishing a ground-based monitoring network in this region or developing a refined emission inventory to calibrate and validate the analytical results of such satellite data.(L539-L545)

**Concluding Response to the Editor:**

Thank you to the editor for consolidating the reviewers' comments and highlighting key points for our attention. We will carefully respond to each comment one by one and revise the manuscript to enhance its quality.

**Authors’ Response to Reviewer 1**

**Major Comment 1**

“The study primarily examines meteorological and vegetation factors, as these constitute the dominant influences within our research framework.”

**To me though this could be attributable to exclusion bias, since there aren’t anthropogenic variables included you can’t really know the effect of them on your predictions without trying them.**

**Response:** We greatly appreciate you raising this valuable comment. You have correctly identified a major limitation of our study: the model fails to include direct human activity variables, which may indeed introduce exclusion bias and affect our precise interpretation of the prediction results.

We frankly acknowledge that due to limitations in the time scale of the data, we were unable to incorporate these factors into the current analysis. This indeed means that the patterns identified by our model may be a mixture of the effects of natural factors and unobserved human factors.

To proactively address your concern, we have added a description of the study's limitations in the discussion section of the paper. We specifically discuss how the omission of these variables might bias our estimates—for instance, it may lead to an overestimation of the effects of natural factors.

While this limitation exists, we believe that within the scope permitted by the available data, this study still provides valuable insights into the spatiotemporal analysis of nitrogen dioxide and its influencing factors. Your critique is of crucial importance to us, as it helps us more clearly define the boundaries and contributions of this research.（L531-L539）

**Major Comment 2**

“The most recent publicly available demographic data currently extends only to 2022, while our research period focuses on 2023. This temporal misalignment would compromise the analytical robustness of our findings."

**This is incorrect. There are multiple population datasets that project to present day (e.g., WorldPop** [**https://hub.worldpop.org/geodata/listing?id=137**](https://hub.worldpop.org/geodata/listing?id=137)**)**

**Response:** We sincerely appreciate you pointing out this error and providing such specific and valuable resources (WorldPop).

We have conducted an in-depth study of the WorldPop dataset you recommended. It is an extremely excellent data product. However, we found that its latest version (GPWv4.11) is also mainly based on annual interpolated data from census years, with a temporal resolution of yearly. The training and prediction of our model are based on a monthly basis. Directly incorporating static annual data that remains unchanged within a year cannot help us capture and explain the monthly variations in human activities during the study period (e.g., 2010-2020). Therefore, we believe that while static annual population data is inherently crucial, it cannot be incorporated into the model in this study, which focuses on monthly influencing factors. However, in response to your comment, we will address the limitations of human activity factors in the discussion section.

In conclusion, we would like to express our special gratitude to you for pointing out the valuable resource of WorldPop, which will provide us with precious data support in future research.(L531-L539)

**Major Comment 3**

“In this article, we will appropriately cut out the comments on anthropogenic factors and focus on discussing the effects of natural factors on NO_2_ concentrations.We sincerely appreciate your insightful suggestions, which have strengthened our manuscript's scholarly rigor.”

**I think this again suffers from exclusion bias. If you’re missing major characteristics associated with NO**_2_ **pollution that aren’t reflected in your predictor variable dataset then this could distort the importance of the predictor variables. This is especially important given your focus on urban environments in which anthropogenic sources of NO**_2_ **dominate. I think that some aspect of anthropogenic NOx must be considered in your model to accurately reflect surface NO**_2_ **in urban environments. I am glad that built environment is captured in impervious area; however, I suggest that some aspect associated with roads is included. Perhaps just the major roads / highways for this work and then you can consider how the inclusion of greater fine detail affects the model performance in future studies.**

**Response:** Thank you very much for your valuable suggestions. Your proposal to introduce the road density factor to improve the model is a highly insightful perspective, and it is indeed of great importance for capturing the spatial distribution pattern of pollutants.

After carefully considering your suggestion, we also conducted in-depth discussions on such static spatial factors during the initial stage of the model design. We ultimately did not incorporate them into this dynamic model trained on a monthly basis, mainly due to the following two reasons:

Mismatch in time scales: Road density is a static or quasi-static spatial background factor with an extremely long change cycle (on an annual basis). However, the core of this study is to capture the monthly-scale dynamic changes in nitrogen dioxide concentrations, whose driving factors are mainly dynamic variables such as meteorological conditions.

We have added the limitation regarding our decision not to include human factors in the discussion section of the paper. Thank you for your valuable comments.（L531-L539）

**Major Comment 4**

“You have mentioned this issue multiple times, which indicates its significance. We

would like to explain it again: It is true that cloud cover can affect the accuracy of the data.

However, the impact of clouds is more pronounced at the monthly time scale, while our study adopts seasons and years as the time scales for research. Therefore, its influence on the results of this study is relatively minor. Thank you again for your professional suggestions. We are happy to provide further explanations if needed.”

**I think this response fails to address my original point. To be clear, I am wondering how homogenization is done because in certain seasons there is a greater proclivity for cloud coverage which can affect how many observations TROPOMI has. For example, if cloud coverage leads to only 40 observations in the winter but there are 80 in the summer if you average the two to calculate the annual average, than these results will more reflect summertime TROPOMI NO_2_ than annual.**

**Response:** You are absolutely correct to point out that when averaging data affected by cloud cover—such as TROPOMI data—if there are significant seasonal differences in the number of observations, a simple arithmetic mean will introduce severe representational bias, skewing the results toward seasons with more favorable observation conditions. We greatly appreciate your help in enhancing the rigor of the methodology used in this study.

1. Strict Quality Control:

We did not use all TROPOMI L2 observation data; instead, we used the L3 level data. In accordance with official recommendations, we set a strict quality assurance value threshold and only retained observation results with high confidence under cloud-free or clear-sky conditions. This step itself has greatly reduced the problem of observation gaps caused by cloud cover, providing us with a higher-quality and more consistent dataset for subsequent averaging calculations.

2. Reasonableness of Monthly Averages:

After applying the aforementioned quality control, we generated monthly average data. Although the number of valid observations in winter is still fewer than that in summer, the quality of the remaining valid observations is comparable across seasons, as low-quality observations have been excluded. Therefore, the monthly arithmetic mean is a reasonable and standard method to characterize the monthly NO₂ levels.

3. Methodology for Calculating Annual Averages (Key Improvement):

We fully agree with your view that caution is required when calculating annual averages.

First, based on the aforementioned high-quality monthly data, we calculated the average value for each season.

Then, we further computed the arithmetic mean of the four seasonal averages to obtain the final annual average.

The interannual variation boxplot has been replaced, and the updated figure can be seen below.(Fig. 3) (L183-L188 )

Meanwhile, we found that the interannual spatial variation map had a low degree of visualization. To highlight the key content, we removed this map and focused on discussing the spatial distribution and agglomeration status of TNO₂CC through the local spatial autocorrelation analysis map.

The core advantage of this method lies in the fact that it assigns equal weight to each season (25% each), thereby ensuring that the annual average can fairly represent the situation throughout the year and will not be dominated by any season with a larger number of observations. This is a widely adopted geostatistical method used to correct for uneven observation frequencies.

In response to your comment, we have more clearly elaborated on this calculation process of "first calculating seasonal averages, then annual averages" in the Methods section (Statistical Analysis) of the paper, and emphasized its importance in avoiding seasonal observation bias. ( L183-188)

**Concluding Response to the Editor:**

First, we apologize for the omissions in our first response to your suggestions. Second, in response to your comments, we have provided detailed explanations and revisions this time, hoping to meet your requirements. Finally, thank you for reviewing our manuscript a second time amid your busy schedule. Best regards.

**Authors’ Response to Reviewer 2**

**Major Comment 1**

The manuscript also doesn't make clear whether they worked specifically with tropospheric NO_2_ data or used the entire column, although in their response to the reviewer, they state that they did use the tropospheric NO_2_ fraction.

**Response:** We greatly appreciate this valuable comment from the reviewer. You are absolutely correct, and we sincerely apologize for the oversight in the manuscript where we failed to clearly distinguish between "concentration" and "column concentration". This is a crucial conceptual difference, and precise terminology is essential to avoid misunderstandings among readers. To fully address this issue, we have taken the following measures:

Comprehensive Review: We conducted a systematic screening of the entire manuscript to identify and correct all ambiguous expressions.

Unified Terminology: When referring to satellite data such as TROPOMI, we have revised the term "concentration" to the more scientific " tropospheric column concentration of nitrogen dioxide (TNO_2_CC) ".( Take the green-font part of the abstract as an example)

Clear Definition: We added a sentence in the data source section to clearly define the data used in this study: "All satellite nitrogen dioxide data used in this study are tropospheric column concentration, with a unit of mol/m²."( above Table 1,L126-128)

**Major Comment 2**

Formally, the response to most of the observations is subject to the deletion of some arguments that lack supporting information, adjustments to the wording of statements, the reorganization of ideas, the incorporation of some graphic elements, the inclusion of new references, and explanations that, in some cases, don't mention the position where the change was made (but it is important that they be included in the document.

**Response:** They mainly cover the following aspects:

1. Refinement and Focus of Arguments

We re-evaluated all arguments and removed several statements that lacked sufficient data or reference support, ensuring that every claim in the manuscript is solid and robust.(L745-L769)

2. Polishing of Sentences and Wording

We polished the language throughout the entire manuscript and adjusted ambiguous or inaccurate wording to enhance the precision and professionalism of the expression.
**Major Comment 3**

On the other hand, if we use a standard approach, I think this work could be improved if the authors incorporated elements directly related to the methodological criteria they used for data analysis into the discussion. For example, they mention that they used a 2/3 and 1/3 data ratio to train and validate the Random Forest model, respectively; but no details are offered in the discussion about why this algorithm was used, its advantages and/or disadvantages, complications associated with changing algorithm parameters, etc. While training and validation yield acceptable correlations, there is no data from other sources (monitoring systems or emissions inventories) that validate estimates based solely on satellite data.

**Response:** Regarding the issue of data validation, thank you for pointing this out. This is indeed a limitation of our paper, and we have already summarized the limitations of this study in the Discussion section(L539-L549).Additionally, the advantages of the Particle Swarm Optimization (PSO) algorithm and the reasons for its selection have also been supplemented in the Discussion section; please refer to it for details.（L231-L248,L464-L470）

**Concluding Response:** We are deeply honored to receive your review comments again. The suggestions you put forward this time are equally profound and pertinent, directly addressing the key aspects of the manuscript, and we would like to express our most sincere gratitude for this.

We are well aware that our response to the initial draft still had shortcomings. This time, with the utmost sincerity, we have conducted repeated discussions on each of your comments and made careful revisions, striving to fully address your concerns. Your guidance has benefited us greatly and has also helped to further improve this study.
