# Supplementary material for: Nitrogen dioxide pollution in 346 Chinese cities: Spatiotemporal variations and natural drivers from multi-source remote sensing data
Source: PLoS One. 2025 Nov 7;20(11):e0334535. doi: 10.1371/journal.pone.0334535 (PMC12594365; doi:10.1371/journal.pone.0334535)
Supplement: Attachment — Submitted filename: Response_to_Reviewers_auresp_3.docx [file pone.0334535.s010.docx]

Responses to Reviewers’ Comments for Manuscript PONE-D-25-30016R2

**Nitrogen dioxide pollution in 346 Chinese Cities: Spatiotemporal variations and drivers from multi-source remote sensing data**

Addressed Comments for Publication to

PLOS ONE

by

Heming Yang

Dear Dr. Editor,

We greatly appreciate your kind letter and positive feedback, which have been a great source of encouragement for us. We will definitely take each comment from the review experts seriously, revise the manuscript meticulously, and strive for perfection. We look forward to the smooth publication of the manuscript under your guidance.

Please find enclosed the revised version of our previous submission entitled “Nitrogen dioxide pollution in 346 Chinese Cities: Spatiotemporal variations and natural drivers from multi-source remote sensing data” with manuscript number PONE-D-25-30016R2. We would like to thank you and the reviewers for the valuable comments which help improving the quality of our manuscript.

In this revision, we have carefully addressed the reviewers’ comments. Provided below is a summary of the main modifications, along with our responses to the reviewers’ comments.

Sincerely,

Heming Yang

**Authors’ Response to the Editor**

**(1) Accordingly, the reviewer asks for clarification on two key points: Inclusion of static/human-related data: If the authors wish to maintain their approach of not including this type of information, a stronger justification is required to explain why this methodological decision is valid and what implications it has for the results.**

**Response:** We would like to thank the editor for summarizing the comments raised by the reviewers amid your busy schedule, as well as for putting forward the important point regarding the inclusion of static/human-related factors. We have carefully considered this suggestion. We also appreciate the reviewers for providing valuable feedback on the potential role of static and human-related data. This is indeed a relevant consideration for many studies in this field. In lines 526-530 of the manuscript, we cite previous studies to illustrate the significance of human factors, which is identified as a key focus for subsequent research.

**(2) Alignment of the manuscript: If human-centric or static data are not to be included, the title, abstract, and motivation of the paper should be revised to accurately reflect the scope of the study and avoid giving the impression that such aspects are integrated.**

**Response:** After thorough deliberation, we have decided to maintain our current approach, which primarily focuses on the impact of natural factors on changes in NO₂ concentrations in air pollution. Based on this, we have refined the title, abstract, and research motivation of the paper to accurately reflect the research scope and define its boundaries. For the specific details of the revisions, please refer to the responses to Reviewer 1’s comments provided below.

**Concluding Response to the Editor:**

Thank you to the editor for consolidating the reviewers' comments and highlighting key points for our attention. We will carefully respond to each comment one by one and revise the manuscript to enhance its quality.

**Authors’ Response to Reviewer 1**

**Major Comment 1**

I thank the authors for responding to my second round of comments. I am not sure I follow your argument involving static vs dynamic data in your model. As I understand it, adding the static data would not worsen the model performance and it would introduce currently missing features in the prediction. Some aspects of surface-level NO_2_ are well correlated with static features. I am guessing it would be more beneficial to have static or annual only human activity data in your model than none at all, or do you disagree? If you do not want to include human centric data in your model I believe that you should reframe the title, abstract, and motivation of this paper to accurately reflect this.

**Response:** We would like to express our sincere gratitude again for investing your time in the second round of review and providing such insightful and constructive comments. Your questions get straight to the core, and they have also helped us realize that we may not have fully clarified the most fundamental theoretical basis of this study in our previous response. We fully understand your perspective and agree with it.

Please allow us to explain that the primary objective of this study is to conduct a "controlled mechanism analysis." Therefore, we have decided to adopt your second suggestion: to define the overall scope of the study as the impact of natural factors on NO₂ concentrations in air pollution. We have thoroughly reviewed the manuscript from start to finish and revised sections where the distinction between natural and anthropogenic factors was unclear, including the title, abstract, and the section on influencing factors.

We earnestly request that you take a moment from your busy schedule to review our revised manuscript, with the hope that it meets your requirements. For the specific details of the revisions, please refer to the content below. Thank you again for your valuable comments.

1. In lines 526-530 of the manuscript, we cite previous studies to illustrate the significance of human factors, which is identified as a key focus for subsequent research.

2.Define in the Title: Change“drivers” to“natural drivers”.

3. Since we have adopted your suggestion to define the influencing factors as natural influencing factors, we have carefully reviewed the manuscript and deleted all content related to the impact of human factors.

4. "natural" is added to define "influencing factors" in all other sections. This can be seen in L21, L23, L25, L31, L33, L75, L270 L282 L294 L401 L404 L409, L417, L421, L427, L465, L468, L470, L473, L484, L489, L515, L529, L557.

5.Since our focus is on the influence factor analysis mechanism rather than concentration prediction, descriptions related to "predictor variable" have been removed and replaced with "variable".

Concluding Response: We are deeply honored to receive your review comments once again. The suggestions you have put forward on this occasion are equally insightful and to the point, accurately identifying the key issues in the manuscript, and we would like to express our most sincere gratitude for this.

We are well aware that our previous response to the second draft still had shortcomings. This time, with the utmost sincerity, we have conducted in-depth and repeated discussions on your comments, made meticulous revisions, and strived to fully address your concerns. Your guidance has benefited us greatly and has also helped to further refine this study.
